# Exploring the causal relationship between bipolar disorders and sensory, motor, and behavioral disorders: A bidirectional Mendelian randomization analysis

**DOI:** 10.1097/MD.0000000000044056

**Published:** 2025-08-22

**Authors:** Yuyan Chen, Yan Zhang, Bangqi Wu, Yupei Cheng, Jingjie Huang, Chaoran Wang, Jing Bai, Yuxing Zhang

**Affiliations:** a First Teaching Hospital of Tianjin University of Traditional Chinese Medicine, National Clinical Research Center for Chinese Medicine Acupuncture and Moxibustion, Tianjin, China; b Tianjin University of Traditional Chinese Medicine, Tianjin, China.

**Keywords:** behavioral disorders, bipolar disorder, comorbidities, Mendelian randomization, motor disorders, sensory disorders

## Abstract

The interrelationships between bipolar disorder (BD) and sensory, motor, and behavioral disorders are intricate and not well defined. While observational studies indicate potential associations, causal relationships have not been established. This study applies bidirectional Mendelian randomization (MR) analysis to examine potential causal links between BD and these disorders. Bidirectional MR analysis uses summary-level data from genome-wide association studies (GWAS). Genetic instruments are selected according to stringent significance thresholds and linkage disequilibrium (LD) criteria. The primary analytical approaches include inverse-variance weighted (IVW) MR and MR-Egger regression. Instrument strength is assessed using *F*-statistics. Significant bidirectional associations are identified. BD is associated with increased genetic susceptibility to pruritus (OR = 1.29, 95% CI: 1.06–1.57), small fiber neuropathy (OR = 1.64, 95% CI: 1.03–2.61), hyperkinetic disorders (OR = 2.02, 95% CI: 1.26–3.23), anorexia nervosa (OR = 1.19, 95% CI: 1.01–1.40), and autism spectrum disorder (OR = 1.10, 95% CI: 1.01–1.20). Conversely, pruritus and psoriasis are identified as significant genetic risk factors for BD (pruritus: OR = 1.04, 95% CI: 1.01–1.08; psoriasis: OR = 9.97, 95% CI: 1.85–53.67). Motor disorders are associated with a protective effect against BD (OR = 0.88, 95% CI: 0.80–0.96). This study demonstrates significant bidirectional causal associations between BD and sensory, motor, and behavioral disorders, underscoring the importance of early screening and integrated clinical management. Shared genetic and neurobiological mechanisms may inform the development of targeted interventions and therapeutic strategies.

**Key Points**

1. Robust bidirectional MR analysis is conducted to identify potential causal associations between BD and sensory, motor, and behavioral disorders.
2. Sensory abnormalities, particularly pruritus, are identified as potential early indicators of BD.
3. Shared genetic and neurobiological mechanisms are observed between BD and its comorbidities – including pruritus, small fiber neuropathy, hyperkinetic disorders, anorexia nervosa, and autism spectrum disorder – offering insights for developing precision medicine strategies in BD management.

## 1. Introduction

Bipolar disorder (BD), formerly referred to as manic-depressive illness) is a chronic, recurrent psychiatric condition, typically characterized by alternating episodes of mania and depression, accompanied by underlying biological, psychological, and other functional disturbances, resulting in substantial distress or disruption to daily functioning.^[1,2]^ Descriptions of BD-like symptoms date back to the Middle Ages, with 19th-century psychiatrists Jules Baillarger and Jean-Pierre Falret formally identifying this phenomenon, introducing the concepts of “dual-form psychosis” and “circular psychosis” respectively.^[3–6]^ In the 20th century, psychiatrist Emil Kraepelin incorporated these early observations into the diagnosis of “manic-depressive illness” and advanced the classification of BD subtypes.^[3,4,6]^ Bipolar I disorder is defined by manic episodes lasting at least 1 week, typically accompanied by major depressive episodes; in contrast, bipolar II disorder involves hypomanic episodes and major depressive episodes lasting at least 4 days, without full manic episodes.^[7–9]^

BD ranks 17th among all disease-related disabilities globally and exerts a substantial impact on health systems and social structures worldwide.^[10]^ Epidemiological data indicate a lifetime prevalence of 2.4% and a 12-month prevalence of 1.5% for BD,^[11]^ with significantly higher rates observed in high-risk groups, particularly those with a family history or early-onset mood disorders.^[2]^ The disorder typically emerges during adolescence or early adulthood, characterized by cyclical mood episodes that profoundly impair daily functioning, interpersonal relationships, and occupational performance.^[12]^ Moreover, the suicide risk for individuals with BD is much higher than in the general population, with approximately 6% to 7% of BD patients dying by suicide,^[13,14]^ and the overall suicide rate estimated to be 20 to 30 times higher than that of the general population, underscoring the disorder’s significant mental health burden.^[13,14]^ Beyond personal suffering, BD imposes substantial socioeconomic burdens, including frequent medical consultations, long-term dependency on healthcare services, and challenges in treatment due to high comorbidity rates. In addition, the disorder contributes to reduced productivity, increased unemployment risk, and disrupted family and social functioning, all of which have far-reaching economic and societal consequences.

It is important to note that BD is closely associated with a range of sensory, cognitive, and behavioral impairments. Recent studies have gradually unveiled the complex relationships between BD and sensory disturbances, including abnormalities in cutaneous sensation, olfaction, and auditory perception, as well as motor dysfunction and behavioral comorbidities such as eating disorders, hyperkinetic disorders, and substance abuse. Observational data suggest that BD is significantly linked with sensory system dysfunction, with individuals frequently reporting abnormal cutaneous sensitivity, particularly chronic pain.^[15]^ Additionally, deficits in sensory gating have been observed in BD patients,^[16]^ alongside alterations in gustatory and olfactory functions, which may further compromise daily quality of life and social functioning.^[17–20]^ Additional findings highlight comorbid motor dysfunction, particularly among stroke survivors,^[21]^ and a high prevalence of BD in individuals with multiple sclerosis, potentially driven by shared neurobiological pathways.^[22]^ Structured physical activity has been shown to ameliorate mental and physical health outcomes in BD patients.^[23,24]^ Notably, BD is also comorbid with a variety of behavioral disorders such as hyperkinetic disorders, anorexia nervosa, ASD, and substance abuse. Hyperkinetic disorders are frequently co-diagnosed with BD and exert a significant negative impact on illness trajectory and prognosis^[25]^; 11.3% of anorexia nervosa patients are suspected of having BD, which complicates treatment and worsens the overall effect on their lives.^[26]^ Similarly, BD is highly prevalent among adolescents and young adults with high-functioning autism spectrum disorder (ASD),^[27]^ and this comorbidity is regarded as a particularly severe psychiatric condition in youth.^[28,29]^ These converging lines of evidence underscore BD’s pathophysiological mechanisms’ multifaceted and heterogeneous nature. Therefore, a more nuanced understanding of its comorbidities with sensory, motor, and behavioral disorders is crucial for elucidating underlying biological mechanisms. It may provide essential guidance for comprehensive clinical evaluation and personalized therapeutic approaches. Although existing research has explored the associations between BD and various sensory, motor, and behavioral disorders, causal relationships remain insufficiently characterized, and our understanding of these complex interactions is still limited.

Mendelian randomization (MR) is a powerful analytical approach for inferring causality in epidemiological research. When randomized controlled trials (RCTs) are impractical, MR leverages genetic variants as instrumental variables (IVs) to estimate the relationship between exposures and outcomes in non-experimental data, thus providing potential causal evidence.^[30,31]^ The theoretical foundation of MR lies in Mendel laws of inheritance, which state that alleles are randomly assorted during gamete formation. This random allocation of genes minimizes confounding, measurement error, and reverse causality inherent in traditional multivariable regression methods, enhancing the credibility of causal inferences.^[32,33]^ MR analysis can be conducted using either a one-sample or two-sample approach. In one-sample MR, genetic variation, risk factors, and health outcomes are measured in the same participant group. In contrast, in two-sample MR, genetic variation and health outcomes are measured in one sample, and genetic variation and risk factors are measured in another.^[34]^ A key advantage of the two-sample approach is its ability to reduce the risk of false-positive findings due to sample overlap. Critically, bidirectional MR enables the reversal of exposure and outcome roles, offering a framework to investigate potential reverse causality. Given the substantial public health implications of understanding the bidirectional relationships between BD and sensory, motor, and behavioral disorders, establishing robust causal evidence is essential for guiding effective prevention and intervention strategies.

The primary objective of this study is to apply bidirectional MR to assess the potential causal relationships between BD and a range of sensory, motor, and behavioral disorders, thereby enhancing our understanding of their complex etiological interplay. Specifically, this study plans to use data from the IEU genome-wide association studies (GWAS) database, FinnGen, UK Biobank (UKB), and the Psychiatric Genomics Consortium (PGC) to assess the potential causal links between BD and sensory, motor, and behavioral disorders. This analysis not only aims to reveal the possible influence of BD on sensory, motor, and behavioral disorders but also explores the potential causal effects of these disorders on BD. By employing this bidirectional MR approach, more robust causal inferences can be obtained, providing a theoretical basis for developing relevant preventive and intervention strategies, thus improving the effectiveness and specificity of treatment.

## 2. Methods

### 2.1. Study design

Our current MR study adopted a bidirectional MR framework, utilizing IVs derived from 14 GWAS to explore causal relationships. We first extracted single-nucleotide polymorphisms (SNPs) associated with the exposure traits from relevant GWAS datasets to identify potential exposure-related SNPs. Subsequently, we retrieved corresponding SNPs for the outcome traits from independent GWAS sources and verified their availability and alignment. SNPs that met predefined quality control and linkage disequilibrium criteria were retained. Then, we applied multiple MR methods to comprehensively evaluate the causal effect of the exposure traits on the risk of outcome traits. To further assess the directionality of the observed associations, we reversed the analytical direction, treating outcomes as exposures and vice versa, to investigate potential reverse causality.

### 2.2. Data source

Genetic summary statistics for BD were obtained from the PGC, comprising 41,917 BD cases and 371,549 controls, totaling 413,466 participants of European ancestry. Genetic data for sensory, motor, and behavioral disorders were primarily derived from the IEU GWAS database, FinnGen, UK Biobank (UKB), and PGC. Hearing impairment data were sourced explicitly from a large-scale GWAS conducted by Kalra et al, which analyzed hearing loss in 330,759 individuals of European descent and is available in the GWAS Catalog.^[35]^ All datasets were accessed through the OPEN GWAS platform (https://gwas.mrcieu.ac.uk/). Since all included GWAS data were publicly available and had received ethical approval from the original studies’ institutional review boards, no additional ethics approval was required for the present analysis. Covariate adjustments (e.g., age, sex, population structure) were performed in the original GWAS analyses. The MR analysis was conducted using summary-level data; therefore, no further covariate adjustment was necessary.

### 2.3. Instrument selection criteria

In this MR study, we selected genome-wide SNPs (*P* < 5 × 10^−8^) for each exposure from publicly available GWAS datasets. We ensured that these SNPs were in linkage equilibrium (*r*^*2*^ < 0.001) with other SNPs within a 10,000-kb region so that they could serve as genetic instruments for the study. To ensure the validity of sensitivity analyses, at least 3 SNPs associated with the exposure factors were required to serve as genetic tools. Therefore, if the number of eligible SNPs was fewer than 3, the P threshold was adjusted to 5 × 10^−6^. A summary of the SNP data used in this MR study is provided in Supplementary File 1 (Supplemental Digital Content, https://links.lww.com/MD/P738).

### 2.4. Statistical analysis

All MR analyses were conducted using R (version 4.4.1, www.r-project.org/) with the DevTools, TwoSampleMR, and MR-PRESSO packages.^[36]^ The inverse-variance weighting (IVW) method was used as the primary approach to assess the causal associations between BD and sensory, motor, and behavioral disorders. Cochran *Q* test was performed to evaluate heterogeneity across IVs.^[37]^ When significant heterogeneity was observed (*P* < .05), the MR Pleiotropy RESidual Sum and Outlier (MR-PRESSO) method was implemented to identify and remove outlier SNPs, thereby accounting for heterogeneity and improving estimate robustness. Following outlier removal, the MR analysis was re-estimated to obtain refined causal estimates. If heterogeneity remained (*P* < .05), a random-effects IVW model was used; otherwise, a fixed-effects IVW model was applied.^[38]^ MR-Egger regression was used to assess potential horizontal pleiotropy through the intercept term. If the P was < 0.05, it indicated the presence of pleiotropic effects, which were then used to evaluate the relationships between SNPs.^[39]^ For binary exposures, causal effects were reported as odds ratios with 95% confidence intervals per unit log-odds increase in genetically predicted exposure. Additionally, a leave-one-out analysis was performed as a sensitivity analysis, where 1 SNP was sequentially removed in each iteration of the MR analysis to test for bias arising from specific SNPs. *F*-statistics were calculated using the formula *F* = Beta^2^/ SE^2^ to mitigate weak instrument bias. An *F*-statistic > 10 indicated a strong instrument, reducing the risk of weak instrument bias in MR estimates.^[40]^ Each MR analysis between BD and a specific trait was treated as an independent, hypothesis-driven test; therefore, no multiple testing correction was applied.

## 3. Results

Based on GWAS, this MR analysis investigated the potential causal relationships between BD and 13 sensory, motor, and behavioral disorders (Table 1). Supplementary File 1 (Supplemental Digital Content, https://links.lww.com/MD/P738) provides detailed information on the IVs for each exposure factor.

**Table 1:** Descriptions of GWAS data used for analyses.

Phenotype	Study or Consortium	Sample size	Cases, n	Controls, n	Population
Bipolar disorders	PGC	413,466	41,917	371,549	European
Sensory-related disorders					
Pain in the limb	Neale lab	361,194	3674	357,520	European
Pruritus	FinnGen	200,110	1370	198,740	European
Hearing impairment	Kalra G	330,759	NA	NA	European
Tinnitus	Neale Lab	109,411	7214	102,197	European
Anosmia	FinnGen	60,206	379	59,827	European
Small fiber neuropathy	FinnGen	215,961	243	215,718	European
Psoriasis	MRC-IEU	462,933	5314	457,619	European
Motor and behavioral disorders					
Extrapyramidal and movement disorders	FinnGen	218,792	4948	213,844	European
Multiple Sclerosis	MRC-IEU	462,933	1679	461,254	European
Hyperkinetic disorders	FinnGen	216,008	245	215,763	European
Anorexia nervosa	PGC	14,477	3495	10,982	European
Autism spectrum disorder	PGC	46,351	18,382	27,969	European
Psychoactive substance abuse	MRC-IEU	463,010	11,793	451,217	European

GWAS = genome-wide association studies, MRC-IEU = Medical Research Council Integrative Epidemiology Unit, PGC = Psychiatric Genomics Consortium.

### 3.1. MR results of BD on the risk of sensory, motor, and behavioral disorders

**Figure 1.**
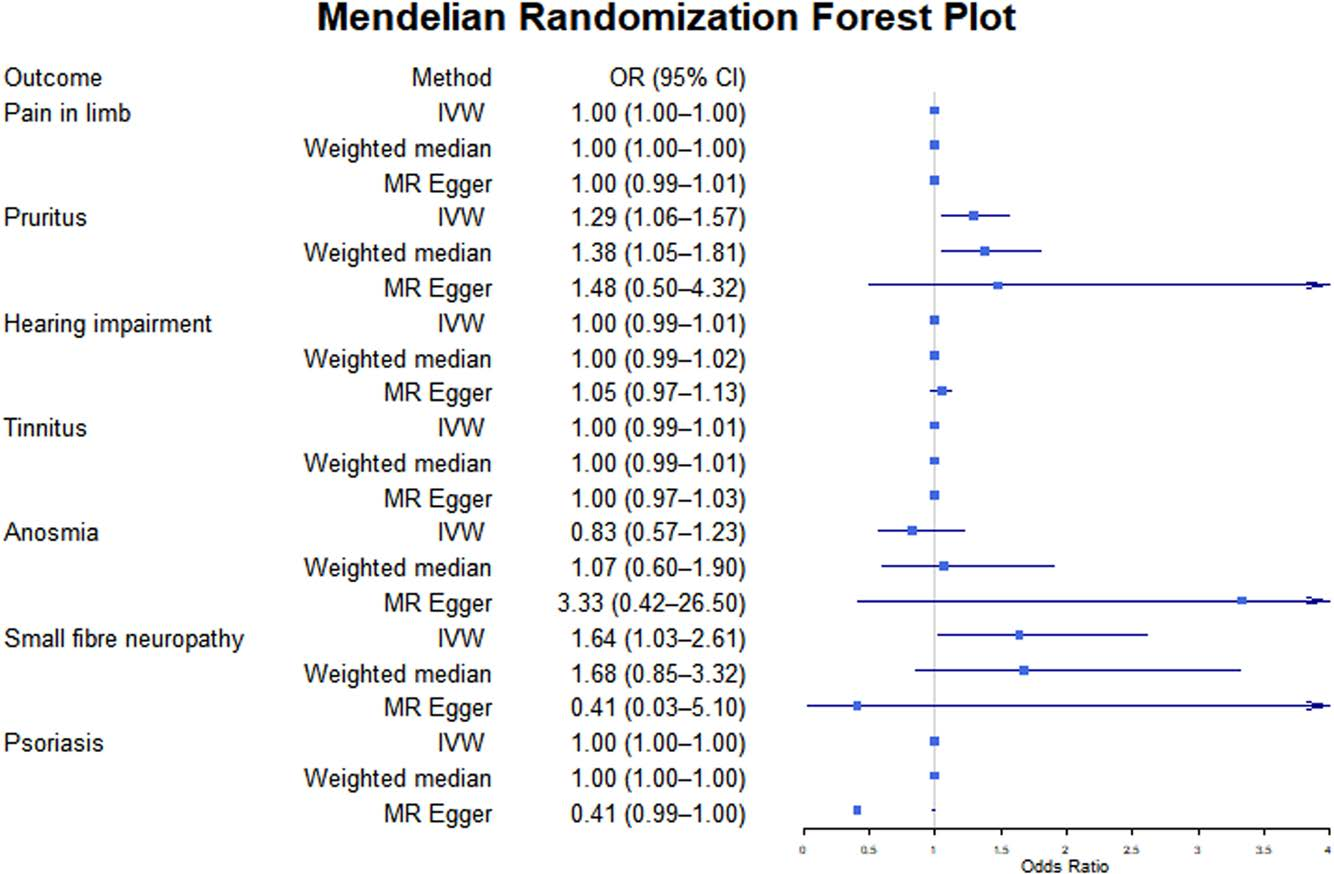
The putative causal effect of bipolar disorders on sensory disorders. The results from Mendelian randomization analysis using the IVW approach, the weighted median approach, or the MR-Egger approach (when horizontal pleiotropy exists). Circles and horizontal bars represent the odds ratios and confidence intervals of the factor with the risk of pain, respectively. CI = confidence interval, IVW = inverse-variance weighted approach, MR = Mendelian randomization, OR = odds ratio.

**Figure 2.**
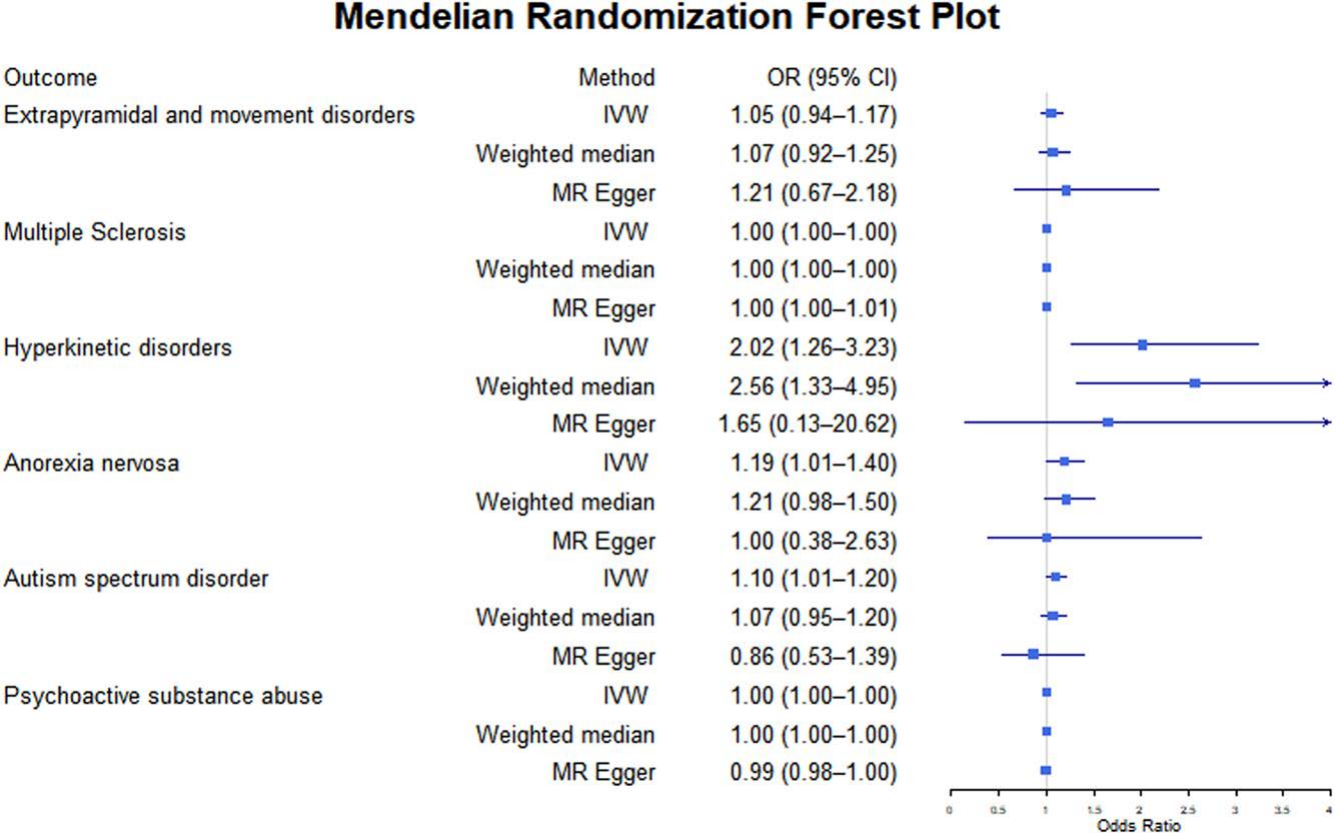
The putative causal effect of bipolar disorders on motor and behavioral disorders. The results from Mendelian randomization analysis using the IVW approach, the weighted median approach, or the MR-Egger approach (when horizontal pleiotropy exists). Circles and horizontal bars represent the odds ratios and confidence intervals of the factor with the risk of pain, respectively. CI = confidence interval, IVW = inverse-variance weighted approach, MR = Mendelian randomization, OR = odds ratio.

As shown in Tables 2 and 3) and Figures 1 and 2, BD is significantly associated with an increased risk of several sensory, motor, and behavioral disorders. BD is a significant risk factor for pruritus (OR = 1.29, 95% CI: 1.06–1.57, *P* = .01) and small fiber neuropathy (OR = 1.64, 95% CI: 1.03–2.61, *P* = .04). Additionally, BD significantly increased the risk of attention-deficit/hyperactivity disorder (hyperkinetic disorders) (OR = 2.019, 95% CI: 1.263–3.229, *P* = .003), anorexia nervosa (OR = 1.19, 95% CI: 1.01–1.40, *P* = .04), and ASD (OR = 1.10, 95% CI: 1.01–1.20, *P* = .03).

**Table 2:** The univariate MR results of bipolar disorders on the risk of sensory disorders.

Outcome	Method	Bipolar disorders		
		*N* SNV	OR (95%CI)	*P* value
Pain in the limb	IVW	51	1.00 (95% CI: 0.999–1.001)	.92
	Weighted median	51	1.00 (95% CI: 0.999–1.002)	.72
	MR-Egger	51	1.00 (95% CI: 0.993–1.007)	.94
Pruritus	IVW	49	1.29 (95% CI: 1.06–1.57)	.01
	Weighted median	49	1.38 (95% CI: 1.05–1.81)	.03
	MR-Egger	49	1.48 (95% CI: 0.50–4.32)	.48
Hearing impairment	IVW	41	1.00 (95% CI: 0.99–1.01)	.92
	Weighted median	41	1.00 (95% CI: 0.99–1.02)	.72
	MR-Egger	41	1.05 (95% CI: 0.97–1.13)	.28
Tinnitus	IVW	40	1.00 (95% CI: 0.99–1.01)	.98
	Weighted median	40	1.00 (95% CI: 0.99–1.01)	.76
	MR-Egger	40	1.00 (95% CI: 0.97–1.03)	.83
Anosmia	IVW	42	0.83 (95% CI: 0.57–1.23)	.36
	Weighted median	42	1.07 (95% CI: 0.60–1.90)	.82
	MR-Egger	42	3.33 (95% CI: 0.42–26.50)	.26
Small fiber neuropathy	IVW	42	1.64 (95% CI: 1.03–2.61)	.04
	Weighted median	42	1.68 (95% CI: 0.85–3.32)	.14
	MR-Egger	42	0.41 (95% CI: 0.03–5.10)	.49
Psoriasis	IVW	50	1.00 (95% CI: 0.999–1.002)	.70
	Weighted median	50	1.00 (95% CI: 0.998–1.001)	.82
	MR-Egger	50	0.41 (95% CI: 0.988–1.003)	.27

Genetic instruments selected from bipolar disorders GWASs, selection threshold *P* less than 5 × 10^−8^, pruned at linkage disequilibrium *R*^2^ <0.001 (10,000 kb pair window).

CI = confidence interval, GWAS = genome-wide association studies, IVW = inverse-variance weighted, MR = Mendelian randomization, *N* SNV = number of single-nucleotide variants, OR = odds ratio.

**Table 3:** The univariate MR results of bipolar disorders on the risk of motor and behavioral disorders.

Outcome	Method	Bipolar disorders		
		*N* SNV	OR (95%CI)	*P* value
Extrapyramidal and movement disorders	IVW	42	1.05 (95% CI: 0.94–1.17)	.40
	Weighted median	42	1.07 (95% CI: 0.92–1.25)	.37
	MR-Egger	42	1.21 (95% CI: 0.67–2.18)	.53
Multiple Sclerosis	IVW	35	1.000 (95% CI: 0.999–1.001)	.68
	Weighted median	35	1.001 (95% CI: 1.000–1.002)	.31
	MR-Egger	35	1.004 (95% CI: 0.998–1.011)	.20
Hyperkinetic disorders	IVW	42	2.019 (95% CI: 1.263–3.229)	.003
	Weighted median	42	2.565 (95% CI: 1.329–4.950)	.005
	MR-Egger	42	1.647 (95% CI: 0.132–20.620)	.70
Anorexia nervosa	IVW	44	1.19 (95% CI: 1.01–1.40)	.04
	Weighted median	44	1.21 (95% CI: 0.98–1.50)	.08
	MR-Egger	44	1.00 (95% CI: 0.38–2.63)	.99
Autism spectrum disorder	IVW	39	1.10 (95% CI: 1.01–1.20)	.03
	Weighted median	39	1.07 (95% CI: 0.95–1.20)	.26
	MR-Egger	39	0.86 (95% CI: 0.53–1.39)	.53
Psychoactive substance abuse	IVW	44	0.998 (95% CI: 0.997–1.000)	.04
	Weighted median	44	0.998 (95% CI: 0.996–1.000)	.09
	MR-Egger	44	0.995 (95% CI: 0.985–1.004)	.28

Genetic instruments selected from bipolar disorders GWASs, selection threshold *P* less than 5 × 10^−8^, pruned at linkage disequilibrium *R*^2^ < 0.001 (10,000 kb pair window).

CI = confidence interval, GWAS = genome-wide association studies, IVW = inverse-variance weighted, MR = Mendelian randomization, *N* SNV = number of single-nucleotide variants, OR = odds ratio.

However, not all examined traits show significant causal associations with BD. No statistically significant associations are observed for bodily pain (OR = 1.00, 95% CI: 0.999–1.001, *P* = .92), tinnitus (OR = 1.00, 95% CI: 0.99–1.01, *P* = .98), motor disorders (OR = 1.05, 95% CI: 0.94–1.17, *P* = .40), or olfactory dysfunction (OR = 0.83, 95% CI: 0.57–1.23, *P* = .36). The result for multiple sclerosis (OR = 1.03, 95% CI: 2.4E−05 to 4.3E+04, *P* = 1.00) appears nominally positive, but the extensive confidence interval and lack of statistical significance prevent meaningful interpretation.

It is noteworthy that BD may have a slight protective effect on certain behavioral disorders, such as substance use disorders (OR = 0.998, 95% CI: 0.997–1.000, *P* = .04). However, the statistical significance of this effect is weak and requires further verification. Detailed MR scatter plots of BD’s influence on the risk of sensory, motor, and behavioral disorder-related diseases can be found in Supplementary File 2 (Supplemental Digital Content, https://links.lww.com/MD/P738).

### 3.2. MR results of sensory, motor, and behavioral disorders on the risk of BD

**Figure 3.**
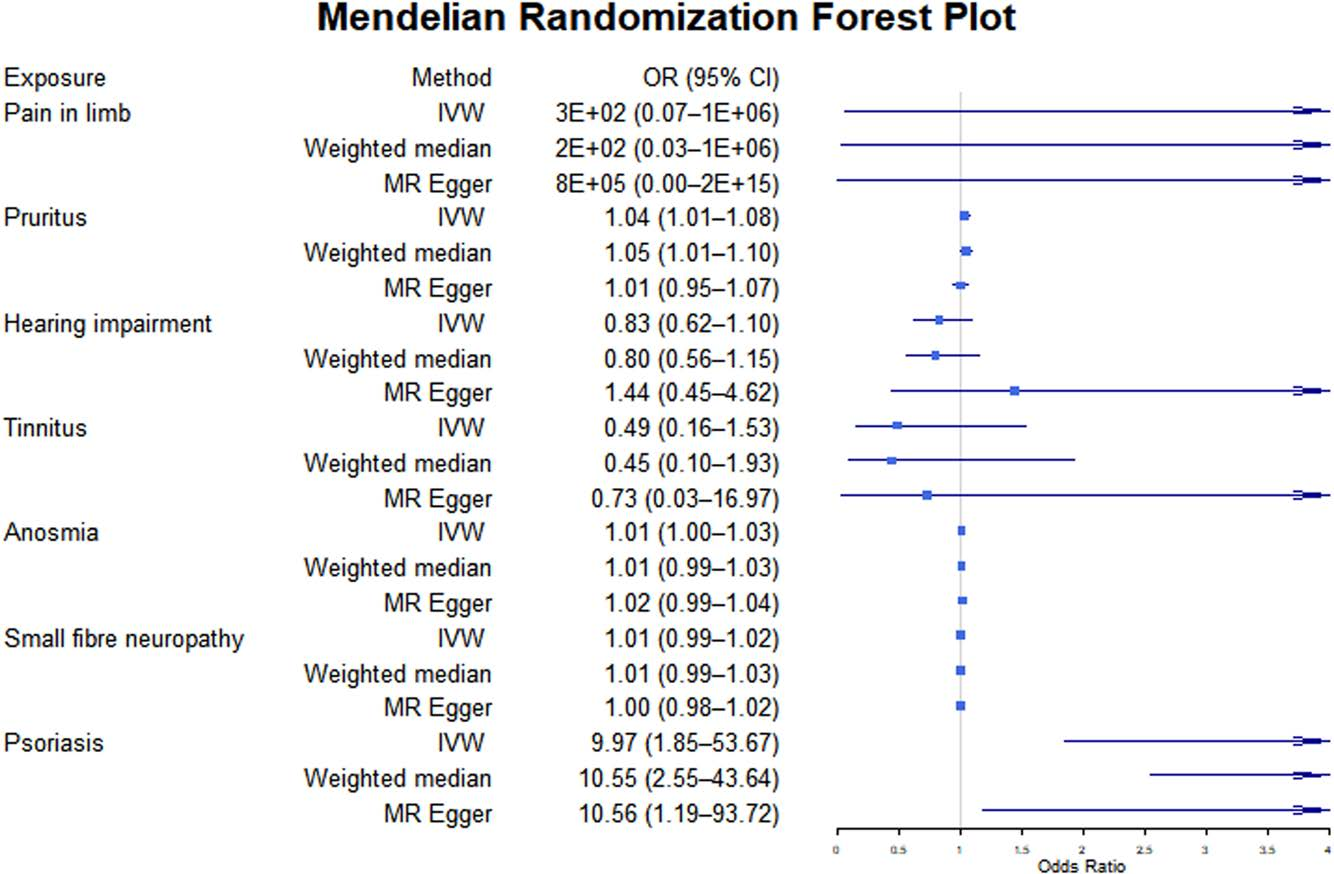
The putative causal effect of sensory disorders on bipolar disorders. The results from Mendelian randomization analysis using the IVW approach, the weighted median approach, or the MR-Egger approach (when horizontal pleiotropy exists). Circles and horizontal bars represent the odds ratios and confidence intervals of the factor with the risk of pain, respectively. CI = confidence interval, IVW = inverse-variance weighted approach, MR = Mendelian randomization, OR = odds ratio.

**Figure 4.**
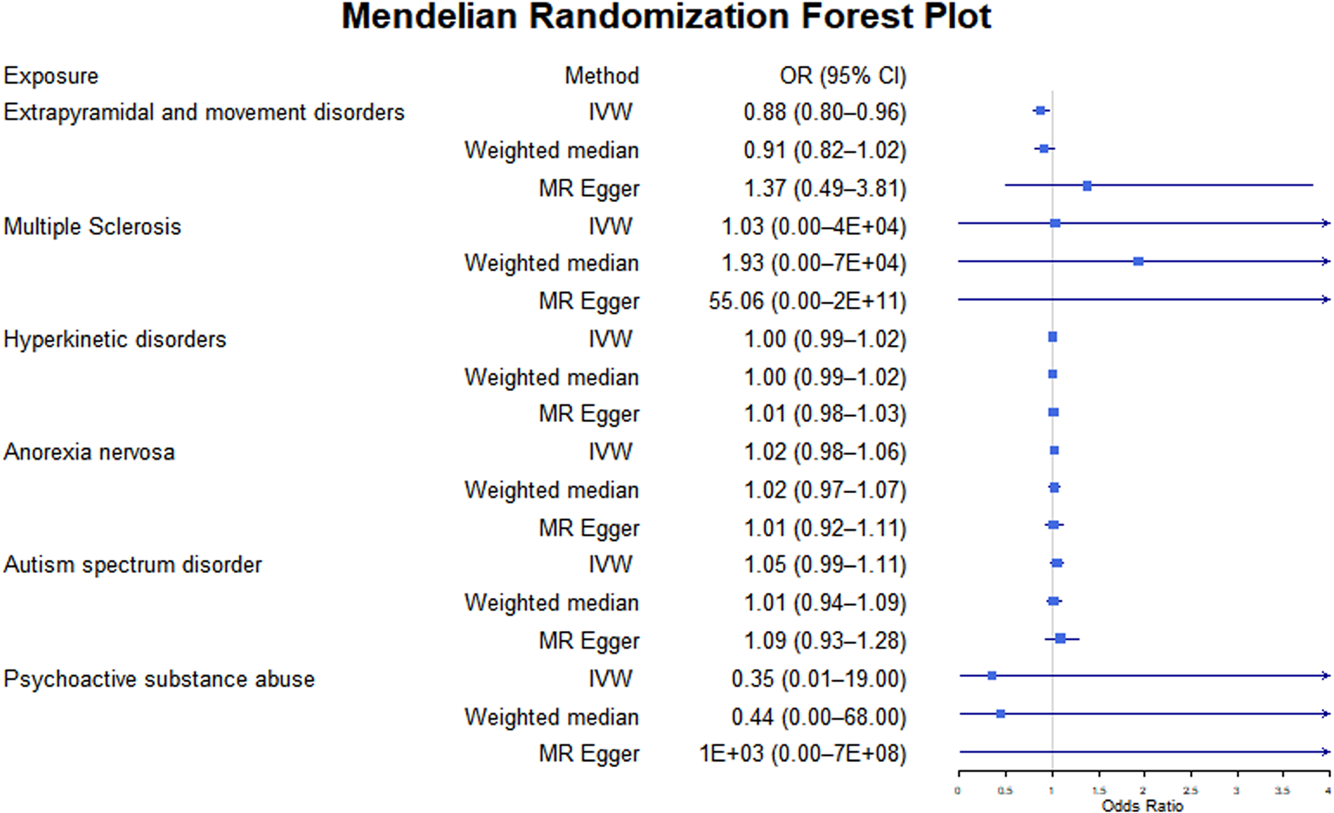
The putative causal effect of motor and behavioral disorders on bipolar disorders. The results from Mendelian randomization analysis using the IVW approach, the weighted median approach, or the MR-Egger approach (when horizontal pleiotropy exists). Circles and horizontal bars represent the odds ratios and confidence intervals of the factor with the risk of pain, respectively. CI = confidence interval, IVW = inverse-variance weighted approach, MR = Mendelian randomization, OR = odds ratio.

As shown in Tables 4 and 5 and Figures 3 and 4, the susceptibility to several sensory, motor, and behavioral disorders is significantly associated with the risk of BD. Pruritus (OR = 1.04, 95% CI: 1.01–1.08, *P* = .007) and psoriasis (OR = 9.97, 95% CI: 1.85–53.67, *P* = .007) are identified as significant risk factors for BD. These associations are further supported by the weighted median and MR-Egger methods, with pruritus (Weighted median OR = 1.05, 95% CI: 1.01–1.10, *P* = .024) and psoriasis (Weighted median OR = 10.55, 95% CI: 2.55–43.64, *P* = .002) showing consistent effects.

**Table 4:** The univariate MR results of sensory disorders on the risk of bipolar disorders.

Exposure	Method	Bipolar disorders		
		*N* SNV	OR (95% CI)	*P* value
Pain in the limb*	IVW	10	280.69 (95% CI: 0.07–1.06E + 06)	.18
	Weighted median	10	195.51 (95% CI: 0.03–1.16E + 06)	.23
	MR-Egger	10	757,706.3 (95% CI: 0.0002–2.44E + 15)	.29
Pruritus*	IVW	7	1.04 (95% CI: 1.01–1.08)	.007
	Weighted median	7	1.05 (95% CI: 1.01–1.10)	.024
	MR-Egger	7	1.01 (95% CI: 0.95–1.07)	.749
Hearing impairment	IVW	27	0.83 (95% CI: 0.62–1.10)	.92
	Weighted median	27	0.80 (95% CI: 0.56–1.15)	.23
	MR-Egger	27	1.44 (95% CI: 0.45–4.62)	.55
Tinnitus*	IVW	15	0.49 (95% CI: 0.16–1.53)	.22
	Weighted median	15	0.45 (95% CI: 0.10–1.93)	.28
	MR-Egger	15	0.73 (95% CI: 0.03–16.97)	.85
Anosmia*	IVW	11	1.013 (95% CI: 1.000–1.026)	.055
	Weighted median	11	1.011 (95% CI: 0.993–1.028)	.238
	MR-Egger	11	1.016 (95% CI: 0.992–1.040)	.237
Small fiber neuropathy*	IVW	6	1.01 (95% CI: 0.99–1.02)	.32
	Weighted median	6	1.01 (95% CI: 0.99–1.03)	.30
	MR-Egger	6	1.00 (95% CI: 0.98–1.02)	.86
Psoriasis	IVW	22	9.97 (95% CI: 1.85–53.67)	.007
	Weighted median	22	10.55 (95% CI: 2.55–43.64)	.002
	MR-Egger	22	10.56 (95% CI: 1.19–93.72)	.05

Genetic instruments selected from bipolar disorders GWASs, selection threshold *P* less than 5 × 10^−8^, pruned at linkage disequilibrium *R*^2^ < 0.001 (10,000 kb pair window).

CI = confidence interval, GWAS = genome-wide association studies, IVW = inverse-variance weighted, MR = Mendelian randomization, *N* SNV = number of single-nucleotide variants, OR = odds ratio.

* If the number of SNPs available for analysis is <3, the selection threshold *P* will be adjusted to 5 × 10^−6^.

**Table 5:** The univariate MR results of bipolar disorders on the risk of motor and behavioral disorders.

Exposure	Method	Bipolar disorders		
		*N* SNV	OR (95%CI)	*P* value
Extrapyramidal and movement disorders*	IVW	6	0.876 (95% CI: 0.798–0.961)	.005
	Weighted median	6	0.913 (95% CI: 0.818–1.019)	.106
	MR-Egger	6	1.374 (95% CI: 0.495–3.809)	.575
Multiple sclerosis	IVW	5	1.03 (95% CI: 2.4E−05 to 4.3E+04)	1.00
	Weighted median	5	1.93 (95% CI: 5.6E−05 to 6.7E+04)	.90
	MR-Egger	5	55.06 (95% CI: 1.3E−08 to 2.4E+11)	.75
Hyperkinetic disorders*	IVW	8	1.00 (95% CI: 0.99–1.02)	.76
	Weighted median	8	1.00 (95% CI: 0.99–1.02)	.72
	MR-Egger	8	1.01 (95% CI: 0.98–1.03)	.69
Anorexia nervosa*	IVW	12	1.02 (95% CI: 0.98–1.06)	.30
	Weighted median	12	1.02 (95% CI: 0.97–1.07)	.50
	MR-Egger	12	1.01 (95% CI: 0.92–1.11)	.83
Autism spectrum disorder*	IVW	27	1.05 (95% CI: 0.99–1.11)	.09
	Weighted median	27	1.01 (95% CI: 0.94–1.09)	.85
	MR-Egger	27	1.09 (95% CI: 0.93–1.28)	.30
Psychoactive substance abuse*	IVW	10	0.35 (95% CI: 6.4E−03 to 1.9E+01)	.61
	Weighted median	10	0.44 (95% CI: 2.9E−03 to 6.8E+01)	.75
	MR-Egger	10	1.04E+03 (95% CI: 1.7E−03 to 6.6E+08)	.34

Genetic instruments selected from bipolar disorders GWASs, selection threshold *P* less than 5 × 10^−8^, pruned at linkage disequilibrium *R*^*2*^ <0.001 (10,000 kb pair window).

CI = confidence interval, GWAS = genome-wide association studies, IVW = inverse-variance weighted, MR = Mendelian randomization, *N* SNV = number of single-nucleotide variants, OR = odds ratio.

* If the number of SNPs available for analysis is <3, the selection threshold *P* will be adjusted to 5 × 10^−6^.

Motor disorders are associated with a decreased risk of BD (OR = 0.876, 95% CI: 0.798–0.961, *P* = .005). However, this association does not reach statistical significance in the weighted median method (OR = 0.913, 95% CI: 0.818–1.019, *P* = .106), though a similar protective trend is observed.

In contrast, most other sensory, motor, and behavioral disorders do not exhibit significant associations with the risk of BD. For example, bodily pain (IVW OR = 280.69, 95% CI: 0.07–1.06E + 06, *P* = .18), tinnitus (IVW OR = 0.49, 95% CI: 0.16–1.53, *P* = .22), and ASD (IVW OR = 1.05, 95% CI: 0.99–1.11, *P* = .09) fail to reach significance. The estimate for multiple sclerosis (IVW OR = 1.03, 95% CI: 2.4E-05–4.3E + 04, *P* = 1.00) has an extensive confidence interval, indicating a lack of statistical support.

It is worth noting that some disorders approached statistical significance. For example, the IVW method for olfactory dysfunction (OR = 1.013, 95% CI: 1.000–1.026, *P* = .055) suggests a potential positive association, though other methods did not support this finding (Weighted median OR = 1.011, *P* = .238; MR-Egger OR = 1.016, *P* = .237). Detailed MR scatter plots of the impact of sensory, motor, and behavioral disorder-related diseases on BD risk can be found in Supplementary File 3 (Supplemental Digital Content, https://links.lww.com/MD/P738).

### 3.3. Sensitivity analyses

Supplementary Files 4 and 5 (Supplemental Digital Content, https://links.lww.com/MD/P738) provide the heterogeneity between genetic instruments and horizontal pleiotropy. Leave-one-out analysis indicates that the causal estimates are not driven by any single SNP (Supplementary File 6, Supplemental Digital Content, https://links.lww.com/MD/P738). Furthermore, the *F*-statistic values for the instruments range from 20.76 to 3089.50, suggesting that no weak instrument bias exists in this MR study.

## 4. Discussion

BD is a complex psychiatric condition characterized by recurrent episodes of mania and depression, disrupting emotion regulation, behavior, and social functioning. The comorbidity between BD and sensory, motor, and behavioral disorders has attracted increasing clinical and research interest, as these conditions add to patient burden and complicate clinical management. With the global prevalence of these disorders on the rise, their impact on public health systems and healthcare resources continues to escalate. Uncovering the causal links and underlying biological mechanisms between BD and these comorbid conditions has therefore become a pressing challenge in psychiatry.

However, the causal relationships remain poorly defined, mainly due to the practical constraints of RCTs, including high cost, ethical concerns, and extended timelines. MR offers a robust alternative for causal inference, leveraging genetic variants as IVs to explore exposure–outcome relationships using observational data. Compared to RCTs, MR minimizes confounding and reverse causality, making it a valuable method in genetic epidemiology for investigating complex disease mechanisms. MR is increasingly recognized as a promising tool in advancing public health and precision medicine.

In this study, we analyzed BD and 13 sensory, motor, and behavioral disorders as exposures and outcomes, using 2 primary MR methods: IVW and MR-Egger regression. Both approaches rely on variance weighting, though their underlying assumptions differ. The IVW method, which assumes no horizontal pleiotropy, excludes an intercept term from the regression model and thus offers greater statistical power under this assumption. Accordingly, IVW was selected as the primary analytic approach. To account for potential bias due to horizontal pleiotropy, we additionally employed MR-Egger, which incorporates an intercept term to detect and adjust for directional pleiotropy, thereby providing complementary and more robust causal inference when pleiotropy is present. The analysis reveals a significant bidirectional association between pruritus (itching) and genetic susceptibility to BD. We also identified BD as a causal risk factor for small fiber neuropathy, hyperkinetic disorders, anorexia nervosa, and ASD. Moreover, BD appears to confer a mild protective effect against substance use disorders. In the reverse direction, psoriasis is a notable genetic determinant of BD susceptibility. Additionally, motor disorders are associated with a reduced risk of BD.

Our findings on the association between BD and psoriasis,^[41]^ hyperkinetic disorders,^[25]^ anorexia nervosa,^[26]^ and ASD^[27,28]^ are consistent with previous observational studies. However, our study extends this evidence by providing genetic support for a causal relationship. Prior research has shown that individuals with psychiatric disorders characterized by emotional dysregulation – such as BD, major depression, anxiety, and panic disorder – exhibit structural and functional alterations in the somatosensory cortex, including changes in gray matter volume, cortical thickness, functional connectivity with other brain regions, and metabolic activity.^[42]^ Building on this neurobiological evidence, our results reveal a significant genetic causal link between BD and 3 skin-related sensory disorders: pruritus (itching), small fiber neuropathy, and psoriasis, suggesting potential neurocutaneous interactions. Notably, itching and small fiber neuropathy are genetically linked to BD for the first time, highlighting novel targets for understanding shared sensory and affective pathophysiology.

These findings suggest that pruritus may be an early clinical indicator of BD. Clinically, greater attention should be given to sensory comorbidities, such as itching, in BD patients, warranting targeted management strategies to improve quality of life. This may represent a valuable clinical entry point for integrated care. From a disease mechanism perspective, the somatosensory cortex emerges as a potential therapeutic target for BD. Future research should investigate the pathophysiological underpinnings of these relationships, including inflammatory signaling pathways, neurotransmitter dysregulation, and the role of the neuroimmune axis in mediating BD and its sensory comorbidities. Such studies may pave the way for more precise and personalized treatment approaches.

Additionally, our results indicate that BD is genetically associated with increased susceptibility to hyperkinetic disorders, anorexia nervosa, and ASD. These findings underscore the need for early screening and intervention in BD patients exhibiting behavioral abnormalities, particularly those with a family history of related disorders. Greater emphasis on recognizing and diagnosing comorbid behavioral disorders will help inform more comprehensive and effective treatment strategies, ultimately improving long-term prognosis. We propose that these psychiatric and behavioral disorders may share standard genetic and biological mechanisms. Previous studies have reported differences in copy number variation burden between BD and ASD, yet both disorders involve chromatin-related molecular mechanisms, despite divergent neurodevelopmental profiles and regulatory copy number variation patterns.^[43]^ Further investigation into shared mechanisms – such as neurodevelopmental dysregulation, altered synaptic plasticity, and neurotransmitter pathway disturbances – will offer novel insights for identifying therapeutic targets in BD patients with behavioral comorbidities.

This study presents several notable strengths. First, it employs a comprehensive bidirectional MR framework to assess the causal relationships between BD and 13 sensory, motor, and behavioral disorders. This approach reveals the potential downstream effects of BD and evaluates reverse causality, providing novel insight into their bidirectional interactions. Unlike traditional observational studies, MR substantially mitigates confounding and reverse causality, thereby enhancing the validity of causal inference. Second, the study leverages large-scale GWAS datasets and applies stringent criteria for selecting SNPs, ensuring that the IVs correlate strongly with the exposures. The use of multiple MR methods, including IVW and MR-Egger, reinforces the robustness and consistency of the findings. Moreover, this study identifies previously unrecognized causal links between BD and specific sensory comorbidities, such as pruritus and small fiber neuropathy, addressing a critical gap in the literature. The findings also highlight a significant genetic association between BD and cutaneous sensory disorders (e.g., pruritus, psoriasis), suggesting that itching may serve as a prodromal or early observational marker for BD. Finally, our results demonstrate that BD increases genetic susceptibility to attention-deficit/hyperactivity disorder, anorexia nervosa, and ASD. These associations imply shared molecular mechanisms, including neurodevelopmental disruptions, impaired synaptic plasticity, and altered neurotransmitter signaling pathways. Collectively, these findings advance our understanding of the pathophysiological underpinnings of BD and its comorbidities and offer a compelling rationale for early clinical screening, integrated treatment strategies, and precision medicine–based interventions in affected populations.

However, this study has several limitations. First, the genetic data were primarily derived from individuals of European ancestry, which may limit generalizability across populations. Further validation in non-European cohorts is warranted to assess the consistency of genetic associations. Because MR estimates reflect lifetime genetic liability, they may not capture the effects of short-term or dynamic exposures. In addition, some GWAS datasets were based on self-reported data, potentially introducing subjective bias. For example, participants may have misclassified sensory conditions due to limited medical knowledge, affecting exposure definition reliability. Second, the study did not differentiate certain disorders’ clinical subtypes or severity levels. For instance, small fiber neuropathy encompasses multiple subtypes that may differentially influence BD, and the severity spectrum of behavioral disorders (e.g., hyperkinetic disorders, ASD) was not incorporated, limiting granularity in causal interpretation. Moreover, some participants may have had multiple co-occurring disorders, and the interactions between comorbidities were not fully adjusted for, which may have introduced residual confounding. The instrument strength for specific exposures (e.g., multiple sclerosis) was relatively weak, potentially reducing causal inference’s statistical power and robustness. Finally, no formal correction for multiple testing was applied, increasing the risk of type I errors and limiting the interpretability of marginal associations.

In conclusion, future research is encouraged to improve and expand on multiple levels. On one hand, incorporating more diverse population samples and refining the classification of disorder subtypes and severity levels can enhance the generalizability and clinical relevance of the findings. In addition, strengthening clinical validation and more thoroughly controlling for confounding variables improves the robustness and credibility of causal inferences. On the other hand, from a methodological perspective, integrating higher-quality data and more advanced statistical techniques is recommended to replicate this study’s main findings and further elucidate the causal mechanisms linking BD with sensory, motor, and behavioral disorders. These efforts are expected to provide a stronger theoretical foundation and practical support for early screening, clinical diagnosis, and personalized treatment strategies for BD-related comorbidities.

## 5. Conclusions

This study employs a bidirectional MR approach to investigate the complex causal relationships between BD and sensory, motor, and behavioral disorders. The findings reveal a significant bidirectional association between pruritus (itching) and genetic susceptibility to BD. BD is also identified as a causal factor for increased genetic susceptibility to small fiber neuropathy, attention-deficit/hyperactivity disorder (hyperkinetic disorders), anorexia nervosa, and ASD. In contrast, BD may exert a modest protective effect on the risk of substance use disorders. Additionally, psoriasis is implicated as a unidirectional genetic risk factor for BD, whereas motor disorders appear to have a potentially mitigating effect. The results suggest that skin sensory abnormalities may serve as early observable indicators of BD, highlighting the clinical importance of recognizing and managing sensory comorbidities in affected individuals. Furthermore, elucidating shared genetic pathways between BD and co-occurring behavioral disorders offers a promising direction for future research. This study strengthens the causal evidence linking BD with sensory, motor, and behavioral disorders and provides a theoretical basis for integrated clinical management and precision intervention strategies. These insights are particularly valuable for early detection, targeted treatment, and individualized care, potentially reducing disease burden and enhancing patient quality of life.

## Acknowledgments

We thank all participants and investigators of the included GWAS studies for contributing to the data.

## Author contributions

**Conceptualization:** Yuyan Chen, Bangqi Wu.

**Data curation:** Yuyan Chen, Yupei Cheng, Jingjie Huang.

**Formal analysis:** Yuyan Chen.

**Funding acquisition:** Bangqi Wu.

**Methodology:** Yan Zhang.

**Project administration:** Yan Zhang.

**Validation:** Yupei Cheng, Jingjie Huang.

**Visualization:** Yuyan Chen, Chaoran Wang, Jing Bai, Yuxing Zhang.

**Writing – original draft:** Yuyan Chen.

**Writing – review & editing:** Bangqi Wu.

## Supplementary Material


