# Supplementary material for: Exploring the causal relationship between bipolar disorders and sensory, motor, and behavioral disorders: A bidirectional Mendelian randomization analysis
Source: Medicine (Baltimore). 2025 Aug 22;104(34):e44056. doi: 10.1097/MD.0000000000044056 (PMC12385034; doi:10.1097/MD.0000000000044056)
Supplement: Supplementary file 1 [file medi-104-e44056-s001.pdf]

Supplementary file 1

Descriptions of instrument variables in the MR analysis

Characteristics of the 52 bipolar disorders associated instruments

SNP	A1	A2	*Beta*	*SE*	*P*-value	*F*-statistic
rs2126180	A	G	0.0566021	0.0094	1.62E-09	36.25846225
rs10737496	T	C	-0.0542041	0.0094	7.17E-09	33.25129535
rs13417268	G	C	-0.0618004	0.011	2.05E-08	31.56437554
rs4619651	A	G	-0.0660967	0.0101	4.78E-11	42.82691649
rs17183814	A	G	-0.102899	0.0185	2.68E-08	30.93704661
rs2011302	A	T	0.0529051	0.0097	4.25E-08	29.74757791
rs9834970	C	T	0.0830012	0.0093	6.63E-19	79.65312986
rs2336147	C	T	-0.067696	0.0093	3.61E-13	52.98587601
rs6806239	G	T	-0.0660967	0.0119	2.64E-08	30.85074324
rs696366	A	C	-0.0515957	0.0094	4.46E-08	30.12806992
rs112481526	G	A	0.0630996	0.0105	1.86E-09	36.11391855
rs7707252	G	A	0.0571949	0.0104	3.64E-08	30.24460601
rs6887473	A	G	-0.0603046	0.0105	8.81E-09	32.98544019
rs10043984	T	C	0.0593042	0.0108	3.71E-08	30.15250461
rs10866641	C	T	-0.0625991	0.0094	2.79E-11	44.34865687
rs28565152	A	G	0.0671018	0.0112	1.96E-09	35.89486259
rs1487445	T	C	0.0741957	0.0093	1.48E-15	63.64899871
rs10455979	G	C	0.0555973	0.0095	4.22E-09	34.24996972
rs34505465	T	G	-0.1364	0.0175	5.75E-15	60.7508898
rs41315395	A	C	0.0719042	0.0127	1.48E-08	32.05539077
rs10255167	A	G	0.0663962	0.0118	1.60E-08	31.66084009
rs113779084	A	G	0.0754997	0.0102	1.42E-13	54.78858804
rs11764361	G	A	-0.0614995	0.0104	3.47E-09	34.96845877
rs12668848	A	G	-0.057004	0.0095	1.90E-09	36.00505281
rs6954854	A	G	-0.0582972	0.0094	5.94E-10	38.46269271
rs6946056	C	A	0.0532004	0.0097	3.66E-08	30.08058837
rs6992333	G	A	0.060196	0.01	1.62E-09	36.23558416
rs62489493	G	C	0.089695	0.0135	2.64E-11	44.1437203
rs2953928	A	G	0.116096	0.02	6.25E-09	33.69570304
rs62581014	T	C	0.0652001	0.0117	2.77E-08	31.05451852
rs1998820	A	T	-0.0836976	0.0152	4.10E-08	30.32067281
rs2273738	T	C	0.0916987	0.0136	1.63E-11	45.46200033
rs10994415	C	T	0.118097	0.0174	1.14E-11	46.0658654
rs7108878	G	T	0.081004	0.0147	3.61E-08	30.36534785
rs10791849	A	T	-0.0693955	0.0121	9.89E-09	32.8921209
rs174592	G	A	0.072001	0.0097	9.92E-14	55.09771496
rs12575685	A	G	0.0652001	0.0101	1.24E-10	41.67290501
rs11062170	C	G	0.0778967	0.0098	1.87E-15	63.18092327
rs35306827	A	G	-0.0659001	0.0112	3.56E-09	34.6207205
rs2693698	G	A	0.053	0.0094	1.96E-08	31.7904029
rs748455	C	T	-0.0674998	0.0103	5.01E-11	42.94677161

rs35958438	A	G	-0.0642041	0.0117	3.83E-08	30.11298456
rs4447398	C	A	-0.0821973	0.0138	2.61E-09	35.47782045
rs7201930	C	T	0.0586042	0.0104	1.89E-08	31.75344173
rs28455634	A	G	-0.0627964	0.0099	2.63E-10	40.23454599
rs4790841	T	C	0.0729041	0.0132	3.14E-08	30.50394741
rs61554907	T	G	0.0868005	0.0154	1.64E-08	31.76896104
rs228768	T	G	-0.064401	0.0102	2.83E-10	39.86436756
rs6104027	G	A	-0.0603046	0.0095	1.93E-10	40.29523303
rs237460	T	C	0.0553013	0.0094	4.25E-09	34.61106589
rs13044225	G	A	0.0546991	0.0095	8.50E-09	33.15226084
rs5758064	C	T	-0.0524027	0.0093	2.01E-08	31.74983197

SNP: single nucleotide polymorphism; A1: effect allele; A2: other allele.

Characteristics of the 14 pain in limb associated instruments

**SNP**	**A1**	**A2**	**EAF**	***Beta***	***SE***	***P*-value**	***F*-statistic**
rs6025	C	T	0.977059	-0.00411564	0.000789917	1.89E-07	27.14637462
rs11900952	A	G	0.361862	0.00116144	0.000245883	2.32E-06	22.31189983
rs79368524	A	T	0.00822812	0.00624806	0.00135844	4.24E-06	21.15482615
rs9307063	T	G	0.893967	-0.00181059	0.000384288	2.46E-06	22.19865126
rs28594099	T	G	0.048514	0.00262766	0.000570427	4.10E-06	21.21964883
rs114850142	T	C	0.0441051	0.00275621	0.000574068	1.58E-06	23.0514331
rs7894505	A	G	0.027596	0.00334086	0.000720368	3.52E-06	21.50838138
rs75470588	G	A	0.108302	0.00184064	0.000382486	1.49E-06	23.15829727
rs191707225	G	A	0.00866948	0.00635447	0.00137725	3.95E-06	21.28791391
rs111301272	T	C	0.42365	0.00124003	0.000259367	1.75E-06	22.85782728
rs78971855	C	G	0.0377405	0.00308061	0.000620436	6.86E-07	24.65354992
rs62042161	T	A	0.0194897	0.00406576	0.000875487	3.42E-06	21.56671873
rs117296148	G	C	0.0427471	-0.00299343	0.000589503	3.82E-07	25.78494293
rs139945846	C	G	0.013451	0.00548238	0.00111678	9.15E-07	24.09922151

SNP: single nucleotide polymorphism; A1: effect allele; A2: other allele; EAF: effect allele frequency.

Characteristics of the 7 pruritus associated instruments

SNP	A1	A2	EAF	*Beta*	*SE*	*P*-value	*F*-statistic
rs115770010	A	T	0.02208	0.758	0.141	7.69E-08	28.90015593
rs10001107	G	A	0.07361	0.3847	0.0758	3.87E-07	25.75763361
rs72678441	G	A	0.08891	0.3495	0.0695	4.86E-07	25.2885979
rs10812124	C	A	0.4553	-0.1826	0.0388	2.54E-06	22.14818259
rs117406698	A	G	0.00649	1.3363	0.2822	2.19E-06	22.42301038
rs4366152	C	T	0.7078	0.2037	0.0425	1.64E-06	22.97228512
rs77495968	A	G	0.007691	1.1275	0.2457	4.45E-06	21.05827477

SNP: single nucleotide polymorphism; A1: effect allele; A2: other allele; EAF: effect allele frequency.

Characteristics of the 30 hearing impairment associated instruments

**SNP**	**A1**	**A2**	***Beta***	***SE***	***P*-value**	***F*-statistic**
rs7525101	T	C	-0.0144609	0.00226128	1.61E-10	40.89610645
rs741475	T	C	0.0131215	0.0022723	7.72E-09	33.34537705
rs2941580	A	G	0.0127288	0.00224942	1.53E-08	32.02092114
rs6443802	A	C	0.0166399	0.00238352	2.93E-12	48.73756452
rs3915060	T	C	0.0146989	0.00252502	5.84E-09	33.88753864
rs13148153	T	C	-0.0190535	0.00329421	7.30E-09	33.45391632
rs34929759	C	T	-0.022056	0.00224867	1.04E-22	96.20597701
rs13204736	A	G	-0.0171985	0.00235443	2.78E-13	53.35923268
rs10948071	T	C	-0.0203386	0.00230183	9.94E-19	78.07205632
rs9493627	A	G	-0.0148024	0.00240617	7.66E-10	37.8452742
rs6902016	T	C	-0.0159999	0.00224565	1.04E-12	50.7633644
rs6968827	G	A	-0.0153899	0.00266256	7.47E-09	33.40971641
rs4732339	A	G	-0.0126976	0.00227783	2.48E-08	31.0742508
rs74544416	G	A	-0.0245055	0.00438286	2.25E-08	31.26165309
rs13277721	A	G	-0.0140294	0.0022454	4.16E-10	39.03828758
rs835259	G	T	0.012364	0.00224758	3.78E-08	30.26130655
rs117583072	A	G	-0.0537772	0.00950561	1.54E-08	32.00636758
rs10901863	T	C	-0.0211667	0.00253537	6.91E-17	69.69852937
rs118176061	C	T	-0.0318873	0.00580568	3.96E-08	30.16680433
rs55635402	G	A	0.0190734	0.0028278	1.53E-11	45.49449548
rs67307131	C	T	-0.0169159	0.00235864	7.40E-13	51.43596586
rs1126809	A	G	-0.0190899	0.00243852	4.94E-15	61.28506669
rs1566129	C	T	0.0148517	0.00227878	7.15E-11	42.47638394
rs62033400	G	A	0.0131836	0.00229705	9.50E-09	32.94026876
rs78417468	A	G	0.0152584	0.00268558	1.33E-08	32.28061525
rs12938775	A	G	0.0129444	0.00224484	8.10E-09	33.25010836
rs4611552	C	T	-0.0153578	0.00273309	1.92E-08	31.57549364
rs11881070	T	C	0.0145896	0.00247684	3.85E-09	34.6969152
rs36062310	A	G	-0.0444537	0.00551869	7.94E-16	64.88492846
rs132929	A	G	-0.0148104	0.00227857	8.04E-11	42.24825989

SNP: single nucleotide polymorphism; A1: effect allele; A2: other allele.

Characteristics of the 21 tinnitus associated instruments

SNP	A1	A2	EAF	*Beta*	*SE*	*P*-value	*F*-statistic
rs7600596	G	A	0.243972	0.00654572	0.00123542	1.17E-07	28.072791
rs72817942	T	C	0.0243728	-0.0156016	0.00341141	4.81E-06	20.9156055
rs16851797	T	G	0.0523178	0.0116683	0.00239203	1.07E-06	23.79479289
rs4705746	A	C	0.739526	0.00664059	0.00120743	3.81E-08	30.24749473
rs138988422	T	C	0.0143269	-0.0214837	0.00453123	2.13E-06	22.47946362
rs76488086	A	C	0.0253011	0.0157838	0.00344543	4.63E-06	20.98631932
rs77101377	G	A	0.0576529	0.0109188	0.00225388	1.27E-06	23.46865726
rs10227990	T	C	0.777003	-0.00606902	0.0012813	2.18E-06	22.43548617
rs28810390	G	A	0.0987295	0.00822	0.00176931	3.39E-06	21.58419077
rs72621174	C	T	0.0171279	-0.0184654	0.00402575	4.51E-06	21.0389391
rs1119017	T	C	0.209789	-0.00601171	0.00130299	3.96E-06	21.28697154
rs185376729	G	C	0.0041133	0.0417908	0.00905251	3.91E-06	21.31195759
rs11013071	C	T	0.104447	0.00799805	0.00174889	4.81E-06	20.91429553
rs67329178	A	G	0.01324	0.0224077	0.0048196	3.33E-06	21.61586261
rs144593498	C	A	0.0085024	0.0292934	0.00628322	3.13E-06	21.73577006
rs2908471	C	T	0.802039	0.00687597	0.0013703	5.23E-07	25.17889292
rs62039092	C	T	0.117271	-0.0083161	0.00165892	5.37E-07	25.12977036
rs117732100	G	A	0.00961598	0.0278155	0.00601843	3.81E-06	21.36029837
rs35133835	T	G	0.114084	0.00775411	0.0016615	3.06E-06	21.78026824
rs77816404	G	T	0.0134594	0.0214887	0.00459352	2.90E-06	21.88411873
rs73102406	C	T	0.198642	-0.00609576	0.00133038	4.61E-06	20.99444048

SNP: single nucleotide polymorphism; A1: effect allele; A2: other allele; EAF: effect allele frequency.

Characteristics of the 13 anosmia associated instruments

SNP	A1	A2	EAF	*Beta*	*SE*	*P*-value	*F*-statistic
rs149459000	T	C	0.002576	5.3941	1.1464	2.54E-06	22.13938905
rs13027132	C	A	0.2172	-0.4176	0.0913	4.79E-06	20.92085478
rs114566775	T	C	0.01645	1.6425	0.3387	1.24E-06	23.51691574
rs1107655	C	A	0.8881	-0.585	0.1224	1.74E-06	22.84277682
rs77064330	T	C	0.02158	1.4158	0.2854	7.03E-07	24.60910835
rs7702774	T	G	0.5135	-0.3426	0.0748	4.72E-06	20.9783594
rs16872237	T	C	0.1054	0.6054	0.1263	1.63E-06	22.97619625
rs41266341	C	A	0.001254	8.911	1.9349	4.12E-06	21.20976409
rs146042559	T	C	0.008129	2.6335	0.5388	1.02E-06	23.88974264
rs751709	T	G	0.02479	1.1989	0.26	3.98E-06	21.26273979
rs11048761	A	G	0.1829	0.4787	0.0985	1.16E-06	23.61861321
rs113377917	A	G	0.1204	0.5959	0.1192	5.79E-07	24.99161144
rs7231107	C	T	0.01836	1.5088	0.3125	1.38E-06	23.31112899

SNP: single nucleotide polymorphism; A1: effect allele; A2: other allele; EAF: effect allele frequency.

Characteristics of the 7 small fibre neuropathy associated instruments

**SNP**	**A1**	**A2**	**EAF**	***Beta***	***SE***	***P*-value**	***F*-statistic**
rs115787256	T	C	0.006975	3.5059	0.7057	6.76E-07	24.6807762
rs141737598	T	C	0.0308	1.3689	0.2981	4.38E-06	21.08722806
rs145703410	T	C	0.0218	1.9801	0.387	3.11E-07	26.17895566
rs142215775	G	T	0.01393	2.3324	0.4847	1.49E-06	23.15581259
rs151067744	A	G	0.09637	0.7613	0.1645	3.68E-06	21.4180464
rs72891041	T	G	0.08447	0.8602	0.1771	1.19E-06	23.59183673
rs4310980	A	G	0.3225	0.4693	0.0984	1.86E-06	22.74630779

SNP: single nucleotide polymorphism; A1: effect allele; A2: other allele; EAF: effect allele frequency.

Characteristics of the 21 psoriasis associated instruments

**SNP**	**A1**	**A2**	**EAF**	***Beta***	***SE***	***P*-value**	***F*-statistic**
rs11581607	A	G	0.066756	-0.00258652	0.000442884	5.20E-09	34.10764232
rs4112787	T	C	0.658527	0.00164731	0.000233284	1.60E-12	49.86327082
rs842636	A	G	0.435424	-0.0013276	0.00022268	2.50E-09	35.54447158
rs2111485	G	A	0.606734	0.00138038	0.000226111	1.00E-09	37.26953164
rs11135059	A	G	0.329065	-0.00251427	0.000235663	1.40E-26	113.8258817
rs848	C	A	0.816194	0.00179998	0.000285251	2.80E-10	39.81814355
rs12188300	T	A	0.093472	0.00512622	0.000379811	1.60E-41	182.1626789
rs12189871	T	C	0.090526	0.0213637	0.000384355	1.00E-200	3089.497665
rs28367705	A	G	0.110929	0.00701345	0.0004472	2.00E-55	245.9573587
rs9277937	C	T	0.096319	0.00285115	0.000375617	3.20E-14	57.61686885
rs33980500	T	C	0.074392	0.00280706	0.000419981	2.30E-11	44.67289573
rs2735003	G	T	0.207994	-0.00259899	0.000271761	1.10E-21	91.46079101
rs582757	T	C	0.726237	-0.00137827	0.000247418	2.50E-08	31.03173247
rs9265203	T	G	0.452948	0.00622853	0.000252624	3.20E-134	607.885671
rs13191494	C	G	0.100222	0.00435168	0.000394465	2.70E-28	121.7017866
rs11795343	C	T	0.401328	-0.00144443	0.000226388	1.80E-10	40.70860256
rs7951925	G	A	0.368627	-0.00127836	0.000229137	2.40E-08	31.12546875
rs8016947	G	T	0.561824	0.00161674	0.000222823	4.00E-13	52.64538821
rs28998802	A	G	0.140482	0.00186972	0.000322335	6.60E-09	33.64637007
rs11085725	T	C	0.292402	-0.00166753	0.000243626	7.70E-12	46.84896948
rs632376	G	A	0.41992	-0.00131604	0.00022408	4.30E-09	34.49308093

SNP: single nucleotide polymorphism; A1: effect allele; A2: other allele; EAF: effect allele frequency.

Characteristics of the 8 extrapyramidal and movement disorders associated instruments

**SNP**	**A1**	**A2**	**EAF**	***Beta***	***SE***	***P*-value**	***F*-statistic**
rs4660144	T	C	0.5914	0.1019	0.0214	1.83E-06	22.67361778
rs7534281	C	T	0.6982	-0.1077	0.0228	2.27E-06	22.31319252
rs9288883	G	A	0.3345	-0.1056	0.0223	2.17E-06	22.42425949
rs145432962	A	G	0.005426	0.7683	0.155	7.13E-07	24.56961041
rs10078642	G	A	0.5622	-0.0966	0.021	4.37E-06	21.16
rs463189	T	C	0.7347	0.1138	0.0238	1.69E-06	22.86286279
rs8044719	G	T	0.8408	0.1325	0.0287	3.97E-06	21.31414731
rs372603764	C	A	0.01823	-0.4051	0.088	4.13E-06	21.19137526

SNP: single nucleotide polymorphism; A1: effect allele; A2: other allele; EAF: effect allele frequency.

Characteristics of the 5 multiple sclerosis associated instruments

**SNP**	**A1**	**A2**	**EAF**	***Beta***	***SE***	***P*-value**	***F*-statistic**
rs67382147	G	T	0.772408	-0.00369994	0.000163096	6.19E-114	514.6390574
rs9378141	C	A	0.284227	-0.00132374	0.000138928	1.60E-21	90.78744985
rs28746956	G	T	0.237273	-0.00136306	0.000147456	2.40E-20	85.44861919
rs7759971	T	C	0.367932	0.000713177	0.000129469	3.60E-08	30.34331812
rs4899257	T	G	0.394594	-0.000698506	0.000127791	4.60E-08	29.87718971

SNP: single nucleotide polymorphism; A1: effect allele; A2: other allele; EAF: effect allele frequency.

Characteristics of the 12 hyperkinetic disorders associated instruments

SNP	A1	A2	EAF	*Beta*	*SE*	*P*-value	*F*-statistic
rs75732251	C	T	0.01158	2.6915	0.5655	1.94E-06	22.65288654
rs2419739	A	T	0.5228	-0.4574	0.0936	1.03E-06	23.88034645
rs150454290	A	G	0.002003	8.2204	1.5951	2.56E-07	26.55889923
rs73126026	G	T	0.1366	0.6645	0.1417	2.72E-06	21.99126794
rs143892063	C	T	0.07183	0.8911	0.1921	3.50E-06	21.51781442
rs77514050	A	G	0.0619	1.0096	0.2135	2.27E-06	22.3616026
rs78858748	A	G	0.06759	1.0097	0.2	4.44E-07	25.48735225
rs148420288	G	A	0.07482	0.8525	0.1857	4.39E-06	21.07487308
rs9579182	A	G	0.2651	0.5337	0.107	6.11E-07	24.87865228
rs755148	T	C	0.1759	0.5864	0.1239	2.22E-06	22.39986034
rs118099629	G	A	0.01935	1.9491	0.4093	1.91E-06	22.67695581
rs62227003	A	G	0.01559	2.4586	0.4726	1.97E-07	27.06377162

SNP: single nucleotide polymorphism; A1: effect allele; A2: other allele; EAF: effect allele frequency.

Characteristics of the 15 anorexia nervosa associated instruments

SNP	A1	A2	*Beta*	*SE*	*P*-value	*F*-statistic
rs145106667	G	A	0.686291	0.1483	3.68E-06	21.41580014
rs61831281	C	G	0.416801	0.0892	2.98E-06	21.83372298
rs75307524	G	A	0.474397	0.101	2.61E-06	22.061809
rs111925923	T	C	0.409497	0.089	4.21E-06	21.17002815
rs13125782	C	T	-0.174802	0.0356	9.19E-07	24.10975508
rs200312312	C	T	-0.180603	0.0335	6.73E-08	29.06432935
rs79350580	T	C	0.309196	0.0666	3.47E-06	21.55357304
rs75544652	T	C	0.5223	0.112	3.10E-06	21.74723294
rs11245454	A	G	-0.154306	0.0333	3.53E-06	21.47223046
rs1894792	T	C	0.172599	0.0364	2.14E-06	22.48401069
rs4622308	T	C	-0.180096	0.0307	4.25E-09	34.41370117
rs11174202	G	A	-0.152798	0.0299	3.11E-07	26.11517634
rs117957029	C	T	0.536502	0.1024	1.62E-07	27.45002708
rs2345557	T	G	0.237299	0.051	3.20E-06	21.64967912
rs75115477	G	A	0.277204	0.0593	3.00E-06	21.85191985

SNP: single nucleotide polymorphism; A1: effect allele; A2: other allele.

Characteristics of the 37 autism spectrum disorder associated instruments

SNP	A1	A2	*Beta*	*SE*	*P*-value	*F*-statistic
rs11185408	A	G	-0.0686965	0.0138	6.98E-07	24.78055614
rs6692705	G	A	-0.0656005	0.0141	3.26E-06	21.64592123
rs78653484	T	C	-0.176296	0.0385	4.67E-06	20.96831143
rs2391769	G	A	0.0769026	0.0145	1.14E-07	28.12846557
rs6701243	C	A	-0.0735014	0.0144	3.07E-07	26.05350985
rs79940520	G	A	0.0953992	0.0207	4.26E-06	21.23971939
rs1452075	T	C	0.080704	0.0155	2.07E-07	27.10982566
rs4916723	C	A	0.067305	0.0141	1.92E-06	22.78538818
rs325485	G	A	-0.0728043	0.0143	3.25E-07	25.92041713
rs72934503	G	A	0.0704976	0.0141	5.89E-07	24.9982979
rs9366877	G	A	-0.0684994	0.0139	9.05E-07	24.28532581
rs9389208	T	C	0.0672006	0.0144	3.12E-06	21.77816667
rs16879023	A	G	-0.0957953	0.0201	1.76E-06	22.71413951
rs740883	T	A	0.113695	0.0238	1.69E-06	22.82069244
rs12203328	C	G	0.0697033	0.0153	4.91E-06	20.75505161
rs111931861	G	A	0.216901	0.0409	1.12E-07	28.12396136
rs7783557	C	T	-0.0670042	0.0146	4.36E-06	21.06193853
rs76397219	G	A	0.140297	0.0303	3.57E-06	21.43934495
rs11787216	T	C	-0.0692	0.0147	2.59E-06	22.16039613
rs10110094	G	A	-0.0906996	0.0191	2.05E-06	22.54986826
rs10099100	C	G	0.0843044	0.0147	1.07E-08	32.89014697
rs28729902	G	A	0.0839035	0.0178	2.34E-06	22.21877702
rs45595836	T	C	0.138996	0.0272	3.13E-07	26.11360296
rs4750990	C	T	0.0680968	0.0141	1.37E-06	23.32465253
rs141319505	G	A	-0.290698	0.061	1.88E-06	22.71038087
rs78827416	A	G	0.130502	0.0266	9.00E-07	24.0697213
rs644552	A	G	0.159403	0.0346	4.21E-06	21.22466204
rs35404050	T	C	0.0843044	0.0176	1.61E-06	22.94431773
rs77691144	C	T	0.207406	0.0435	1.91E-06	22.73338557
rs112635299	T	G	0.220997	0.0432	3.04E-07	26.17009281
rs78058104	A	G	0.187898	0.0397	2.22E-06	22.40078828
rs141455452	G	T	-0.0784044	0.0159	8.94E-07	24.31569139
rs292441	A	G	-0.0724954	0.0149	1.12E-06	23.67273105
rs149923766	G	T	0.237306	0.0484	9.61E-07	24.03957108
rs2224274	T	C	0.0709989	0.0138	2.86E-07	26.46945915
rs910805	A	G	-0.0956963	0.016	2.04E-09	35.77258529
rs144911765	C	T	0.190096	0.0403	2.36E-06	22.25029969

SNP: single nucleotide polymorphism; A1: effect allele; A2: other allele.

Characteristics of the 17 psychoactive substance abuse associated instruments

**SNP**	**A1**	**A2**	**EAF**	***Beta***	***SE***	***P*-value**	***F*-statistic**
rs184795458	A	G	0.039258	-0.00396017	0.000866713	4.90E-06	20.87743007
rs60610159	A	G	0.204638	-0.00208874	0.000405049	2.50E-07	26.59216144
rs62117586	G	A	0.078078	-0.00331335	0.000609221	5.40E-08	29.57909609
rs9851294	C	T	0.054919	0.00339282	0.00071757	2.30E-06	22.35594806
rs10004843	G	C	0.414759	0.00154934	0.000332439	3.20E-06	21.72048569
rs34635	G	A	0.420153	0.00158686	0.000331155	1.70E-06	22.96225802
rs55944202	G	A	0.146009	-0.00214323	0.000463259	3.70E-06	21.40374798
rs75997818	G	C	0.043633	0.0036989	0.000805228	4.40E-06	21.10121412
rs4711634	A	G	0.552571	-0.00153652	0.000328647	2.90E-06	21.85833566
rs234468	G	A	0.664299	-0.00181512	0.000348305	1.90E-07	27.15759244
rs17138597	A	G	0.195447	0.00228458	0.000415081	3.70E-08	30.29333115
rs55805800	A	C	0.113606	0.00263365	0.000521572	4.40E-07	25.4969117
rs61908143	T	C	0.031908	0.00431778	0.000929631	3.40E-06	21.57246761
rs11618413	C	T	0.155253	0.00220164	0.000451385	1.10E-06	23.79021507
rs11672037	C	A	0.101526	-0.00267781	0.000552221	1.20E-06	23.51438811
rs4919820	G	T	0.843735	-0.00231731	0.00045012	2.60E-07	26.50401393
rs6876	C	T	0.1088	-0.00256901	0.000534971	1.60E-06	23.06062689

SNP: single nucleotide polymorphism; A1: effect allele; A2: other allele; EAF: effect allele frequency.

Supplementary file 2

The MR results and scatter plot of bipolar disorders on the risk of sensory, motor, and behavioral disorders.

The univariate MR results of bipolar disorders on the risk of sensory disorders.

Outcome	Method	Bipolar disorders<br>*N* SNV	*OR*（95%*CI*）	*P* value
Pain in the limb	IVW	51	1.00 (95% CI: 0.999-1.001)	0.92
	Weighted median	51	1.00 (95% CI: 0.999-1.002)	0.72
	MR Egger	51	1.00 (95% CI: 0.993-1.007)	0.94
Pruritus	IVW	49	1.29 (95% CI: 1.06-1.57)	0.01
	Weighted median	49	1.38 (95% CI: 1.05-1.81)	0.03
	MR Egger	49	1.48 (95% CI: 0.50-4.32)	0.48
Hearing impairment	IVW	41	1.00 (95% CI: 0.99 - 1.01)	0.92
	Weighted median	41	1.00 (95% CI: 0.99 - 1.02)	0.72
	MR Egger	41	1.05 (95% CI: 0.97 - 1.13)	0.28
Tinnitus	IVW	40	1.00 (95% CI: 0.99 - 1.01)	0.98
	Weighted median	40	1.00 (95% CI: 0.99 - 1.01)	0.76
	MR Egger	40	1.00 (95% CI: 0.97 - 1.03)	0.83
Anosmia	IVW	42	0.83 (95% CI: 0.57 - 1.23)	0.36
	Weighted median	42	1.07 (95% CI: 0.60 - 1.90)	0.82
	MR Egger	42	3.33 (95% CI: 0.42 - 26.50)	0.26
Small fibre neuropathy	IVW	42	1.64 (95% CI: 1.03 - 2.61)	0.04
	Weighted median	42	1.68 (95% CI: 0.85 - 3.32)	0.14
	MR Egger	42	0.41 (95% CI: 0.03 - 5.10)	0.49
Psoriasis	IVW	50	1.00 (95% CI: 0.999 - 1.002)	0.70
	Weighted median	50	1.00 (95% CI: 0.998 - 1.001)	0.82
	MR Egger	50	0.41 (95% CI: 0.988 - 1.003)	0.27

Abbreviations: MR, Mendelian randomization; N SNV, number of single-nucleotide variants; IVW, inverse-variance weighted; OR, odds ratio.

Genetic instruments selected from bipolar disorders GWASs, selection threshold P less than 5×10^{-8}, pruned at linkage disequilibrium R^2 less than 0.001 (10,000 kilobase pair window).

The univariate MR results of bipolar disorders on the risk of motor and behavioral disorders.

Outcome	Method	Bipolar disorders		
		N SNV	*OR*（95%*CI*）	*P* value
Extrapyramidal and movement disorders	IVW	42	1.05 (95% CI: 0.94 - 1.17)	0.40
	Weighted median	42	1.07 (95% CI: 0.92 - 1.25)	0.37
	MR Egger	42	1.21 (95% CI: 0.67 - 2.18)	0.53
Multiple Sclerosis	IVW	35	1.000 (95% CI: 0.999 - 1.001)	0.68
	Weighted median	35	1.001 (95% CI: 1.000 - 1.002)	0.31
	MR Egger	35	1.004 (95% CI: 0.998 - 1.011)	0.20
Hyperkinetic disorders	IVW	42	2.019 (95% CI: 1.263 - 3.229)	0.003
	Weighted median	42	2.565 (95% CI: 1.329 - 4.950)	0.005
	MR Egger	42	1.647 (95% CI: 0.132 - 20.620)	0.70
Anorexia nervosa	IVW	44	1.19 (95% CI: 1.01 - 1.40)	0.04
	Weighted median	44	1.21 (95% CI: 0.98 - 1.50)	0.08
	MR Egger	44	1.00 (95% CI: 0.38 - 2.63)	0.99
Autism spectrum disorder	IVW	39	1.10 (95% CI: 1.01 - 1.20)	0.03
	Weighted median	39	1.07 (95% CI: 0.95 - 1.20)	0.26
	MR Egger	39	0.86 (95% CI: 0.53 - 1.39)	0.53
Psychoactive substance abuse	IVW	44	0.998 (95% CI: 0.997 - 1.000)	0.04
	Weighted median	44	0.998 (95% CI: 0.996 - 1.000)	0.09
	MR Egger	44	0.995 (95% CI: 0.985 - 1.004)	0.28

Abbreviations: MR, Mendelian randomization; N SNV, number of single-nucleotide variants; IVW, inverse-variance weighted; OR, odds ratio.

Genetic instruments selected from bipolar disorders GWASs, selection threshold *P* less than 5×10^{-8}, pruned at linkage disequilibrium R^2 less than 0.001 (10,000 kilobase pair window).

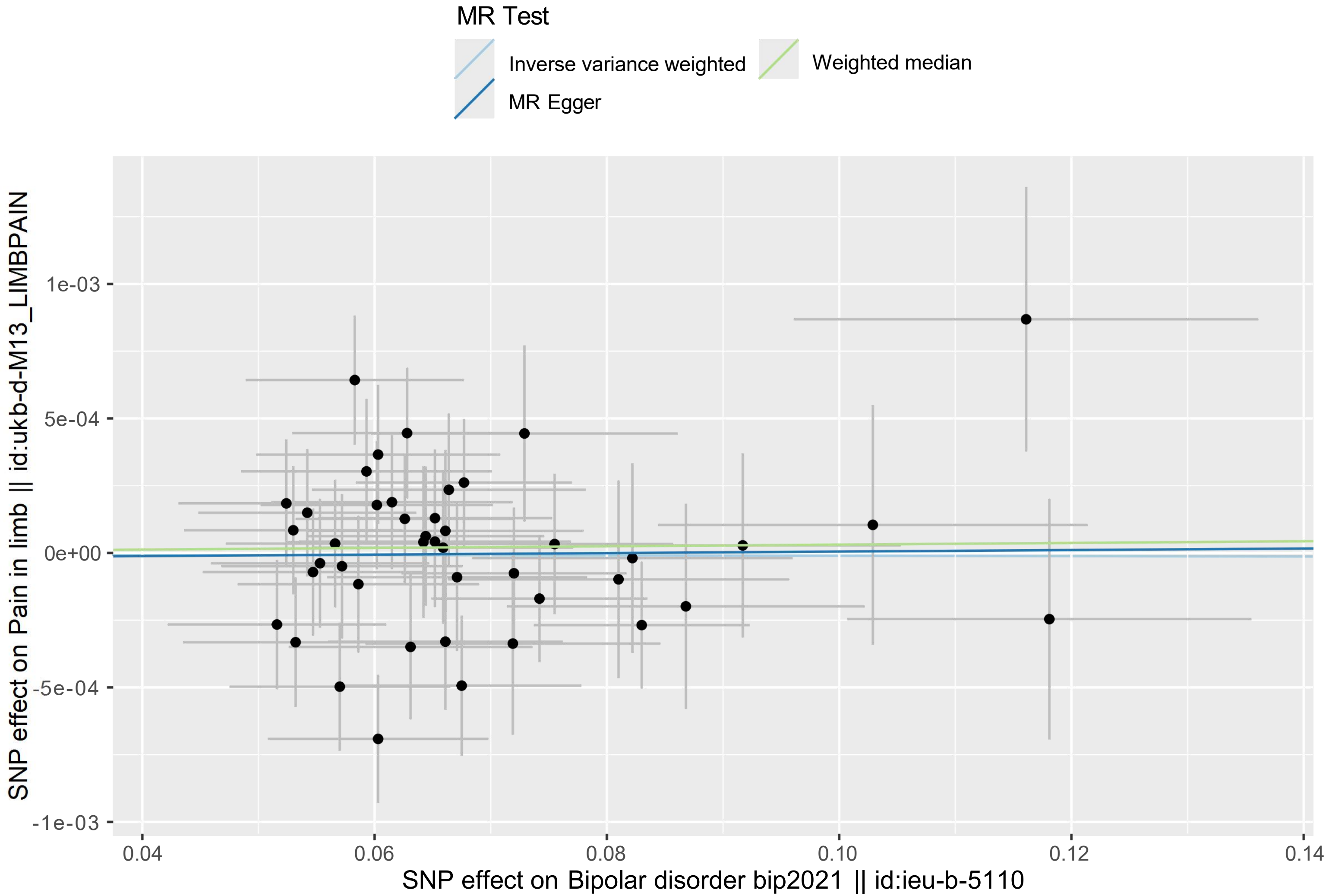
MR Test
Inverse variance weighted
MR Egger
Weighted median
1e-03
5e-04
0e+00
-5e-04
-1e-03
SNP effect on Pain in limb || id:ukb-d-M13_LIMBPAIN
0.04
0.06
0.08
0.10
0.12
0.14
SNP effect on Bipolar disorder bip2021 || id:ieu-b-5110

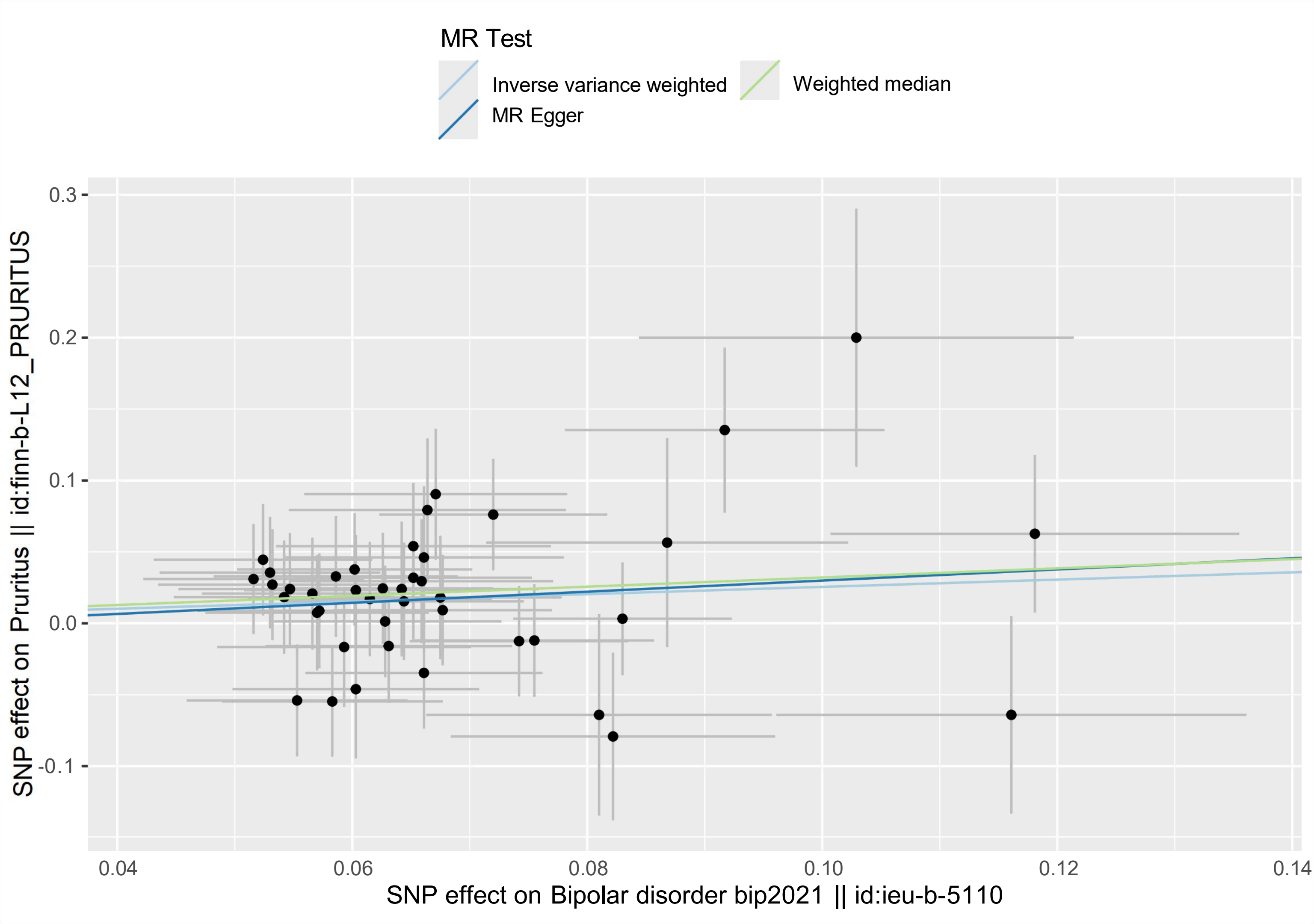
MR Test
Inverse variance weighted
MR Egger
Weighted median
SNP effect on Pruritus || id:finn-b-L12_PRURITUS
SNP effect on Bipolar disorder bip2021 || id:ieu-b-5110
0.3
0.2
0.1
0.0
-0.1
0.04
0.06
0.08
0.10
0.12
0.14

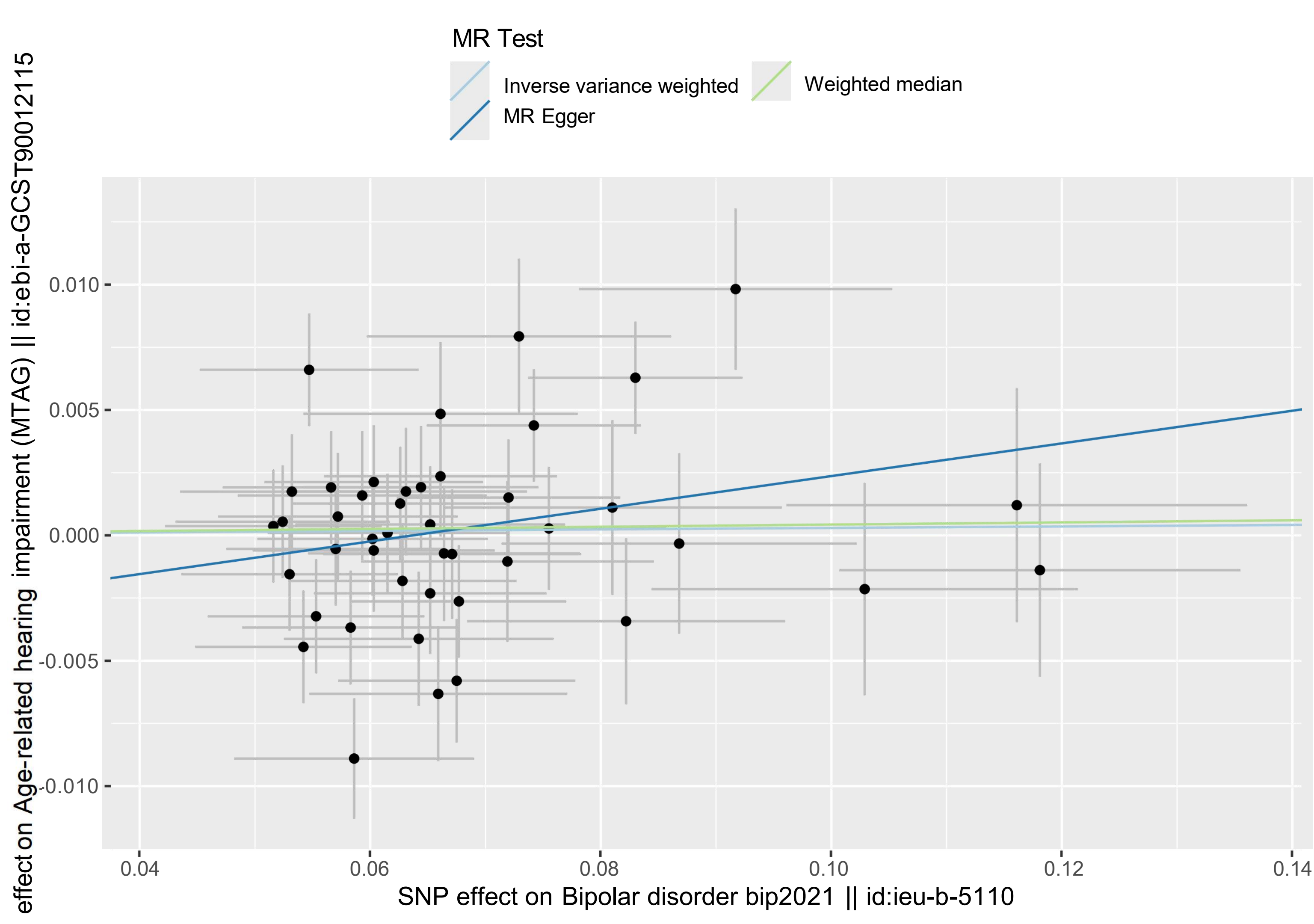

MR Test
Inverse variance weighted
MR Egger
Weighted median
0.010
0.005
0.000
-0.005
-0.010
0.04
0.06
0.08
0.10
0.12
0.14
SNP effect on Bipolar disorder bip2021 || id:ieu-b-5110
effect on Age-related hearing impairment (MTAG) || id:ebi-a-GCST90012115

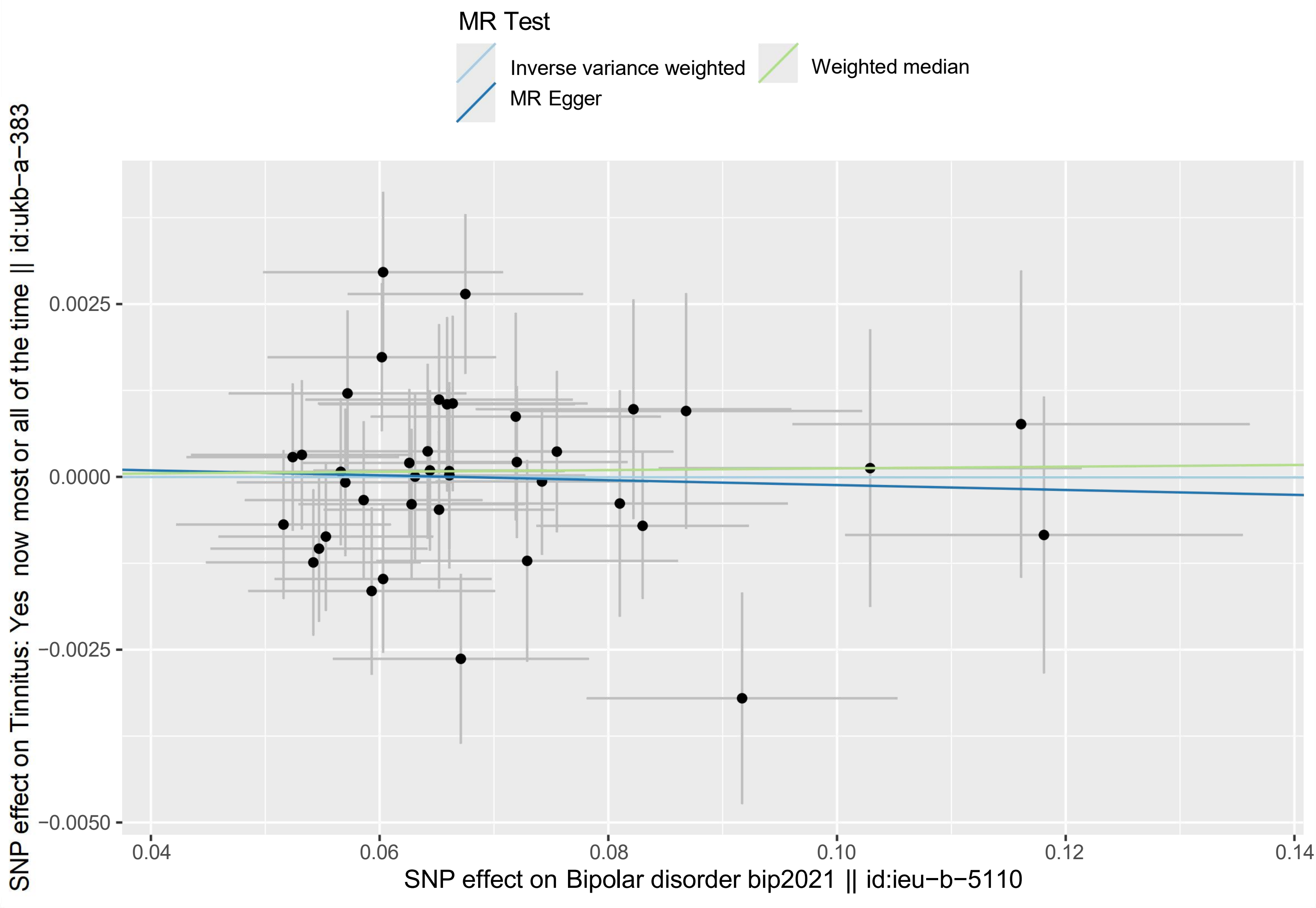
MR Test
Inverse variance weighted
MR Egger
Weighted median
SNP effect on Tinnitus: Yes now most or all of the time || id:ukb-a-383
0.0025
0.0000
−0.0025
−0.0050
0.04
0.06
0.08
0.10
0.12
0.14
SNP effect on Bipolar disorder bip2021 || id:ieu−b−5110

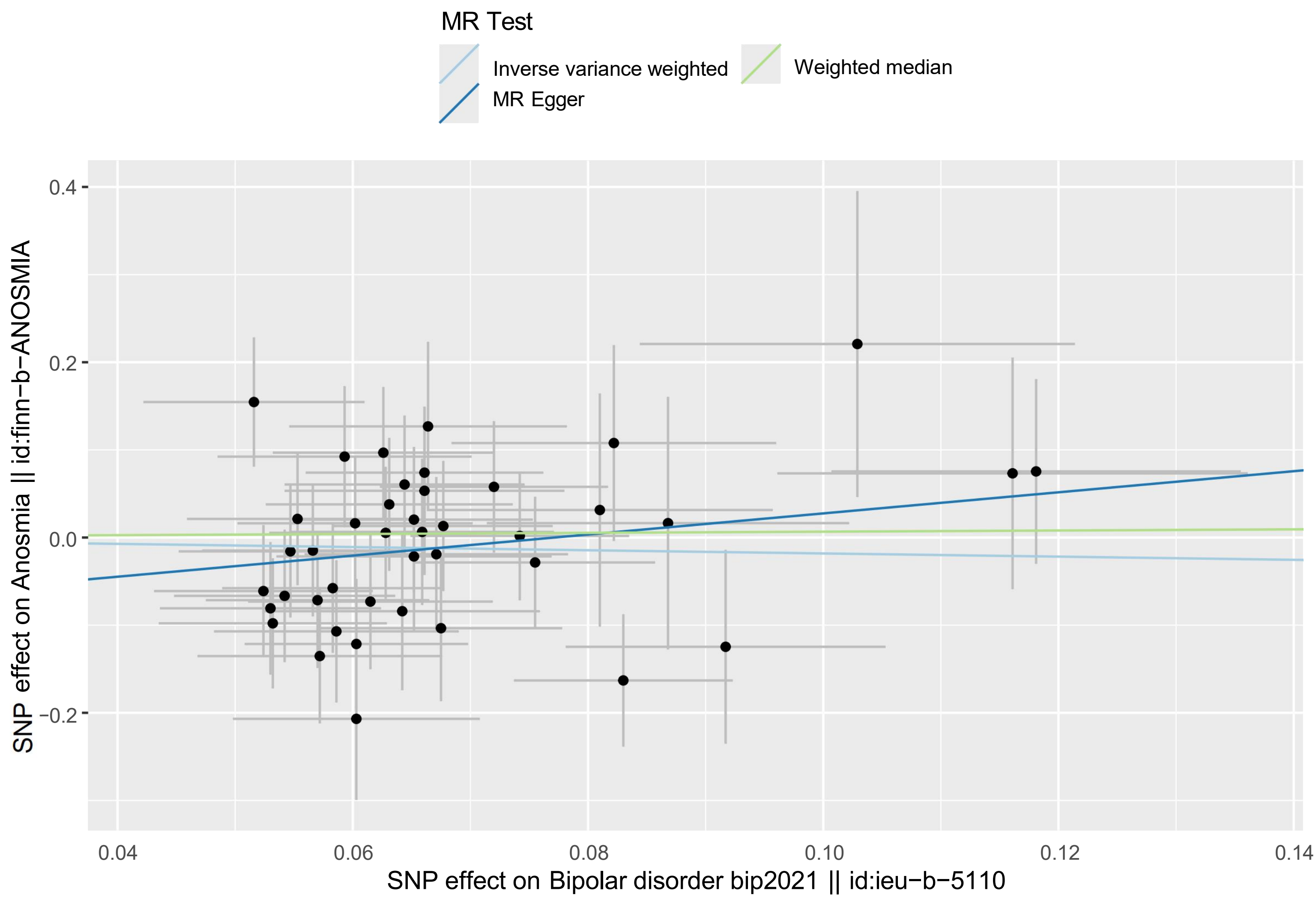

MR Test
Inverse variance weighted
MR Egger
Weighted median
0.4
0.2
0.0
−0.2
SNP effect on Anosmia || id:finn−b−ANOSMIA
0.04
0.06
0.08
0.10
0.12
0.14
SNP effect on Bipolar disorder bip2021 || id:ieu−b−5110

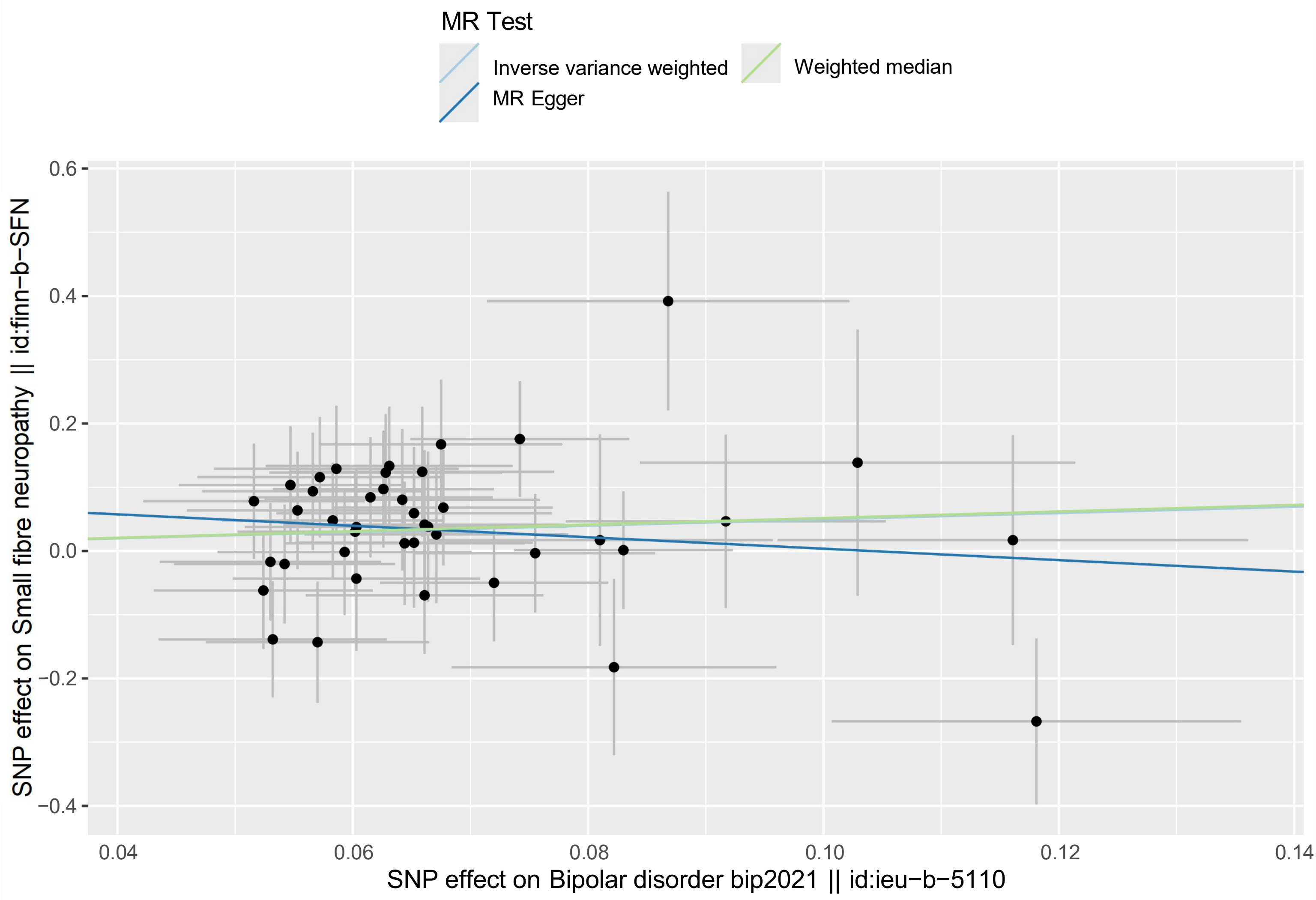

MR Test
Inverse variance weighted
MR Egger
Weighted median
SNP effect on Small fibre neuropathy || id:finn-b-SFN
SNP effect on Bipolar disorder bip2021 || id:ieu-b-5110
0.6
0.4
0.2
0.0
−0.2
−0.4
0.04
0.06
0.08
0.10
0.12
0.14

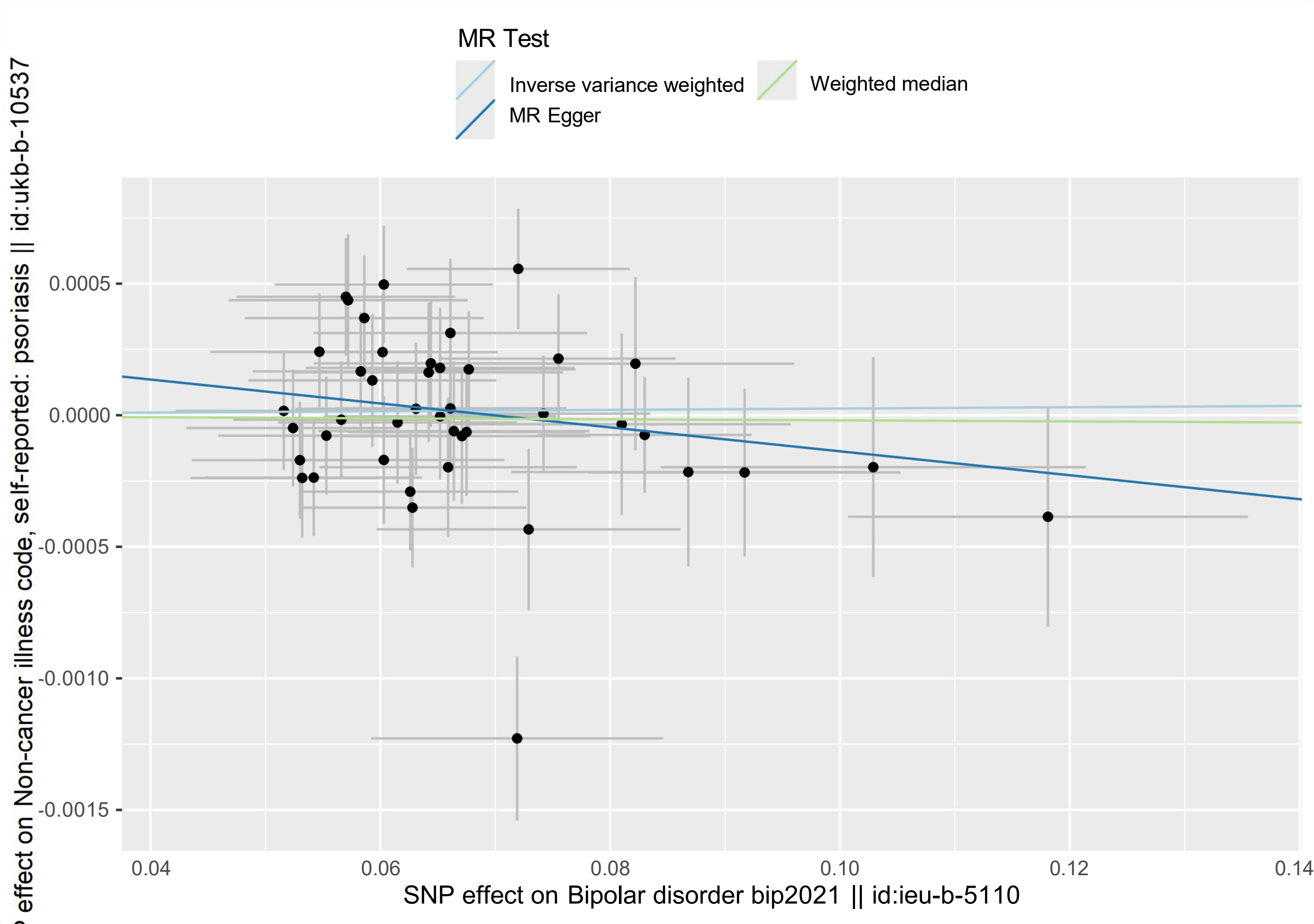
MR Test
Inverse variance weighted
MR Egger
Weighted median
0.0005
0.0000
-0.0005
-0.0010
-0.0015
0.04
0.06
0.08
0.10
0.12
0.14
SNP effect on Bipolar disorder bip2021 || id:ieu-b-5110
SNP effect on Non-cancer illness code, self-reported: psoriasis || id:ukb-b-10537

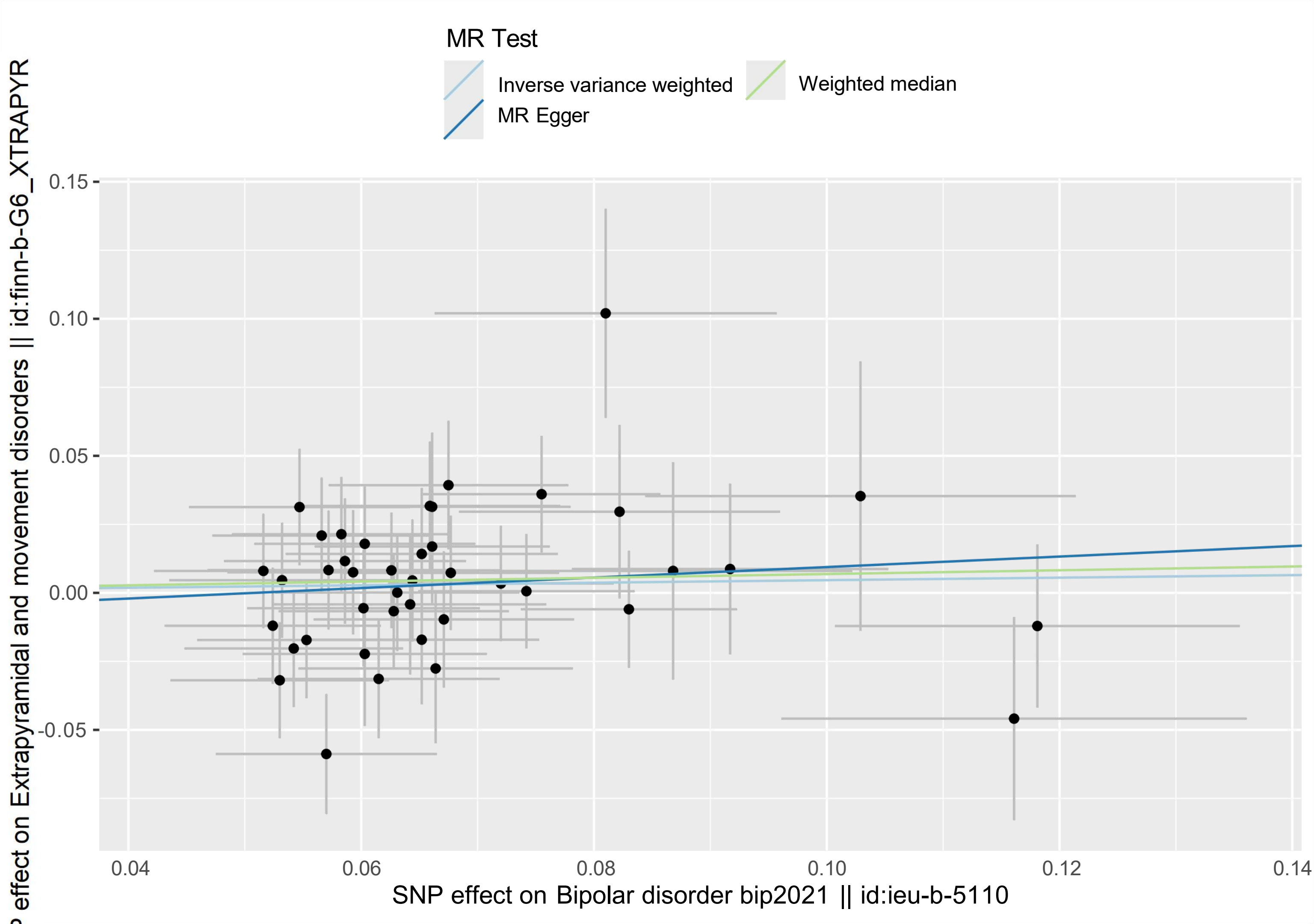
MR Test
Inverse variance weighted
MR Egger
Weighted median
0.15
0.10
0.05
0.00
-0.05
0.04
0.06
0.08
0.10
0.12
0.14
SNP effect on Bipolar disorder bip2021 || id:ieu-b-5110
P effect on Extrapyramidal and movement disorders || id:finn-b-G6_XTRAPYR

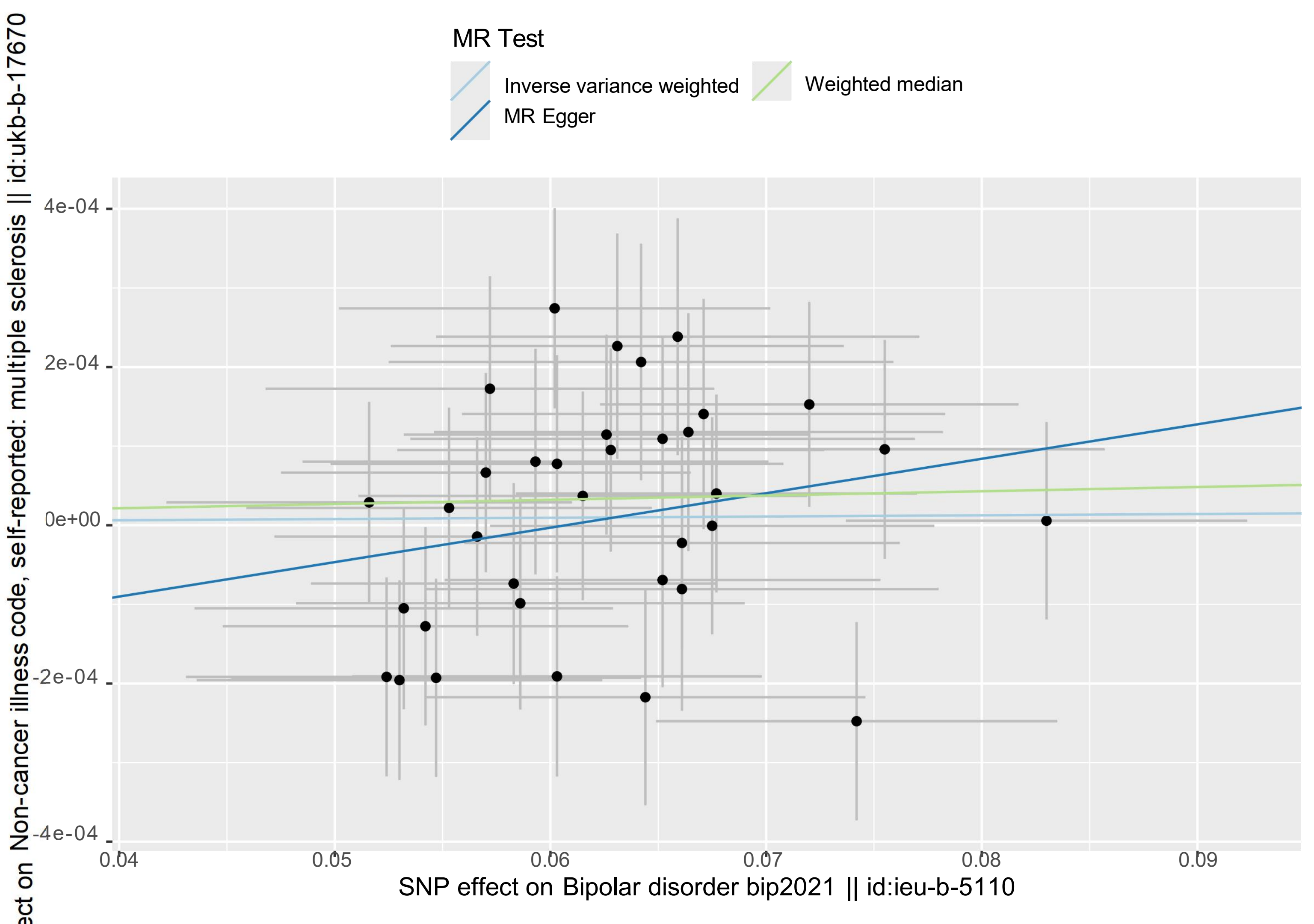

MR Test
Inverse variance weighted
MR Egger
Weighted median
4e-04
2e-04
0e+00
-2e-04
-4e-04
0.04
0.05
0.06
0.07
0.08
0.09
SNP effect on Bipolar disorder bip2021 || id:ieu-b-5110
Non-cancer illness code, self-reported: multiple sclerosis || id:ukb-b-17670
ect on

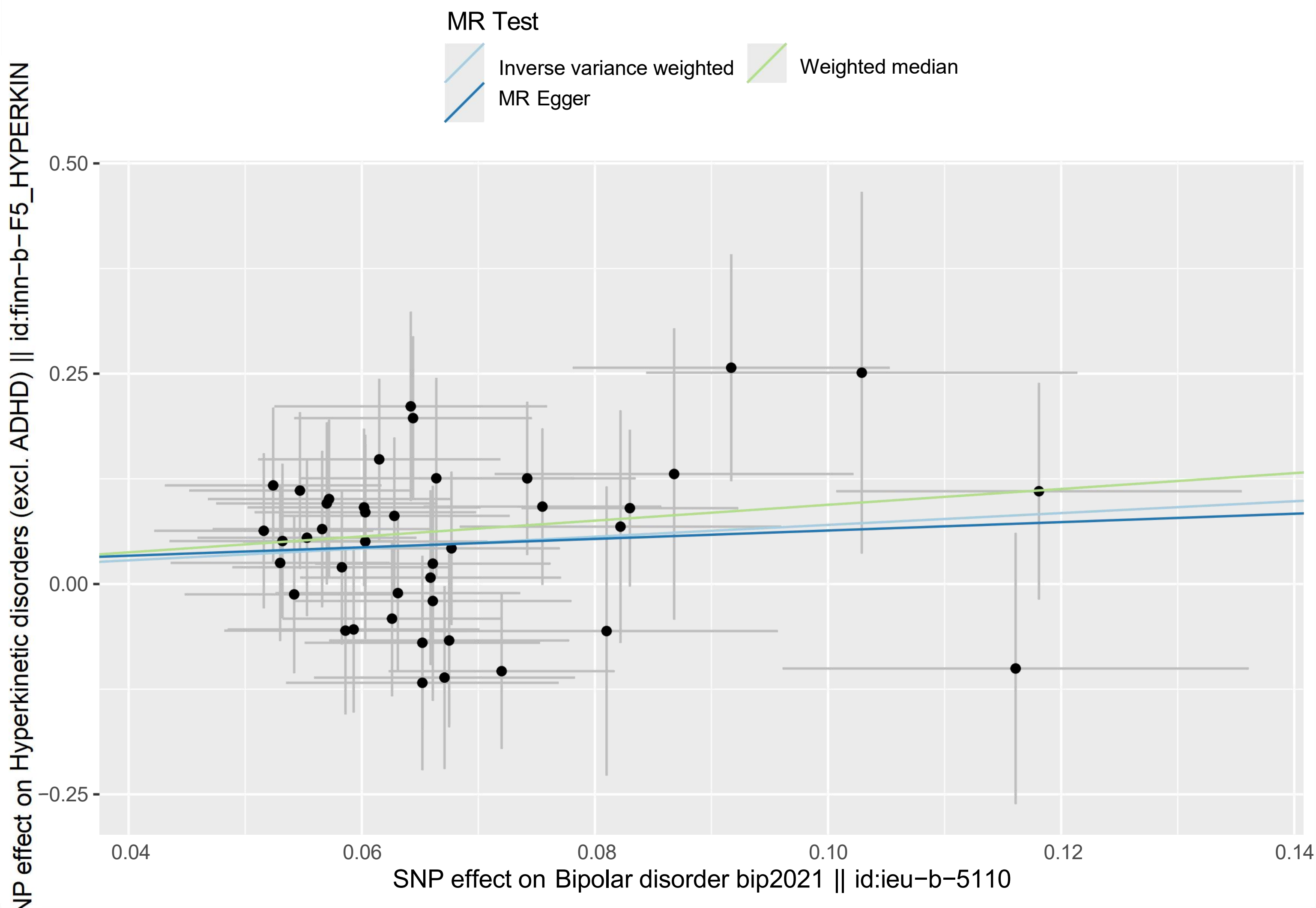

MR Test
Inverse variance weighted
MR Egger
Weighted median
0.50
0.25
0.00
−0.25
0.04
0.06
0.08
0.10
0.12
0.14
SNP effect on Bipolar disorder bip2021 || id:ieu−b−5110
NP effect on Hyperkinetic disorders (excl. ADHD) || id:finn−b−F5_HYPERKIN

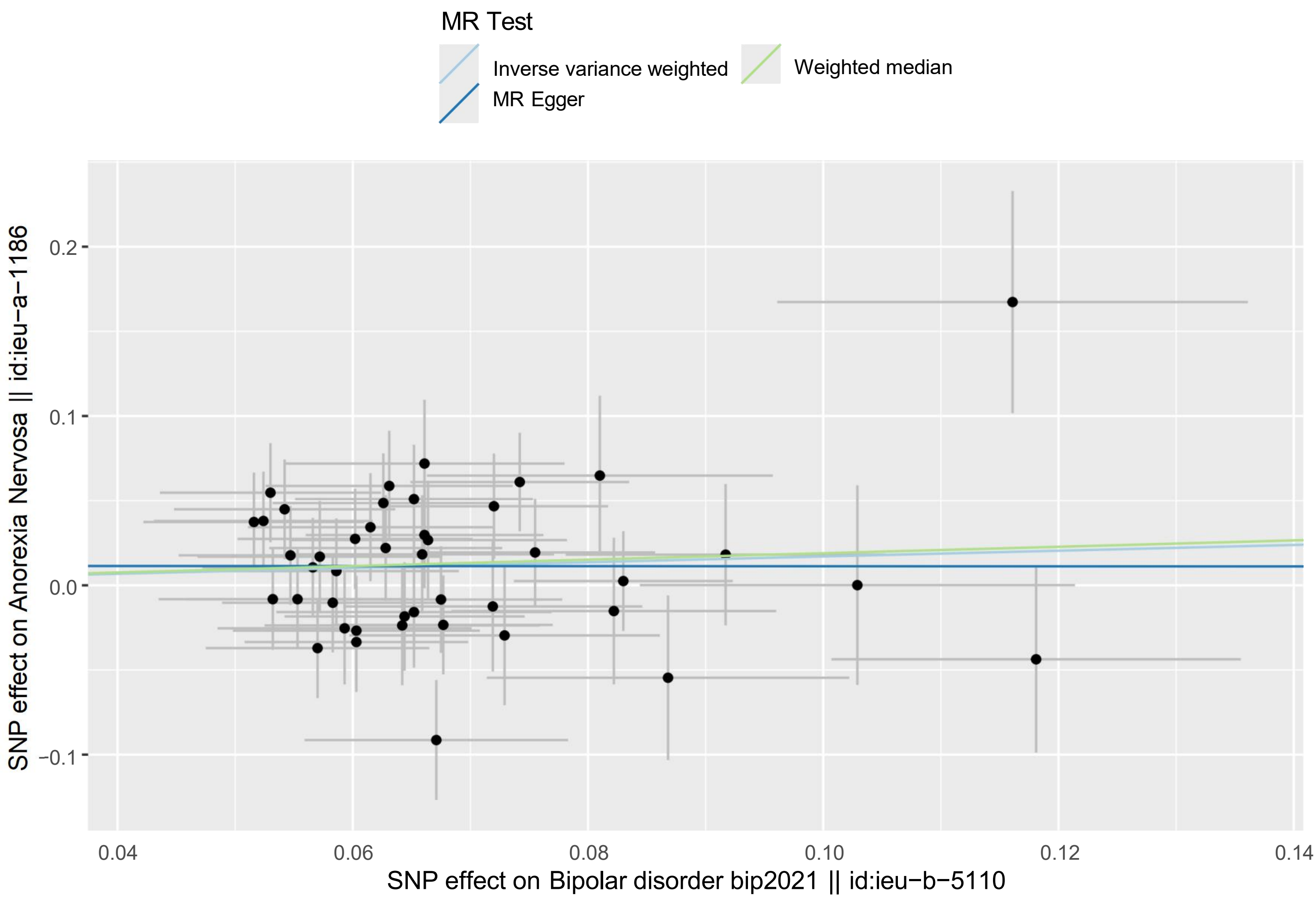
MR Test
Inverse variance weighted
MR Egger
Weighted median
SNP effect on Anorexia Nervosa || id:ieu-a-1186
0.2
0.1
0.0
−0.1
0.04
0.06
0.08
0.10
0.12
0.14
SNP effect on Bipolar disorder bip2021 || id:ieu−b−5110

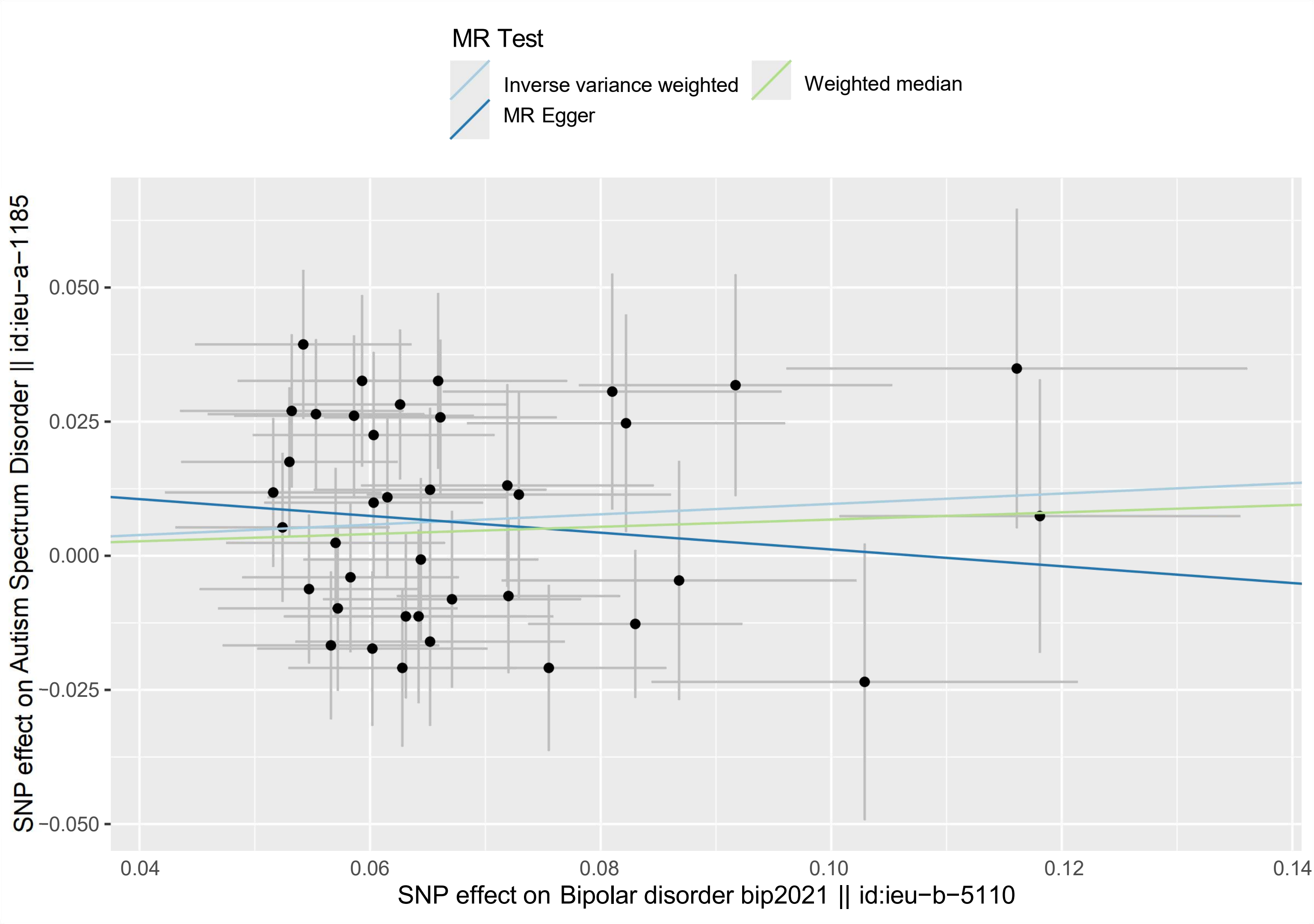
MR Test
Inverse variance weighted
MR Egger
Weighted median
0.050
0.025
0.000
−0.025
−0.050
0.04
0.06
0.08
0.10
0.12
0.14
SNP effect on Bipolar disorder bip2021 || id:ieu−b−5110
SNP effect on Autism Spectrum Disorder || id:ieu−a−1185

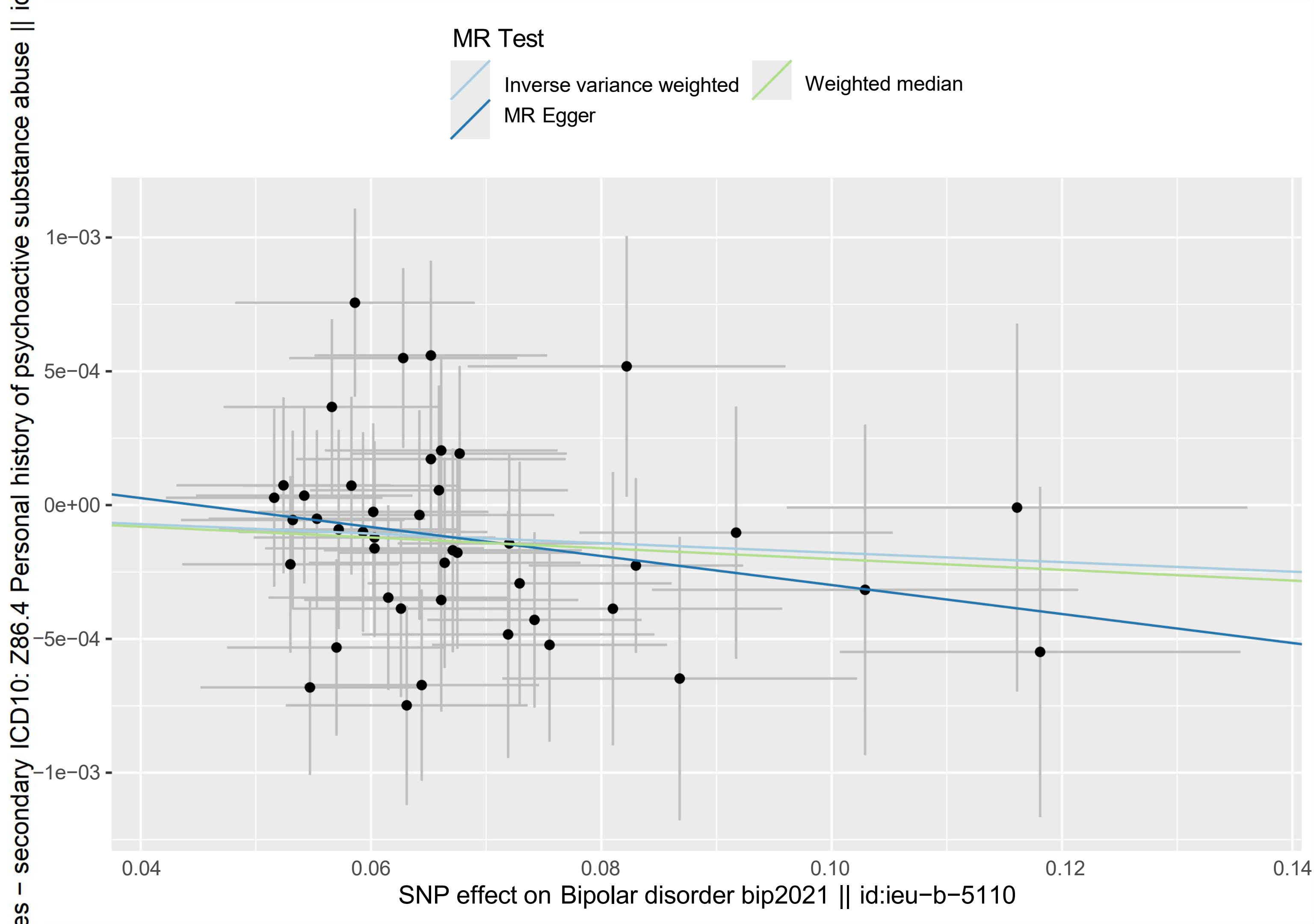

MR Test
Inverse variance weighted
MR Egger
Weighted median
1e−03
5e−04
0e+00
−5e−04
−1e−03
0.04
0.06
0.08
0.10
0.12
0.14
SNP effect on Bipolar disorder bip2021 || id:ieu−b−5110
es − secondary ICD10: Z86.4 Personal history of psychoactive substance abuse || id

Supplementary file 3

The MR results and scatter plot of sensory, motor, and behavioral disorders on the risk of bipolar disorders.

The univariate MR results of sensory disorders on the risk of bipolar disorders.

Exposure	Method	Bipolar disorders<br>*N* SNV	*OR*（95%*CI*）	*P* value
* Pain in the limb	IVW	10	280.69 (95% CI: 0.07 – 1.06E+06)	0.18
	Weighted median	10	195.51 (95% CI: 0.03 – 1.16E+06)	0.23
	MR Egger	10	757706.3 (95% CI: 0.0002 – 2.44E+15)	0.29
* Pruritus	IVW	7	1.04 (95% CI: 1.01 – 1.08)	0.007
	Weighted median	7	1.05 (95% CI: 1.01 – 1.10)	0.024
	MR Egger	7	1.01 (95% CI: 0.95 – 1.07)	0.749
Hearing impairment	IVW	27	0.83 (95% CI: 0.62 – 1.10)	0.92
	Weighted median	27	0.80 (95% CI: 0.56 – 1.15)	0.23
	MR Egger	27	1.44 (95% CI: 0.45 – 4.62)	0.55
* Tinnitus	IVW	15	0.49 (95% CI: 0.16 – 1.53)	0.22
	Weighted median	15	0.45 (95% CI: 0.10 – 1.93)	0.28
	MR Egger	15	0.73 (95% CI: 0.03 – 16.97)	0.85
* Anosmia	IVW	11	1.013 (95% CI: 1.000 – 1.026)	0.055
	Weighted median	11	1.011 (95% CI: 0.993 – 1.028)	0.238
	MR Egger	11	1.016 (95% CI: 0.992 – 1.040)	0.237
* Small fibre neuropathy	IVW	6	1.01 (95% CI: 0.99 – 1.02)	0.32
	Weighted median	6	1.01 (95% CI: 0.99 – 1.03)	0.30
	MR Egger	6	1.00 (95% CI: 0.98 – 1.02)	0.86
Psoriasis	IVW	22	9.97 (95% CI: 1.85 – 53.67)	0.007
	Weighted median	22	10.55 (95% CI: 2.55 – 43.64)	0.002
	MR Egger	22	10.56 (95% CI: 1.19 – 93.72)	0.05

Abbreviations: MR, Mendelian randomization; N SNV, number of single-nucleotide variants; IVW, inverse-variance weighted; OR, odds ratio.

Genetic instruments selected from bipolar disorders GWASs, selection threshold *P* less than 5×10^{-8}, pruned at linkage disequilibrium R^2 less than 0.001 (10,000 kilobase pair window).

* If the number of SNPs available for analysis is less than 3, the selection threshold *P* will be adjusted to 5×10^{-6}.

The univariate MR results of motor and behavioral disorders on the risk of bipolar disorders.

**Exposure**	**Method**	**Bipolar disorders** *N* SNV	***OR*（95%*CI*）**	***P* value**
* Extrapyramidal and movement disorders	IVW	6	0.876 (95% CI: 0.798 – 0.961)	0.005
	Weighted median	6	0.913 (95% CI: 0.818 – 1.019)	0.106
	MR Egger	6	1.374 (95% CI: 0.495 – 3.809)	0.575
Multiple Sclerosis	IVW	5	1.03 (95% CI: 2.4E-05 – 4.3E+04)	1.00
	Weighted median	5	1.93 (95% CI: 5.6E-05 – 6.7E+04)	0.90
	MR Egger	5	55.06 (95% CI: 1.3E-08 – 2.4E+11)	0.75
* Hyperkinetic disorders	IVW	8	1.00 (95% CI: 0.99 – 1.02)	0.76
	Weighted median	8	1.00 (95% CI: 0.99 – 1.02)	0.72
	MR Egger	8	1.01 (95% CI: 0.98 – 1.03)	0.69
* Anorexia nervosa	IVW	12	1.02 (95% CI: 0.98 – 1.06)	0.30
	Weighted median	12	1.02 (95% CI: 0.97 – 1.07)	0.50
	MR Egger	12	1.01 (95% CI: 0.92 – 1.11)	0.83
* Autism spectrum disorder	IVW	27	1.05 (95% CI: 0.99 – 1.11)	0.09
	Weighted median	27	1.01 (95% CI: 0.94 – 1.09)	0.85
	MR Egger	27	1.09 (95% CI: 0.93 – 1.28)	0.30
* Psychoactive substance abuse	IVW	10	0.35 (95% CI: 6.4E-03 – 1.9E+01)	0.61
	Weighted median	10	0.44 (95% CI: 2.9E-03 – 6.8E+01)	0.75
	MR Egger	10	1.04E+03 (95% CI: 1.7E-03 – 6.6E+08)	0.34

Abbreviations: MR, Mendelian randomization; N SNV, number of single-nucleotide variants; IVW, inverse-variance weighted; OR, odds ratio.

Genetic instruments selected from bipolar disorders GWASs, selection threshold *P* less than 5×10^{-8}, pruned at linkage disequilibrium R^2 less than 0.001 (10,000 kilobase pair window).

* If the number of SNPs available for analysis is less than 3, the selection threshold *P* will be adjusted to 5×10^{-6}.

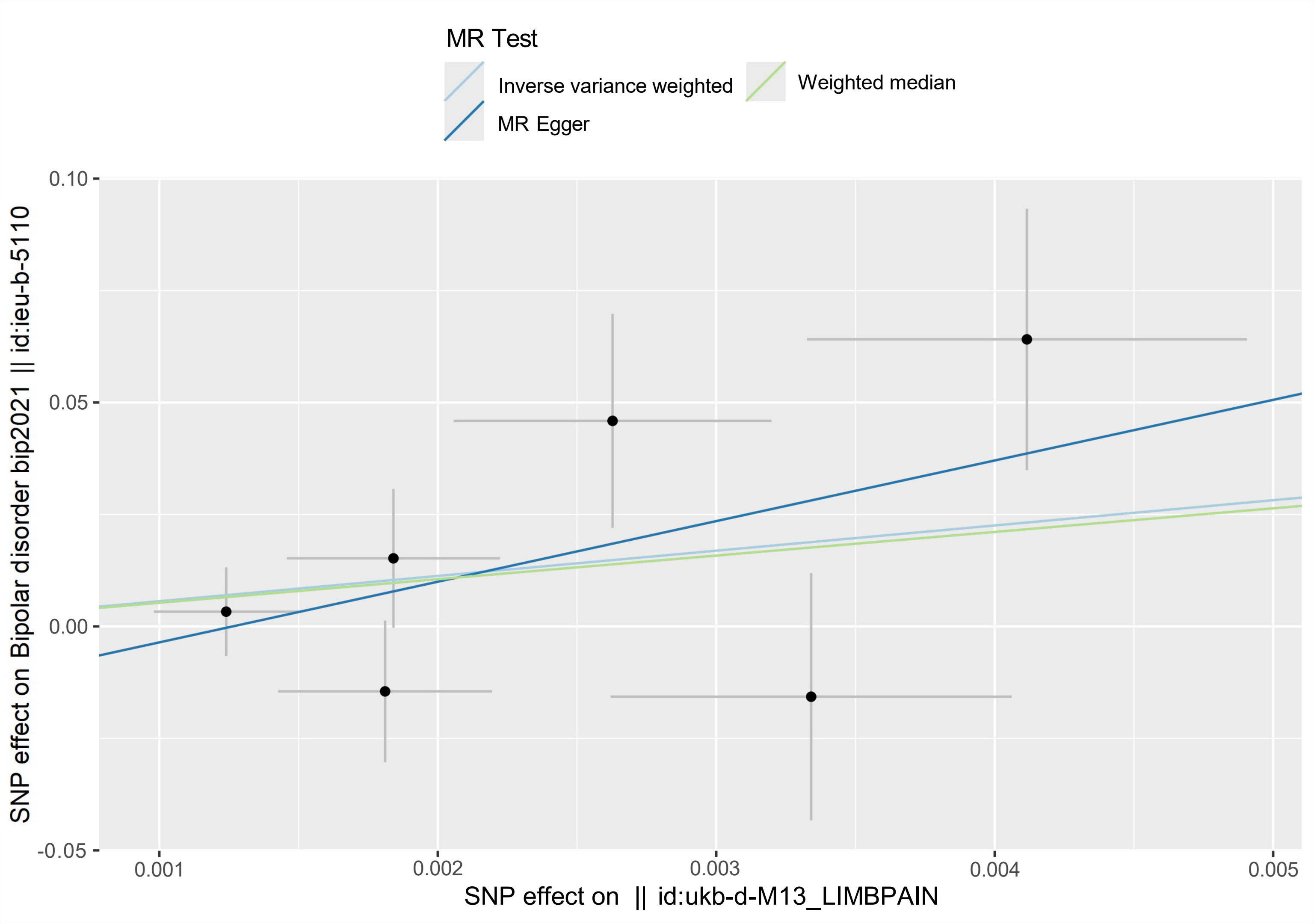

MR Test
Inverse variance weighted
MR Egger
Weighted median
0.10
0.05
0.00
-0.05
0.001
0.002
0.003
0.004
0.005
SNP effect on Bipolar disorder bip2021 || id:ieu-b-5110
SNP effect on || id:ukb-d-M13_LIMBPAIN

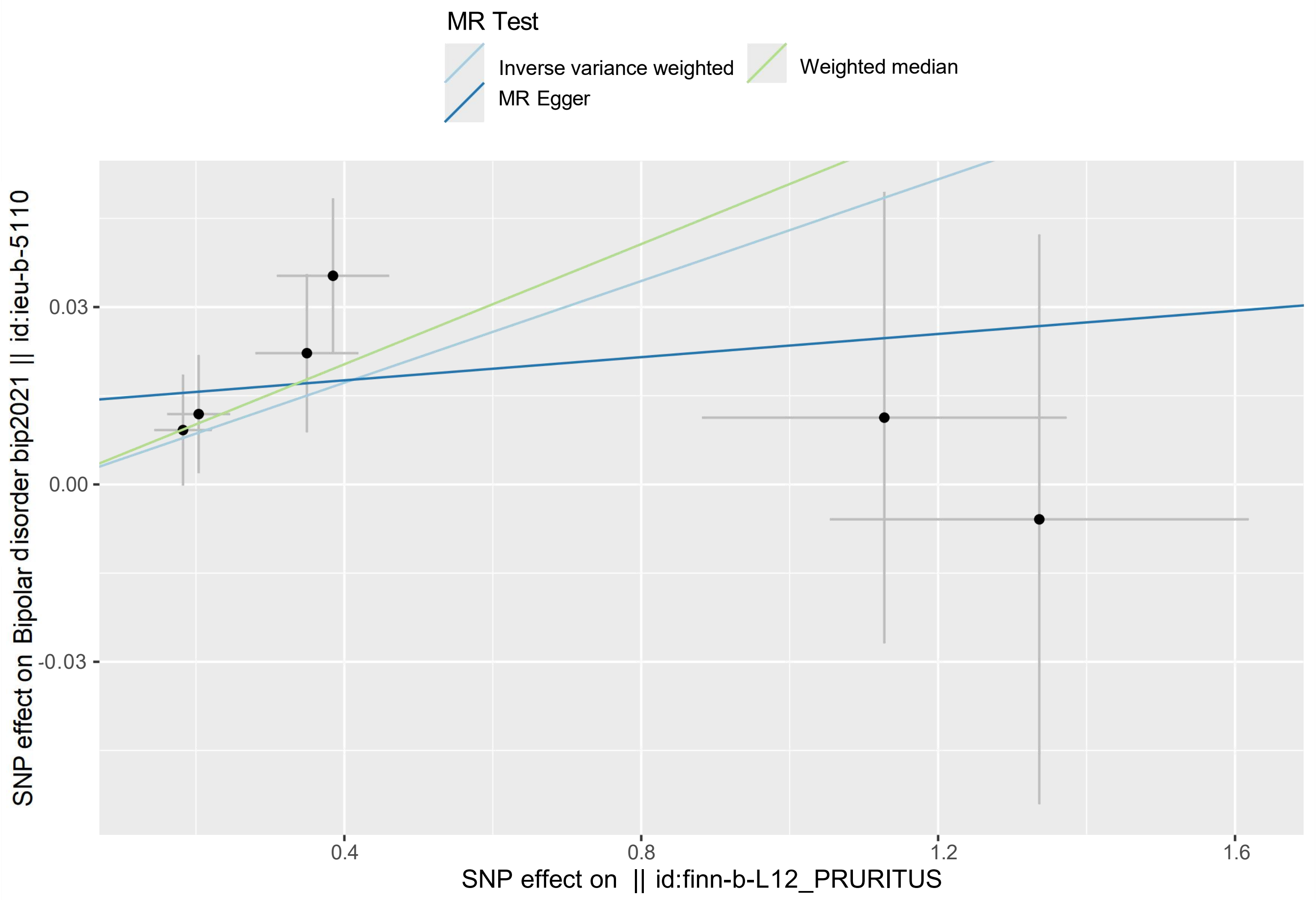
MR Test
Inverse variance weighted
MR Egger
Weighted median
SNP effect on Bipolar disorder bip2021 || id:ieu-b-5110
0.03
0.00
-0.03
0.4
0.8
1.2
1.6
SNP effect on || id:finn-b-L12_PRURITUS

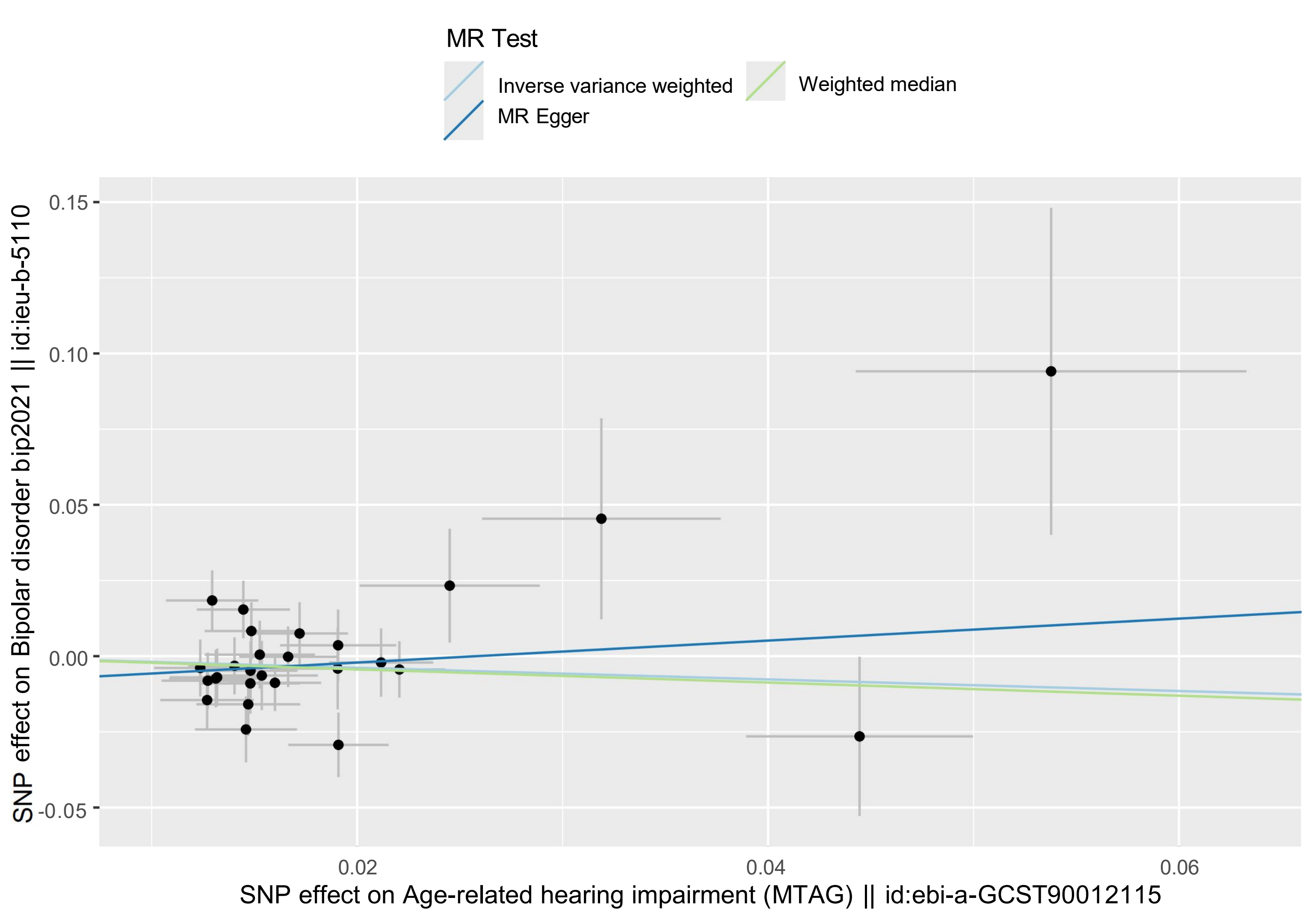
MR Test
Inverse variance weighted
MR Egger
Weighted median
0.15
0.10
0.05
0.00
-0.05
0.02
0.04
0.06
SNP effect on Bipolar disorder bip2021 || id:ieu-b-5110
SNP effect on Age-related hearing impairment (MTAG) || id:ebi-a-GCST90012115

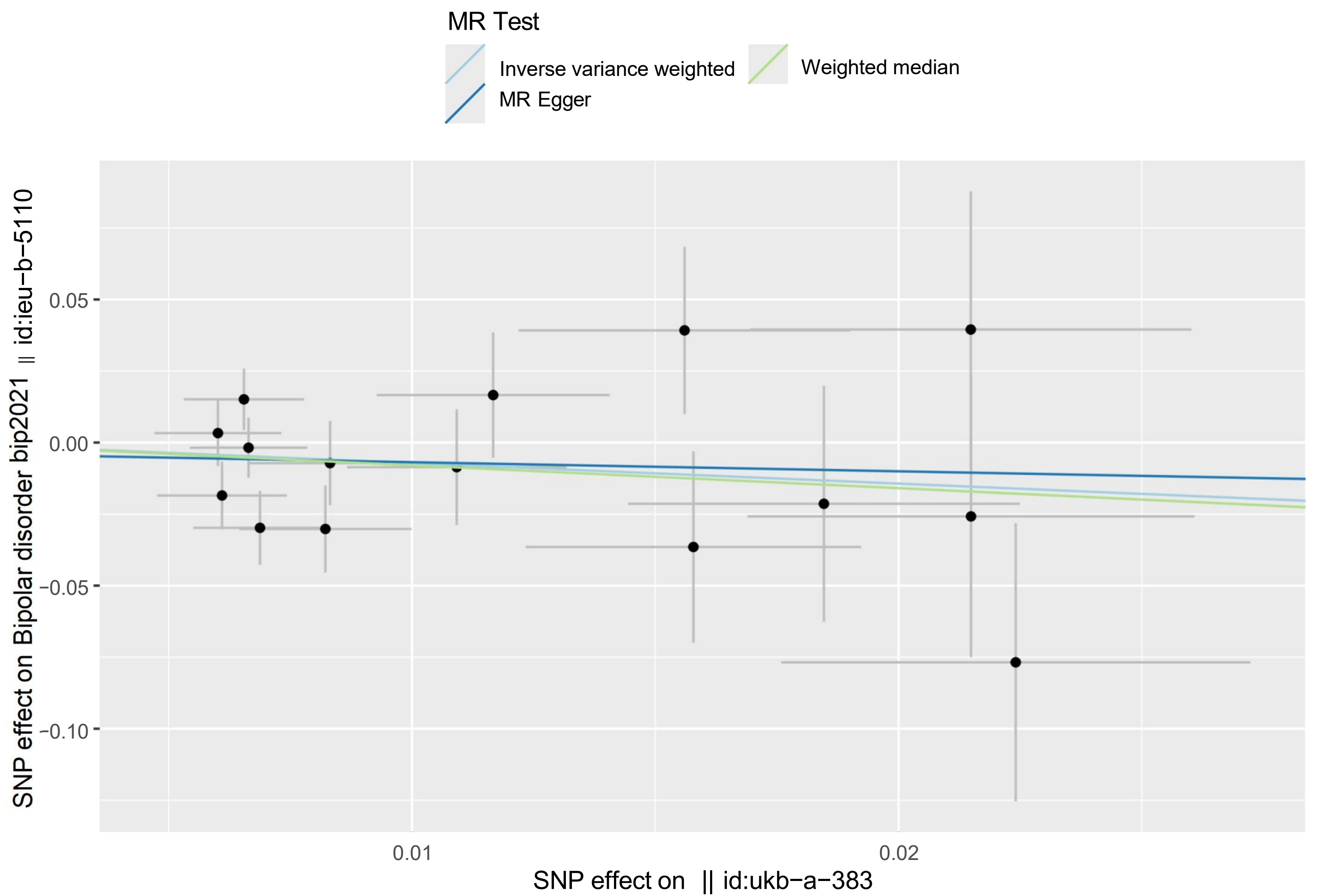

MR Test
Inverse variance weighted
MR Egger
Weighted median
0.05
0.00
−0.05
−0.10
0.01
0.02
SNP effect on Bipolar disorder bip2021 || id:ieu-b-5110
SNP effect on || id:ukb-a-383

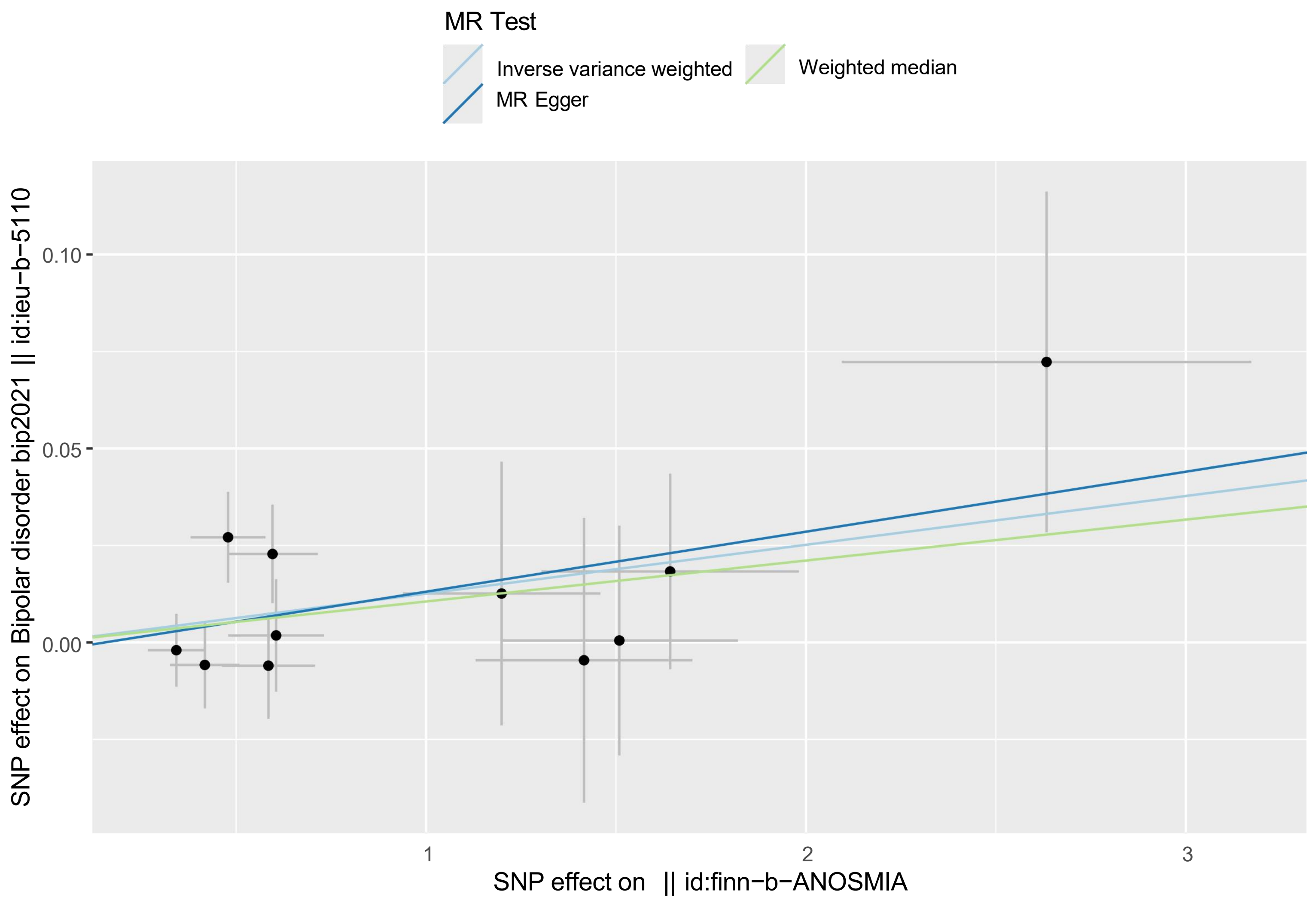
MR Test
Inverse variance weighted
MR Egger
Weighted median
0.10
0.05
0.00
1
2
3
SNP effect on Bipolar disorder bip2021 || id:ieu-b-5110
SNP effect on || id:finn-b-ANOSMIA

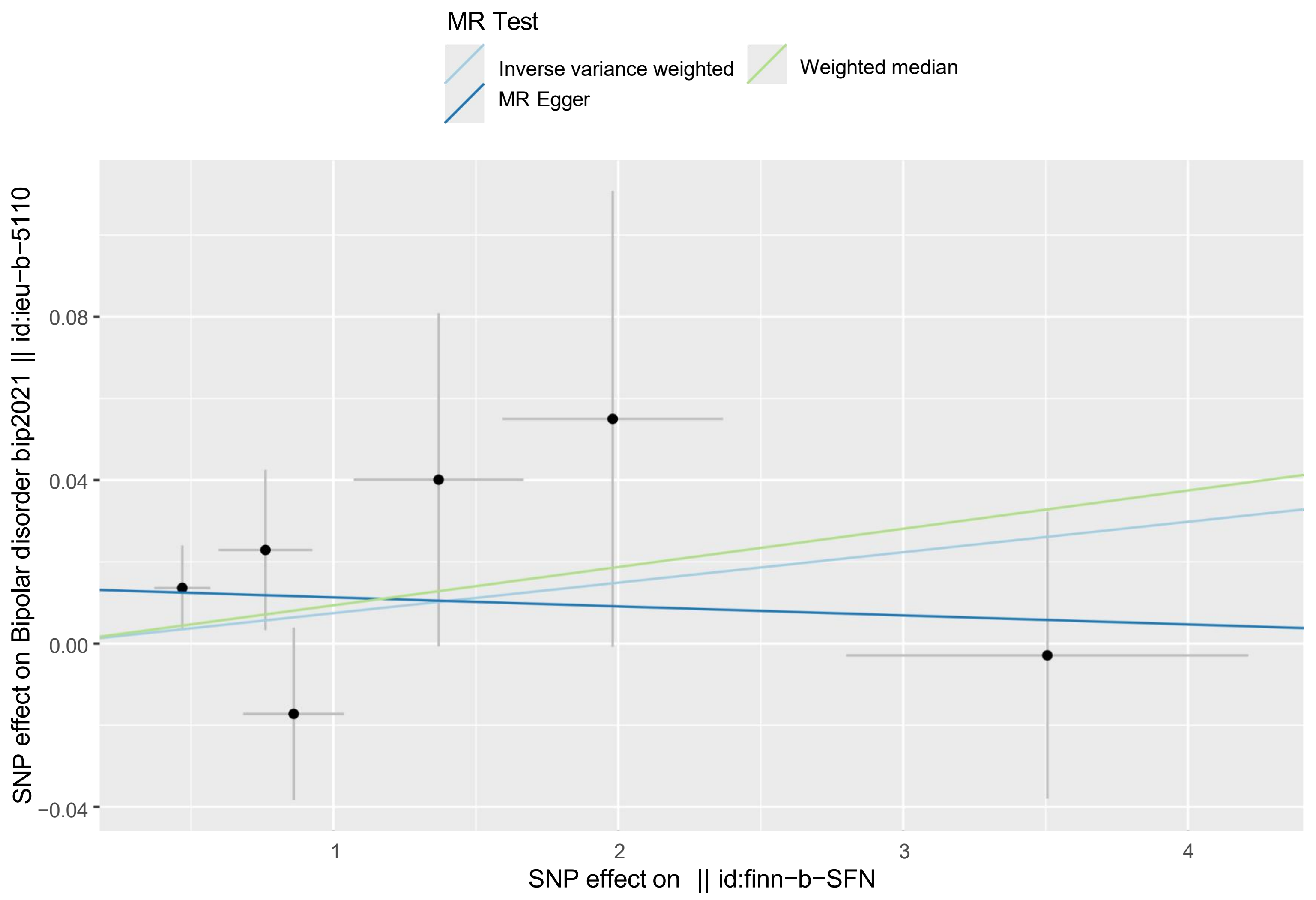
MR Test
Inverse variance weighted
MR Egger
Weighted median
SNP effect on Bipolar disorder bip2021 || id:ieu−b−5110
0.08
0.04
0.00
−0.04
1
2
3
4
SNP effect on || id:finn−b−SFN

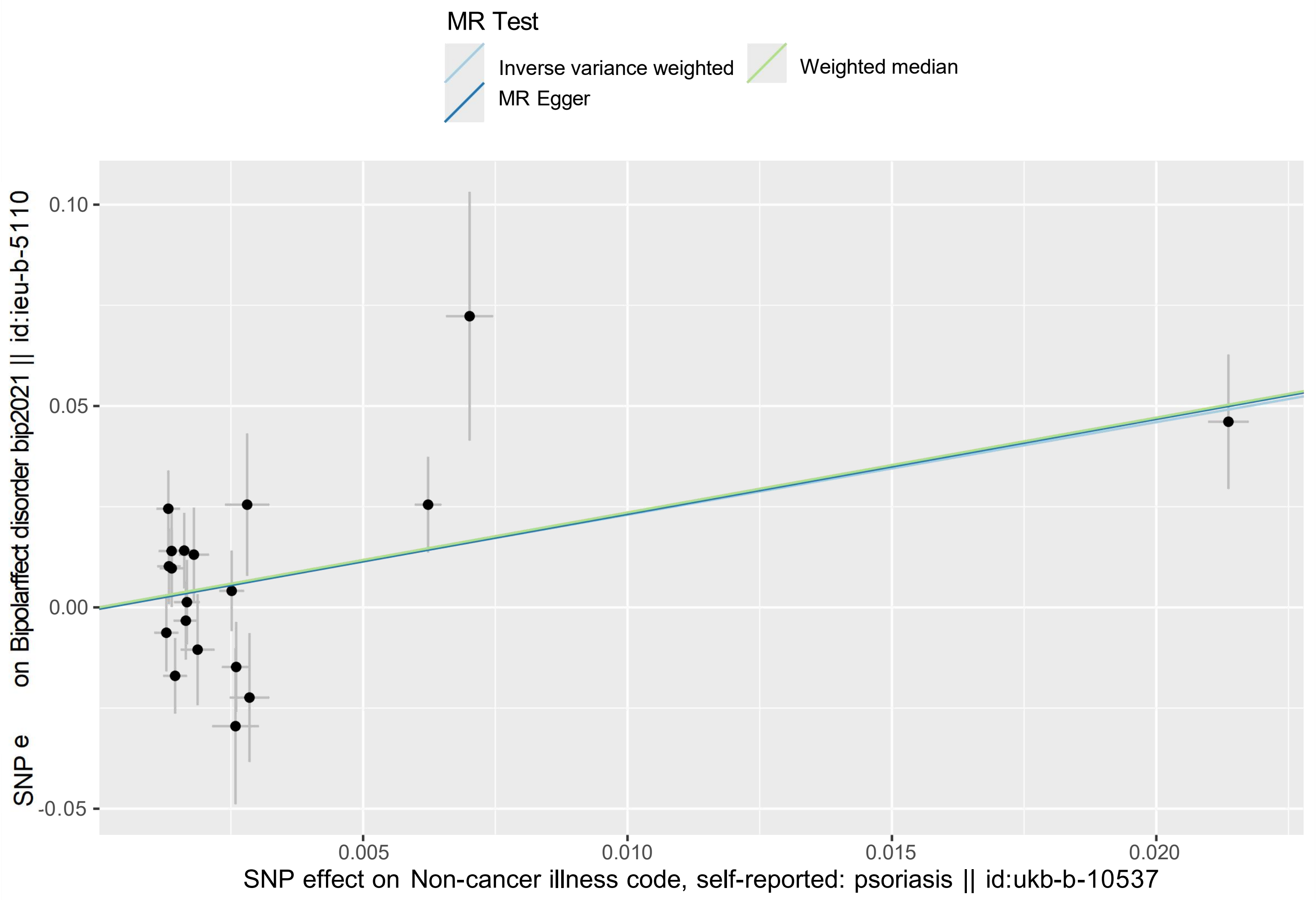

MR Test
Inverse variance weighted
MR Egger
Weighted median
SNP effect on Bipolarffect disorder bip2021 || id:ieu-b-5110
0.10
0.05
0.00
-0.05
0.005
0.010
0.015
0.020
SNP effect on Non-cancer illness code, self-reported: psoriasis || id:ukb-b-10537

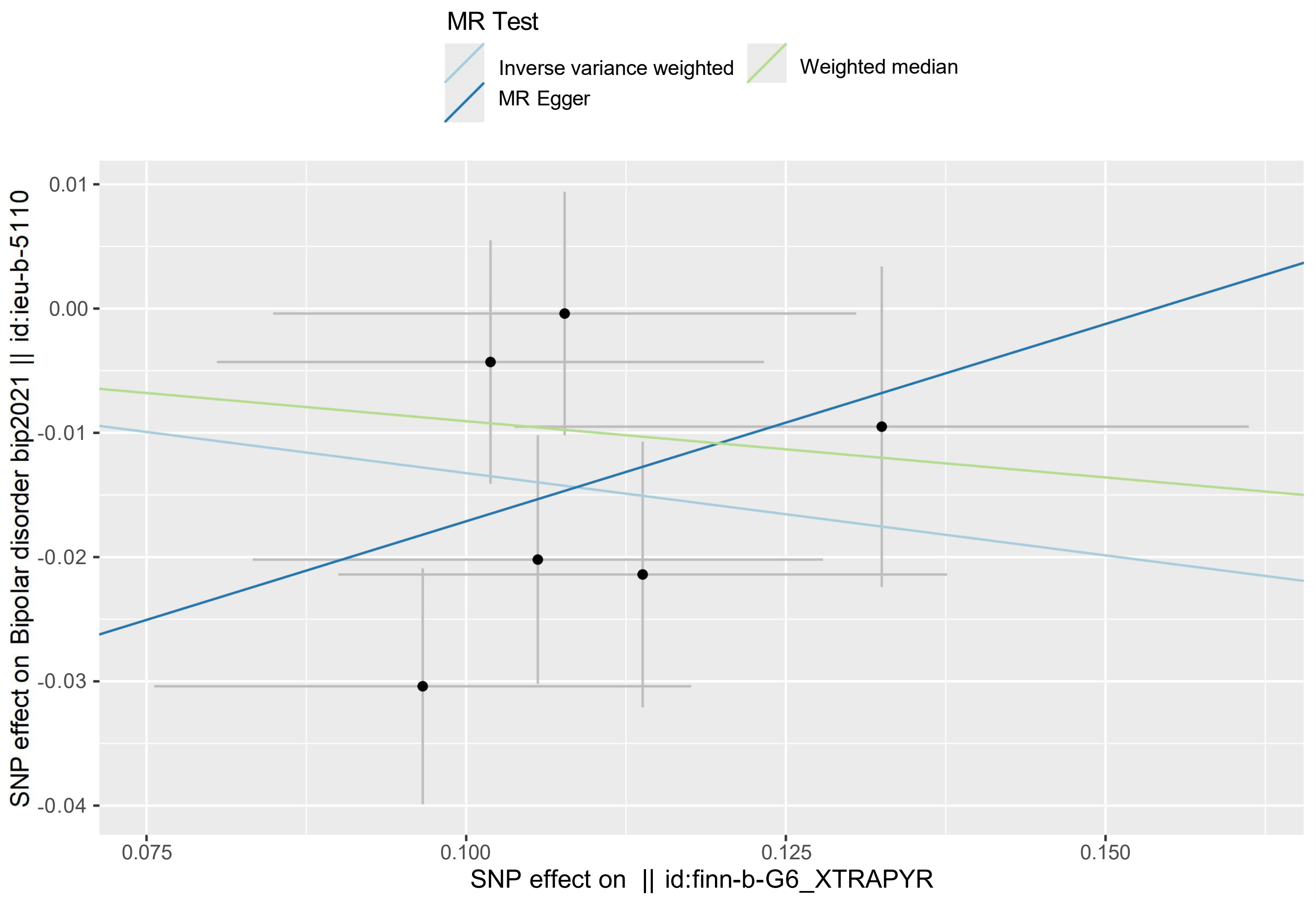
MR Test
Inverse variance weighted
MR Egger
Weighted median
0.01
0.00
-0.01
-0.02
-0.03
-0.04
SNP effect on Bipolar disorder bip2021 || id:ieu-b-5110
0.075
0.100
0.125
0.150
SNP effect on || id:finn-b-G6_XTRAPYR

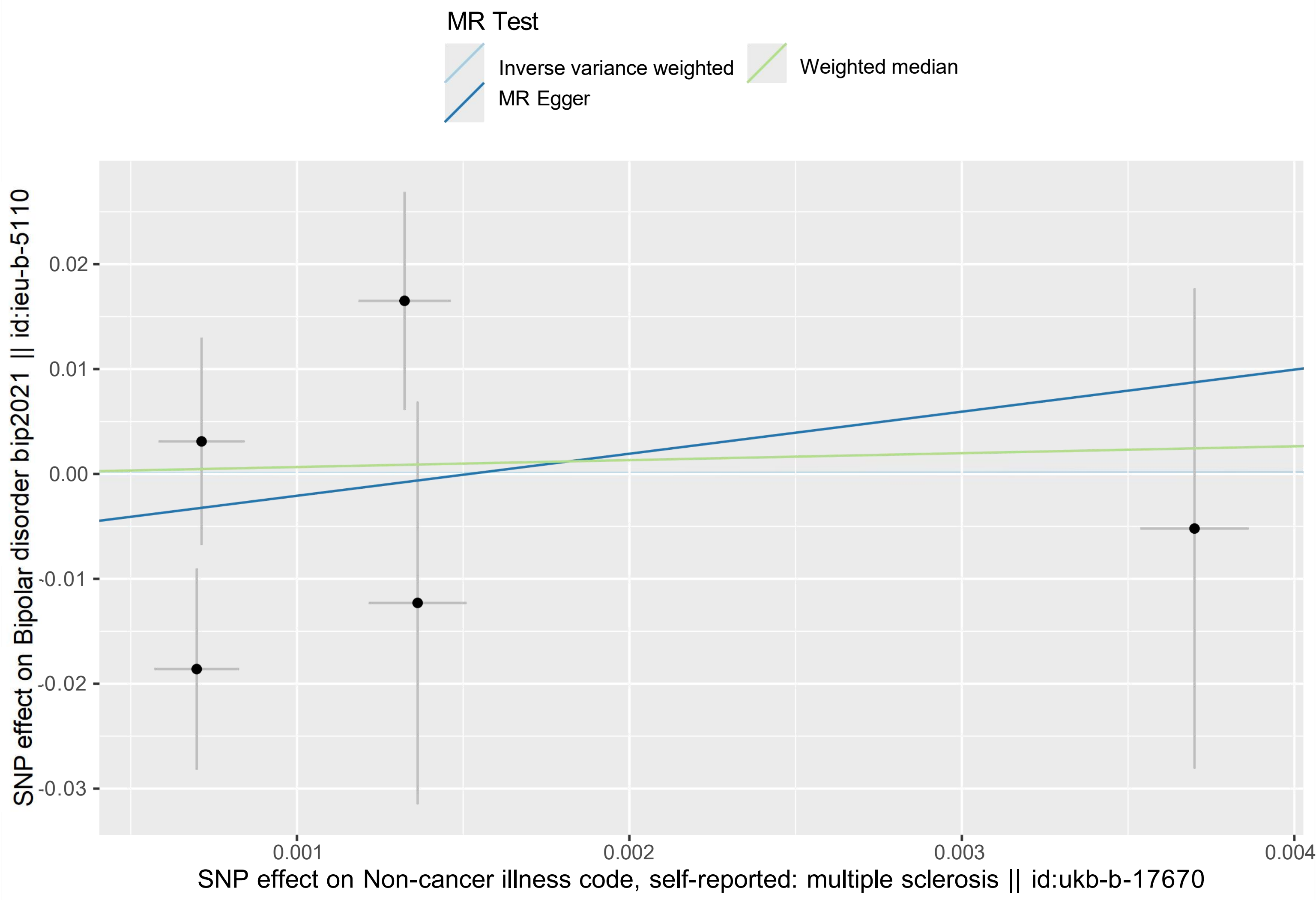

MR Test
Inverse variance weighted
MR Egger
Weighted median
0.02
0.01
0.00
-0.01
-0.02
-0.03
0.001
0.002
0.003
0.004
SNP effect on Bipolar disorder bip2021 || id:ieu-b-5110
SNP effect on Non-cancer illness code, self-reported: multiple sclerosis || id:ukb-b-17670

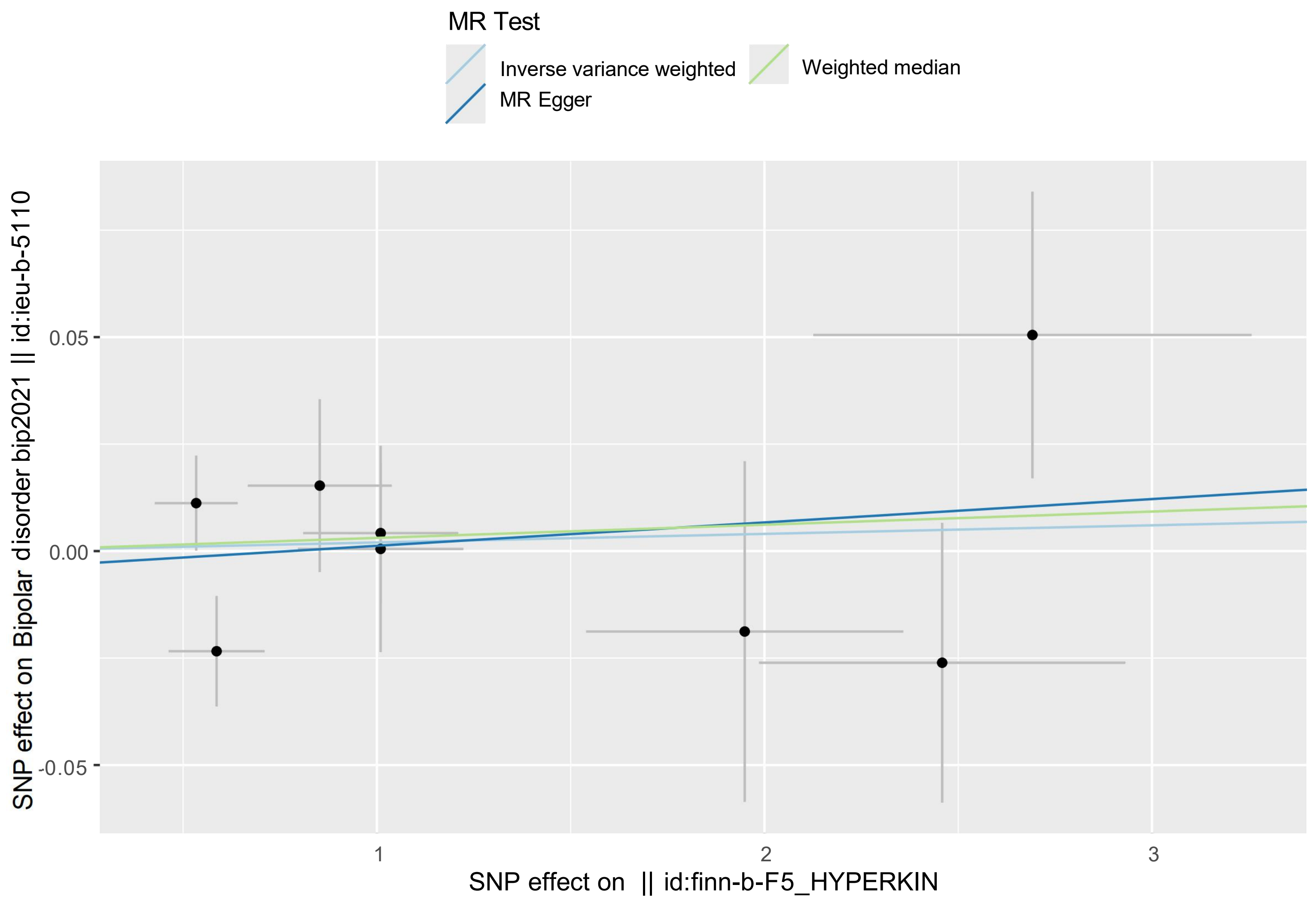

MR Test
Inverse variance weighted
MR Egger
Weighted median
SNP effect on Bipolar disorder bip2021 || id:ieu-b-5110
0.05
0.00
-0.05
1
2
3
SNP effect on || id:finn-b-F5_HYPERKIN

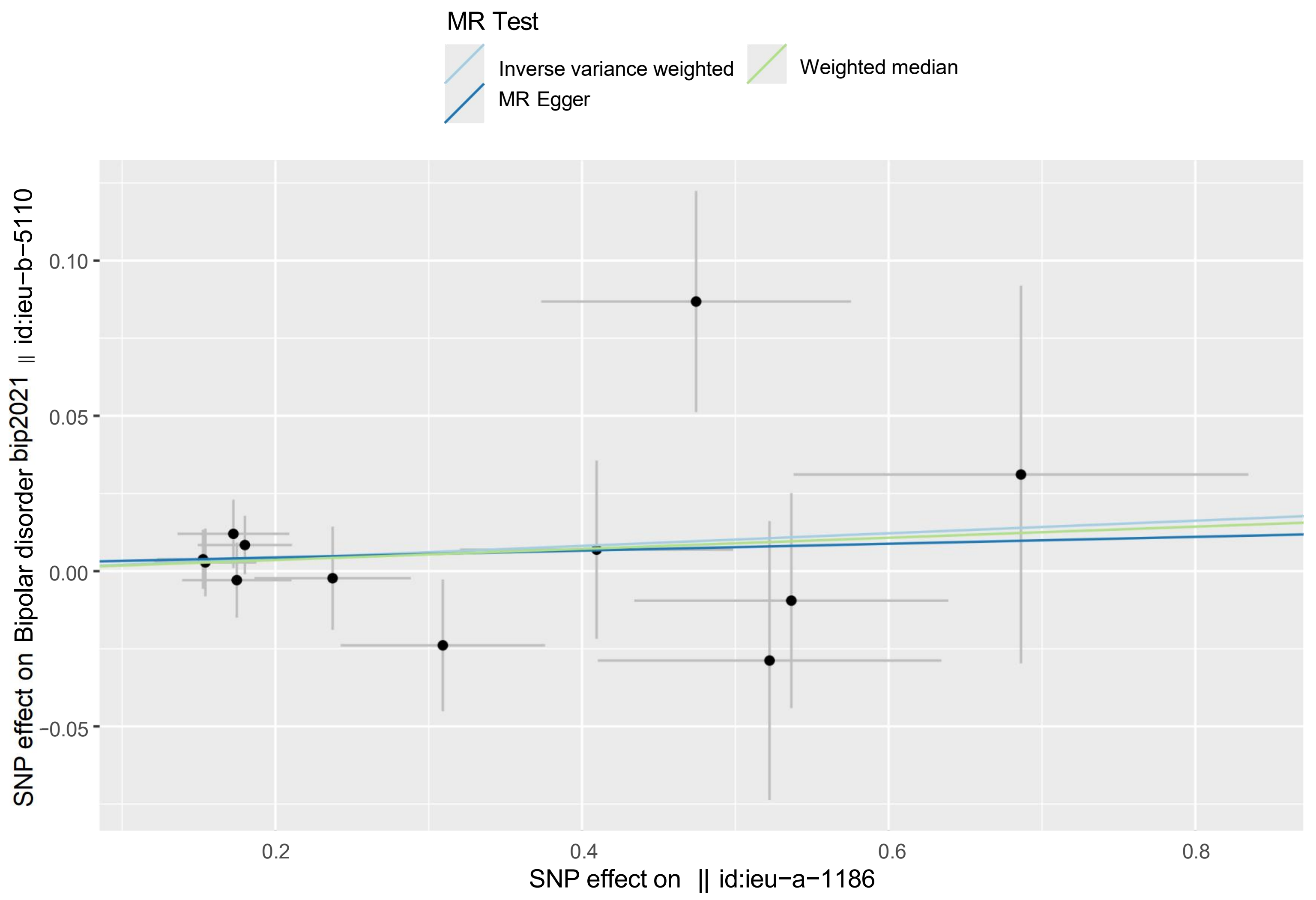
MR Test
Inverse variance weighted
MR Egger
Weighted median
SNP effect on Bipolar disorder bip2021 || id:ieu-b-5110
0.10
0.05
0.00
−0.05
0.2
0.4
0.6
0.8
SNP effect on || id:ieu-a-1186

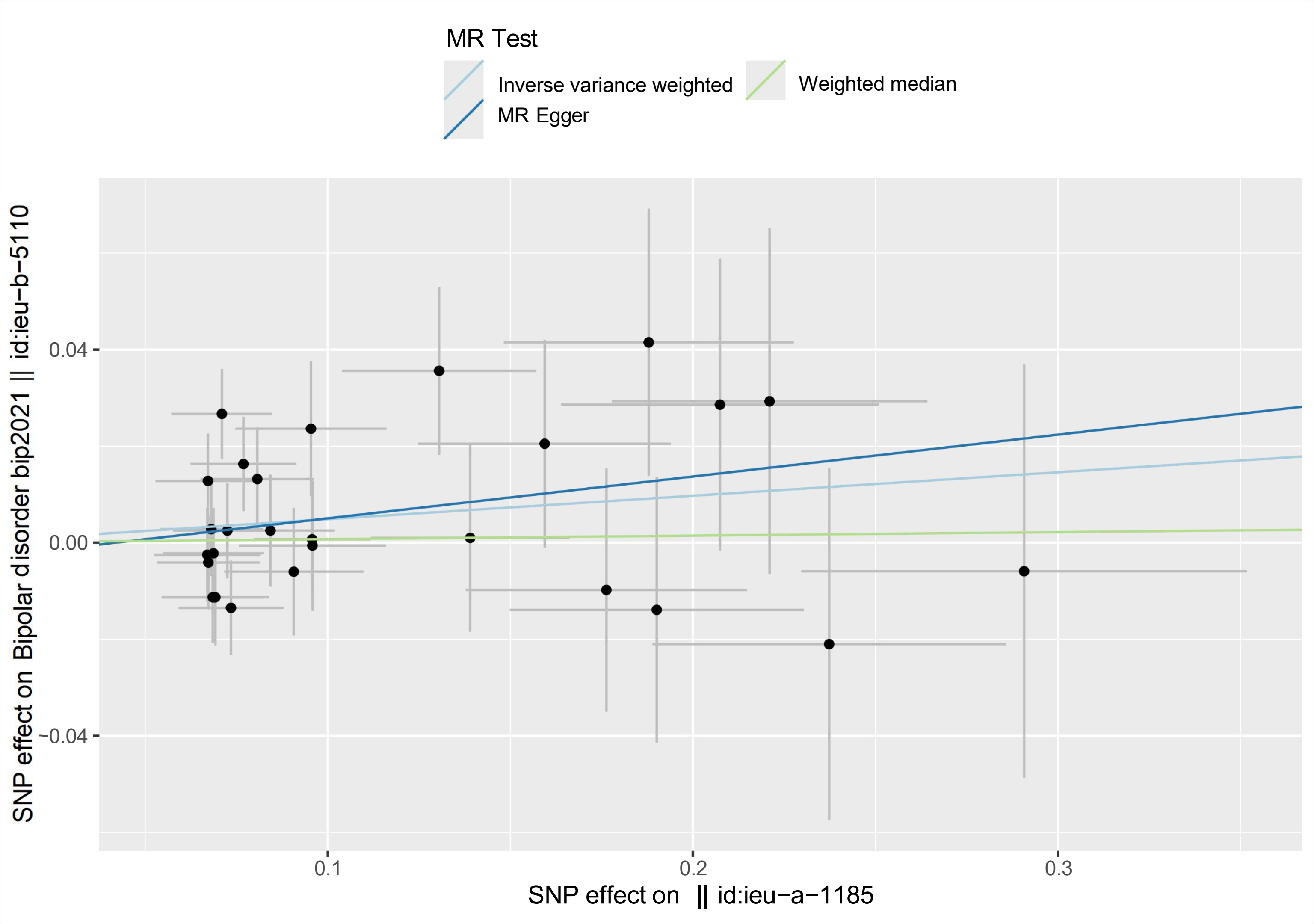

MR Test
Inverse variance weighted
MR Egger
Weighted median
SNP effect on Bipolar disorder bip2021 || id:ieu-b-5110
0.04
0.00
−0.04
0.1
0.2
0.3
SNP effect on || id:ieu−a−1185

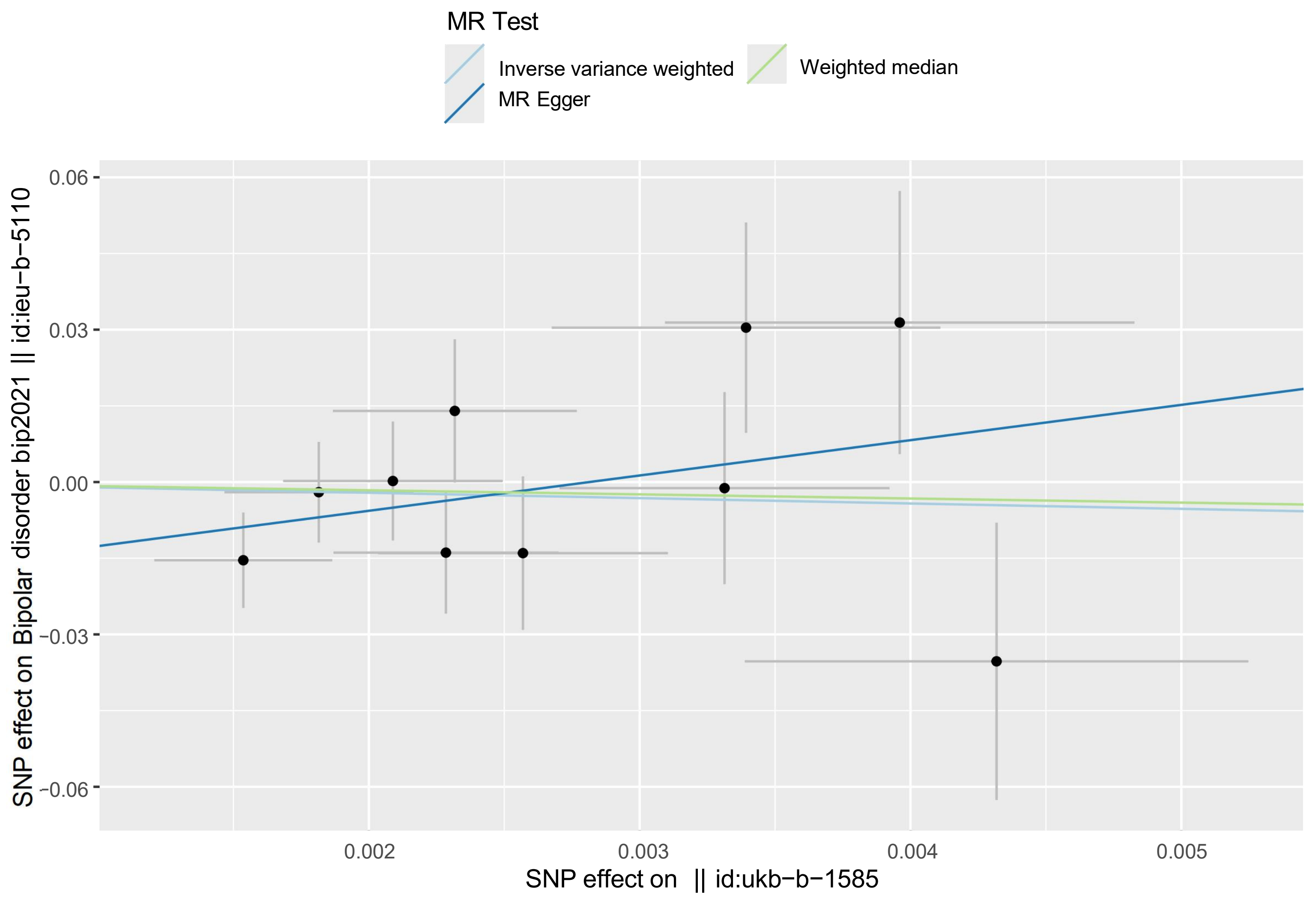

MR Test
Inverse variance weighted
MR Egger
Weighted median
0.06
0.03
0.00
−0.03
−0.06
SNP effect on Bipolar disorder bip2021 || id:ieu−b−5110
0.002
0.003
0.004
0.005
SNP effect on || id:ukb−b−1585

Supplementary file 4

The heterogeneity and horizontal pleiotropy among genetic instruments

Heterogeneity statistics of the MR analysis - bipolar disorders on the risk of sensory disorders.

Outcome	Method	Bipolar disorders		
		Q	***Q_df***	***P* value**
Pain in the limb	IVW	50.56	43	0.20
	MR Egger	50.55	42	0.17
Pruritus	IVW	36.33	41	0.68
	MR Egger	36.27	40	0.64
Hearing impairment	IVW	53.97	40	0.07
	MR Egger	52.34	39	0.08
Tinnitus	IVW	37.19	39	0.55
	MR Egger	37.14	38	0.51
Anosmia	IVW	42.48	41	0.41
	MR Egger	40.67	40	0.44
Small fibre neuropathy	IVW	36.21	41	0.68
	MR Egger	35.00	40	0.69
Psoriasis	IVW	55.92	42	0.07
	MR Egger	54.05	41	0.08

Abbreviations: MR, Mendelian randomization; IVW, inverse-variance weighted.

Heterogeneity statistics of the MR analysis - bipolar disorders on the risk of motor and behavioral disorders.

Outcome	Method	Bipolar disorders		
		Q	***Q_df***	***P* value**
Extrapyramidal and movement disorders	IVW	41.51	41	0.45
	MR Egger	41.26	40	0.42
Multiple Sclerosis	IVW	38.64	34	0.27
	MR Egger	36.81	33	0.30
Hyperkinetic disorders	IVW	28.59	41	0.93
	MR Egger	28.57	40	0.91
Anorexia nervosa	IVW	52.50	43	0.15
	MR Egger	52.34	42	0.13
Autism spectrum disorder	IVW	53.28	38	0.051
	MR Egger	51.77	37	0.054
Psychoactive substance abuse	IVW	37.32	43	0.72
	MR Egger	36.75	42	0.70

Abbreviations: MR, Mendelian randomization; IVW, inverse-variance weighted.

Heterogeneity statistics of the MR analysis - sensory disorders on the risk of bipolar disorders.

Exposure	Method	Bipolar disorders		
		Q	*Q_df*	*P* value
Pain in the limb	IVW	15.23	7	0.14
	MR Egger	9.68	6	0.16
Pruritus	IVW	5.13	5	0.40
	MR Egger	3.17	4	0.53
Hearing impairment	IVW	34.66	26	0.12
	MR Egger	33.42	25	0.12
Tinnitus	IVW	19.44	14	0.15
	MR Egger	19.33	13	0.11
Anosmia	IVW	8.81	10	0.55
	MR Egger	8.73	9	0.46
Small fibre neuropathy	IVW	4.71	5	0.45
	MR Egger	3.46	4	0.48
Psoriasis	IVW	31.84	18	0.02
	MR Egger	31.83	17	0.02

Abbreviations: MR, Mendelian randomization; IVW, inverse-variance weighted.

Heterogeneity statistics of the MR analysis - motor and behavioral disorders on the risk of bipolar disorders.

Exposure	Method	Bipolar disorders		
		Q	*Q_df*	*P* value
Extrapyramidal and movement disorders	IVW	7.44	5	0.19
	MR Egger	6.26	4	0.18
Multiple Sclerosis	IVW	6.83	4	0.14
	MR Egger	6.46	3	0.09
Hyperkinetic disorders	IVW	7.98	7	0.33
	MR Egger	7.85	6	0.25
Anorexia nervosa	IVW	9.25	11	0.60
	MR Egger	9.21	10	0.51
Autism spectrum disorder	IVW	28.82	26	0.32
	MR Egger	28.54	25	0.28
Psychoactive substance abuse	IVW	10.89	9	0.28
	MR Egger	9.16	8	0.33

Abbreviations: MR, Mendelian randomization; IVW, inverse-variance weighted.

Horizontal pleiotropy of the exposure SNPs in the MR analysis - bipolar disorders on the risk of sensory, motor and behavioral disorders.

Outcome	Bipolar disorders		
	**ERI**	***SE***	**Directionality *P* value**
Pain in the limb	-2.31E-05	0.0003	0.93
Pruritus	0.009	0.04	0.80
Hearing impairment	-0.003	0.003	0.28
Tinnitus	0.0002	0.001	0.83
Anosmia	-0.09	0.07	0.19
Small fibre neuropathy	0.09	0.08	0.28
Psoriasis	0.0003	0.0003	0.24
Extrapyramidal and movement disorders	-0.01	0.02	0.63
Multiple Sclerosis	-0.0003	0.0002	0.21
Hyperkinetic disorders	0.01	0.09	0.87
Anorexia nervosa	0.01	0.03	0.73
Autism spectrum disorder	0.02	0.02	0.31
Psychoactive substance abuse	0.0002	0.0003	0.46

Abbreviations: SNP, single nucleotide polymorphism; MR, Mendelian randomization; ERI, egger regression intercept; SE, standard error.

Horizontal pleiotropy of the exposure SNPs in the MR analysis - sensory, motor and behavioral disorders on the risk of bipolar disorders.

Exposure	Bipolar disorders		
	**ERI**	***SE***	**Directionality *P* value**
Pain in the limb	-0.02	0,02	0.48
Pruritus	0.01	0.01	0.23
Hearing impairment	-0.009	0.01	0.35
Tinnitus	-0.004	0.01	0.79
Anosmia	-0.002	0.009	0.79
Small fibre neuropathy	0.01	0.01	0.33
Psoriasis	-0.0004	0.004	0.93
Extrapyramidal and movement disorders	-0.05	0.06	0.43
Multiple Sclerosis	-0.006	0.01	0.70
Hyperkinetic disorders	-0.004	0.01	0.77
Anorexia nervosa	0.002	0.01	0.84
Autism spectrum disorder	-0.004	0.007	0.63
Psychoactive substance abuse	-0.02	0.02	0.25

Abbreviations: SNP, single nucleotide polymorphism; MR, Mendelian randomization; ERI, egger regression intercept; SE, standard error.

Supplementary file 5

Results of Funnel Plot Analysis for Mendelian Randomization

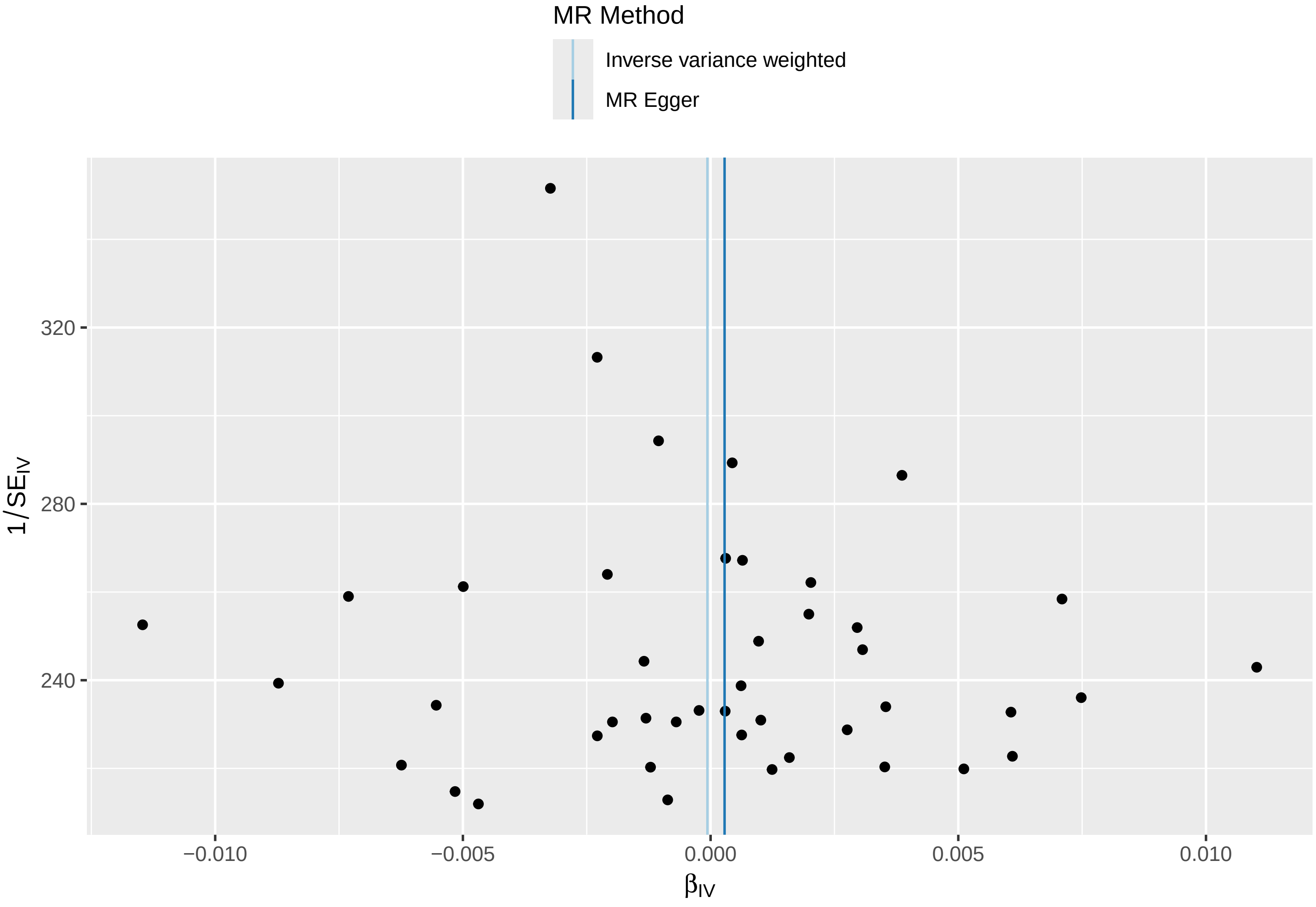

MR Method
Inverse variance weighted
MR Egger
320
280
240
1/SE_IV
−0.010
−0.005
0.000
0.005
0.010
β_IV

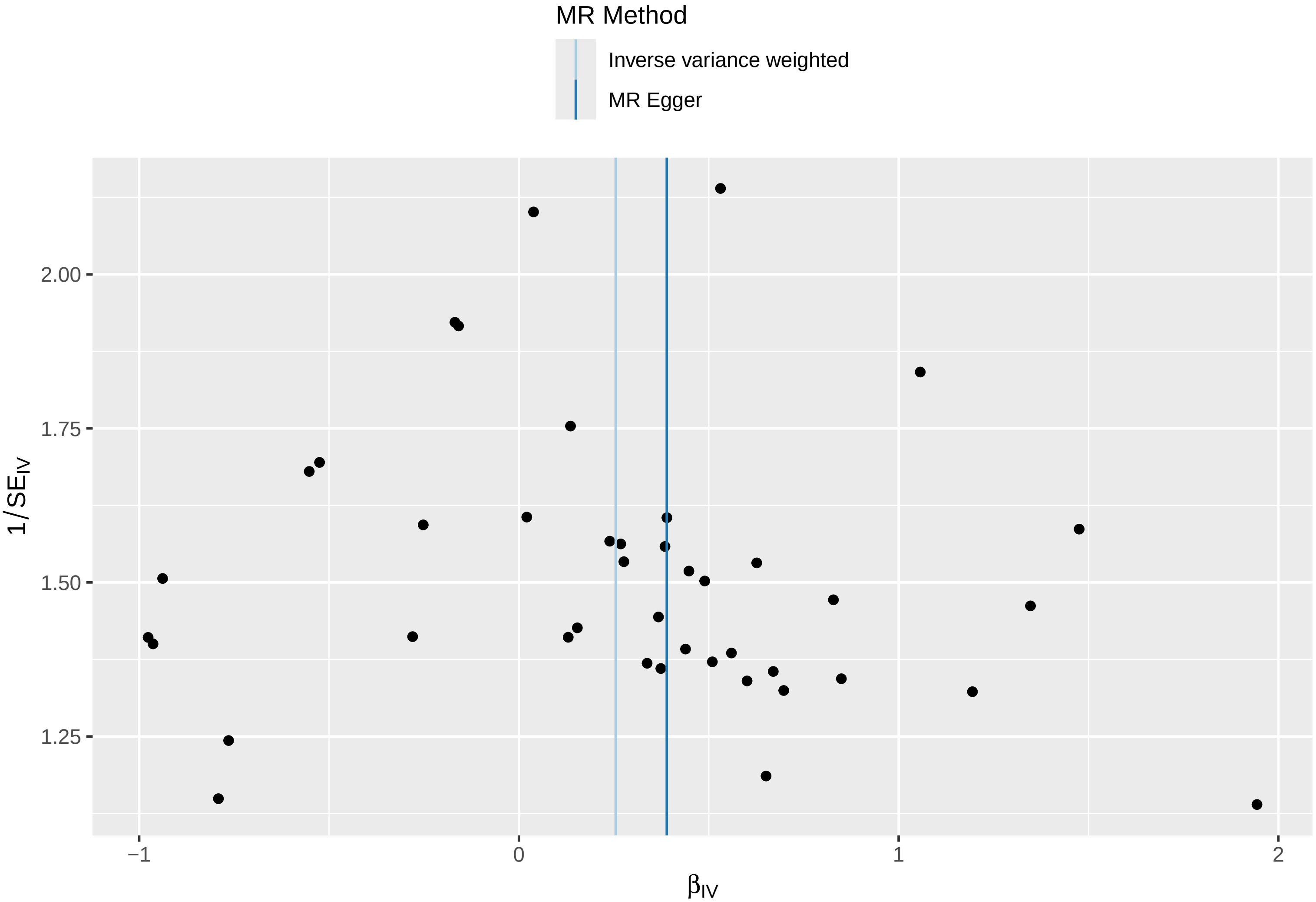

MR Method
Inverse variance weighted
MR Egger
2.00
1.75
1.50
1.25
$1/SE_{IV}$
−1
0
1
2
β_{IV}

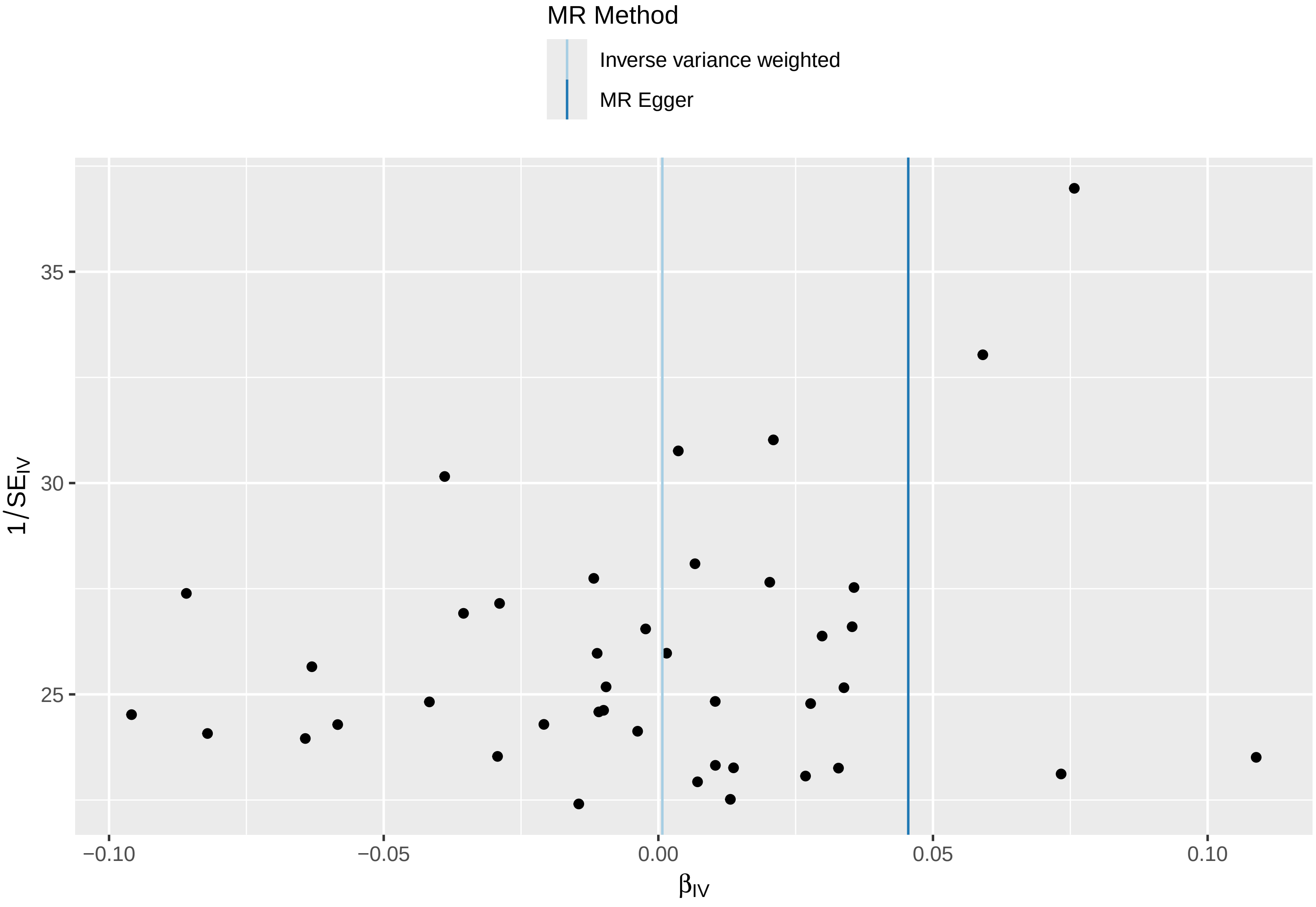
MR Method
Inverse variance weighted
MR Egger
35
30
25
1/SE$_{IV}$
−0.10
−0.05
0.00
0.05
0.10
β$_{IV}$

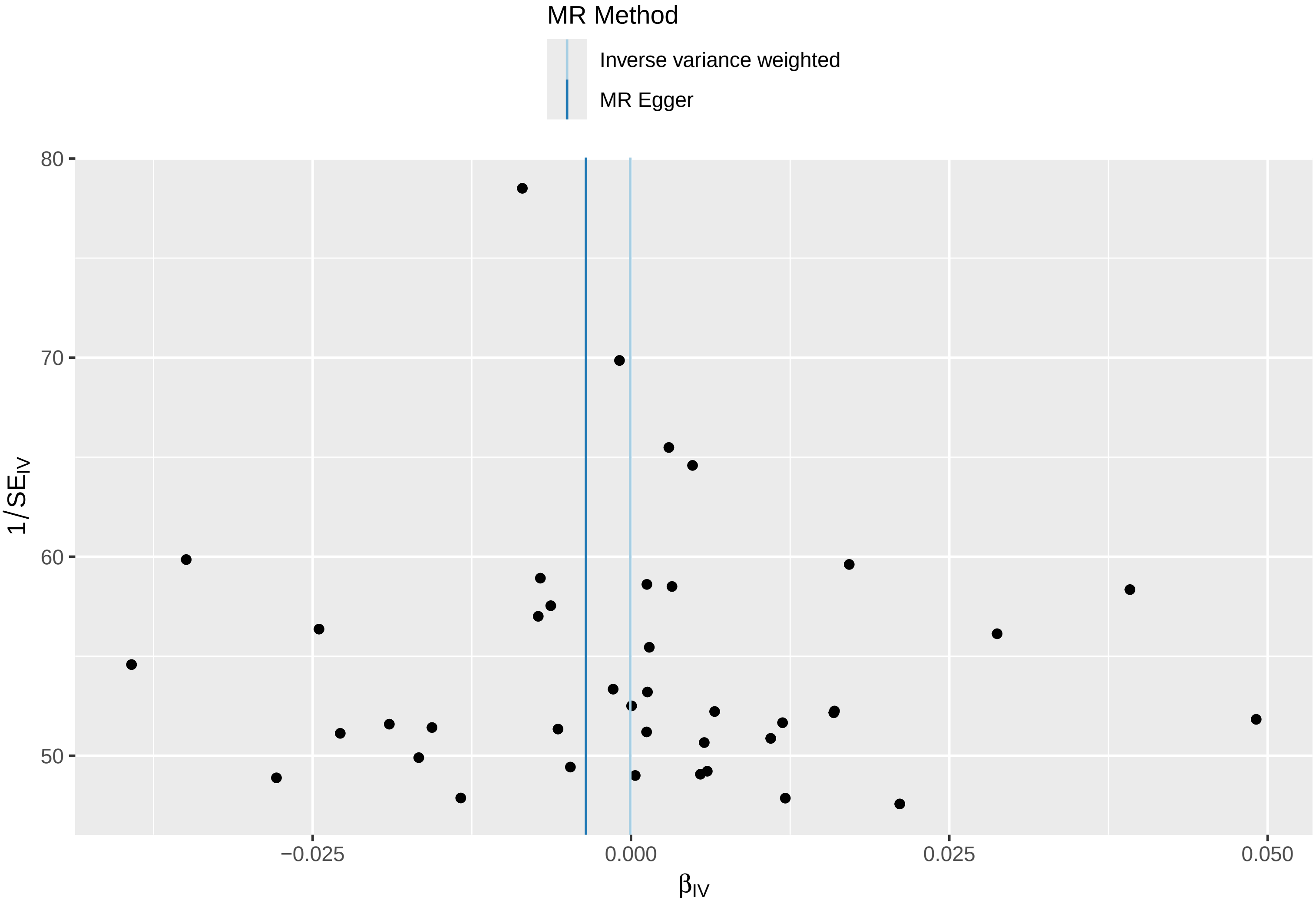

MR Method
Inverse variance weighted
MR Egger
80
70
60
50
$1/SE_{IV}$
−0.025
0.000
0.025
0.050
β_{IV}

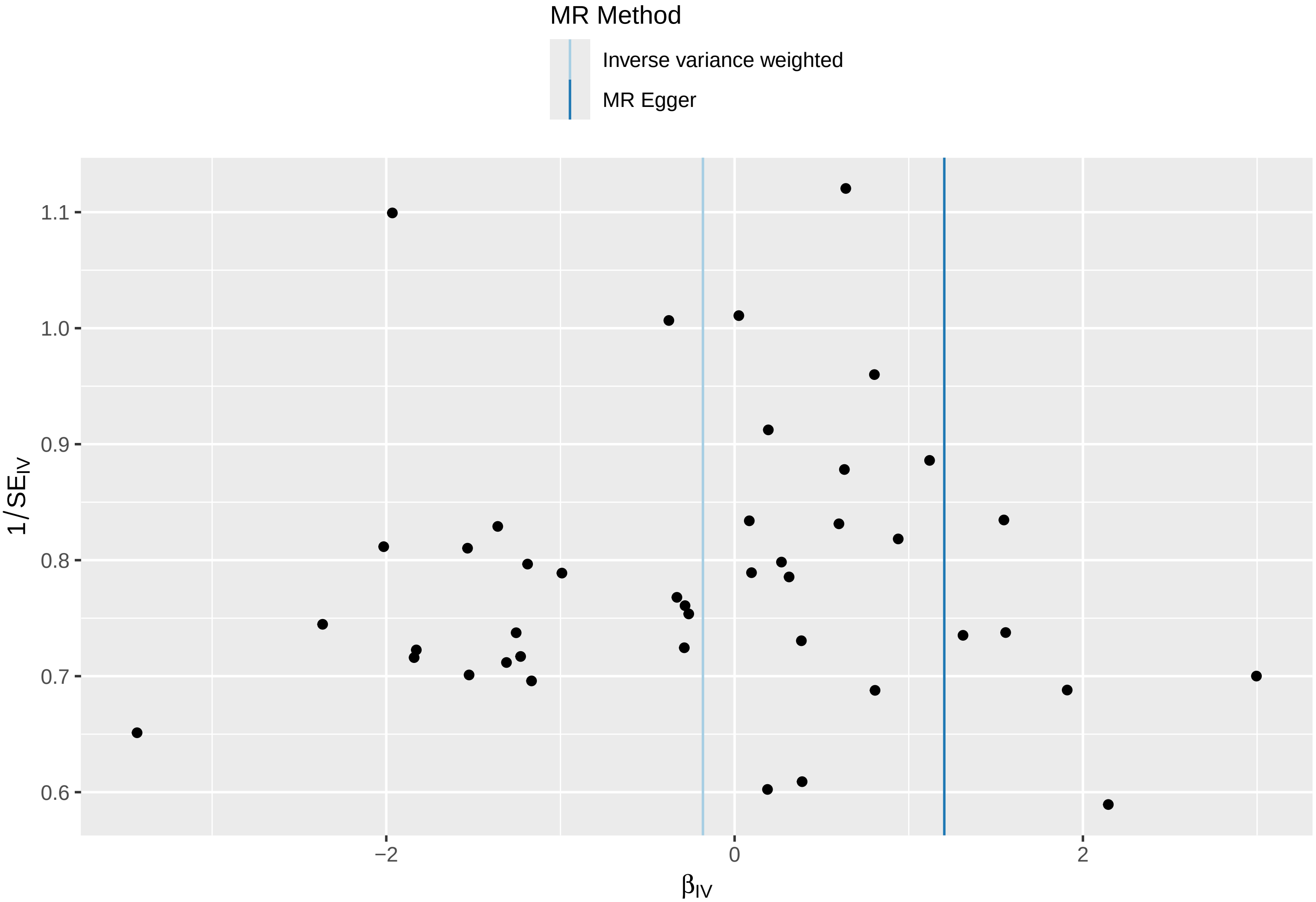
MR Method
Inverse variance weighted
MR Egger
1.1
1.0
0.9
0.8
0.7
0.6
−2
0
2
$1/SE_{IV}$
β_{IV}

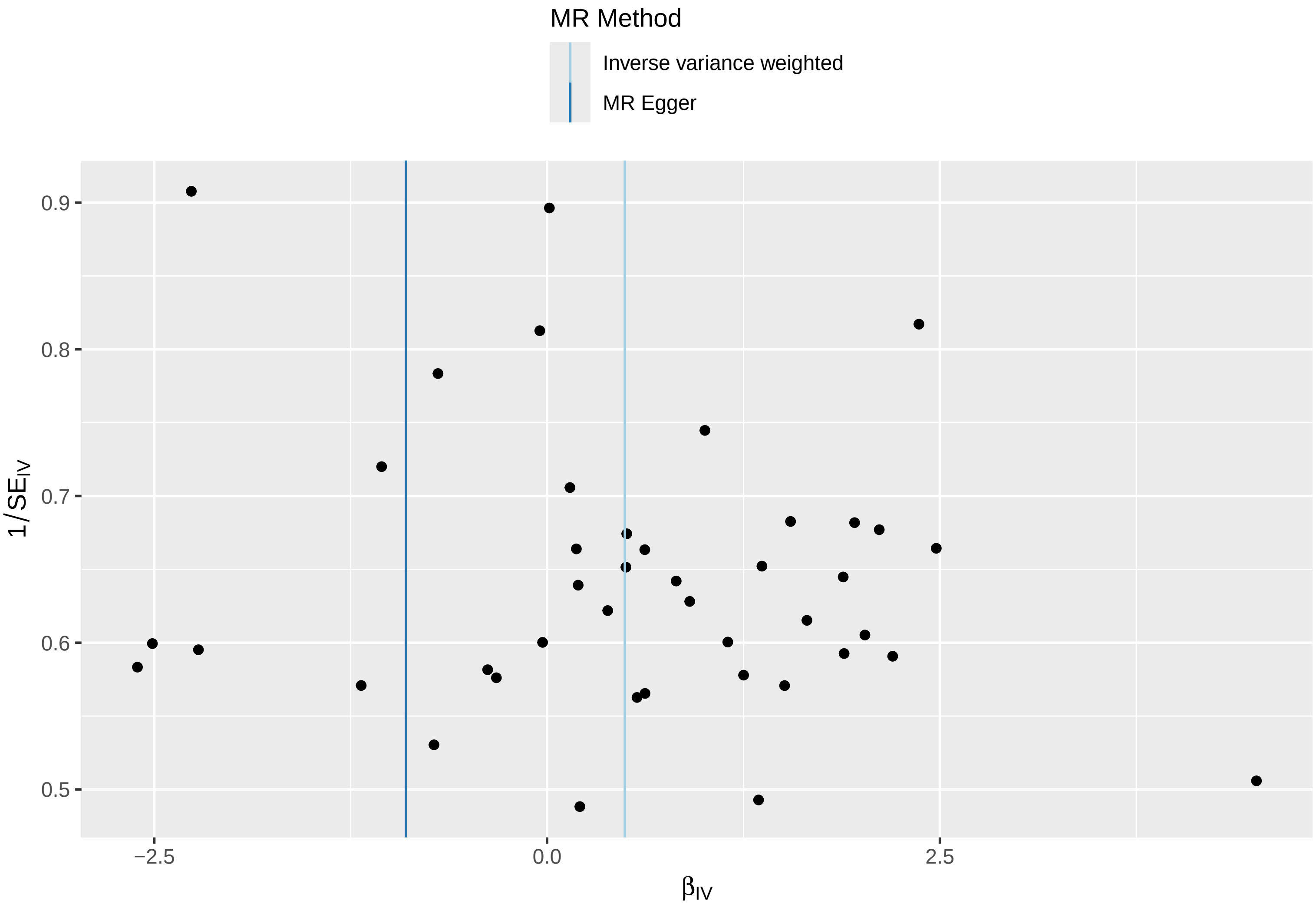

MR Method
Inverse variance weighted
MR Egger
0.9
0.8
0.7
0.6
0.5
1/SE_IV
−2.5
0.0
2.5
β_IV

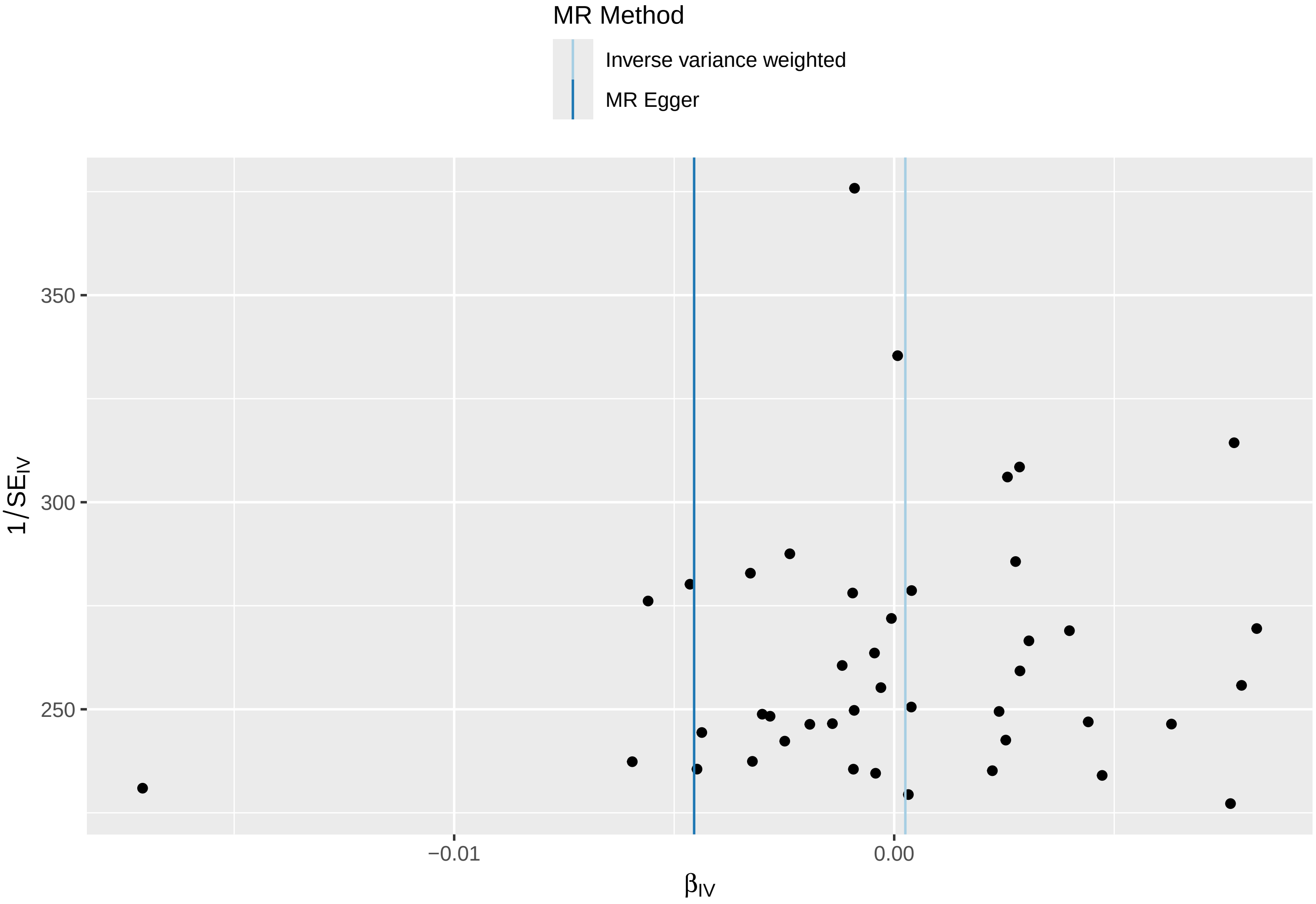
MR Method
Inverse variance weighted
MR Egger
350
300
250
1/SE_IV
−0.01
0.00
β_IV

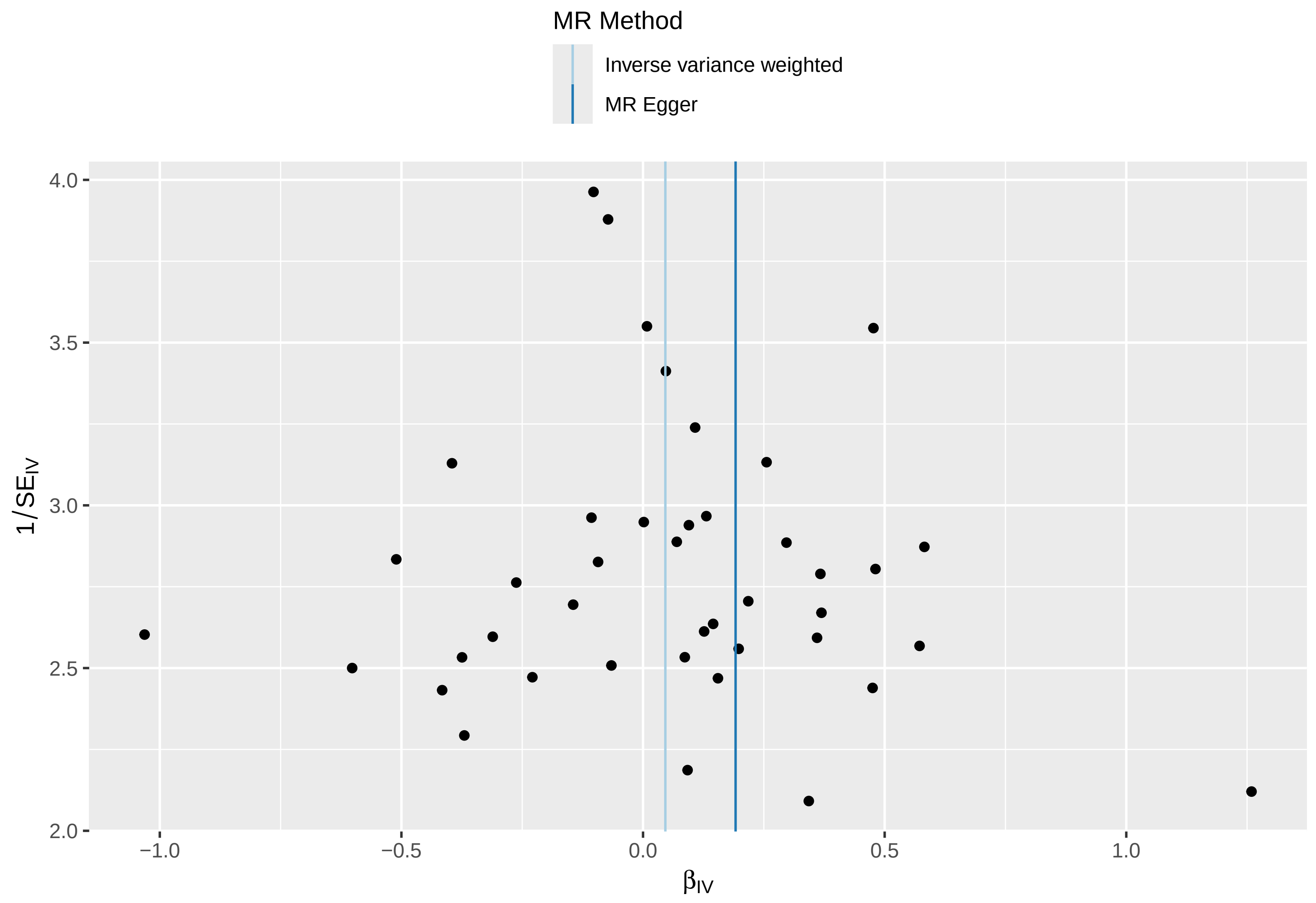

MR Method
Inverse variance weighted
MR Egger
4.0
3.5
3.0
2.5
2.0
$1/SE_{IV}$
−1.0
−0.5
0.0
0.5
1.0
β_{IV}

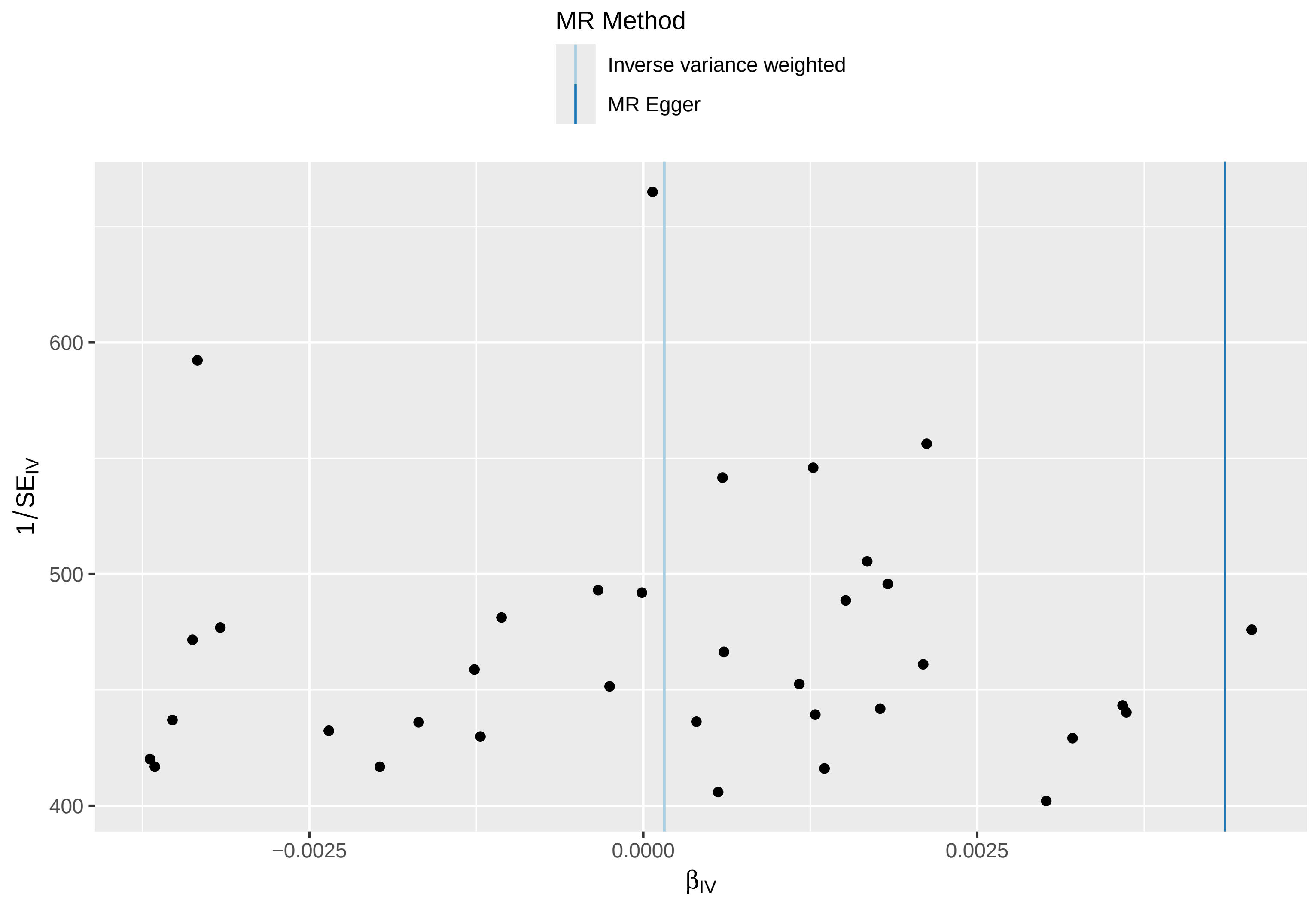
MR Method
Inverse variance weighted
MR Egger
600
500
400
1/SE_IV
−0.0025
0.0000
0.0025
β_IV

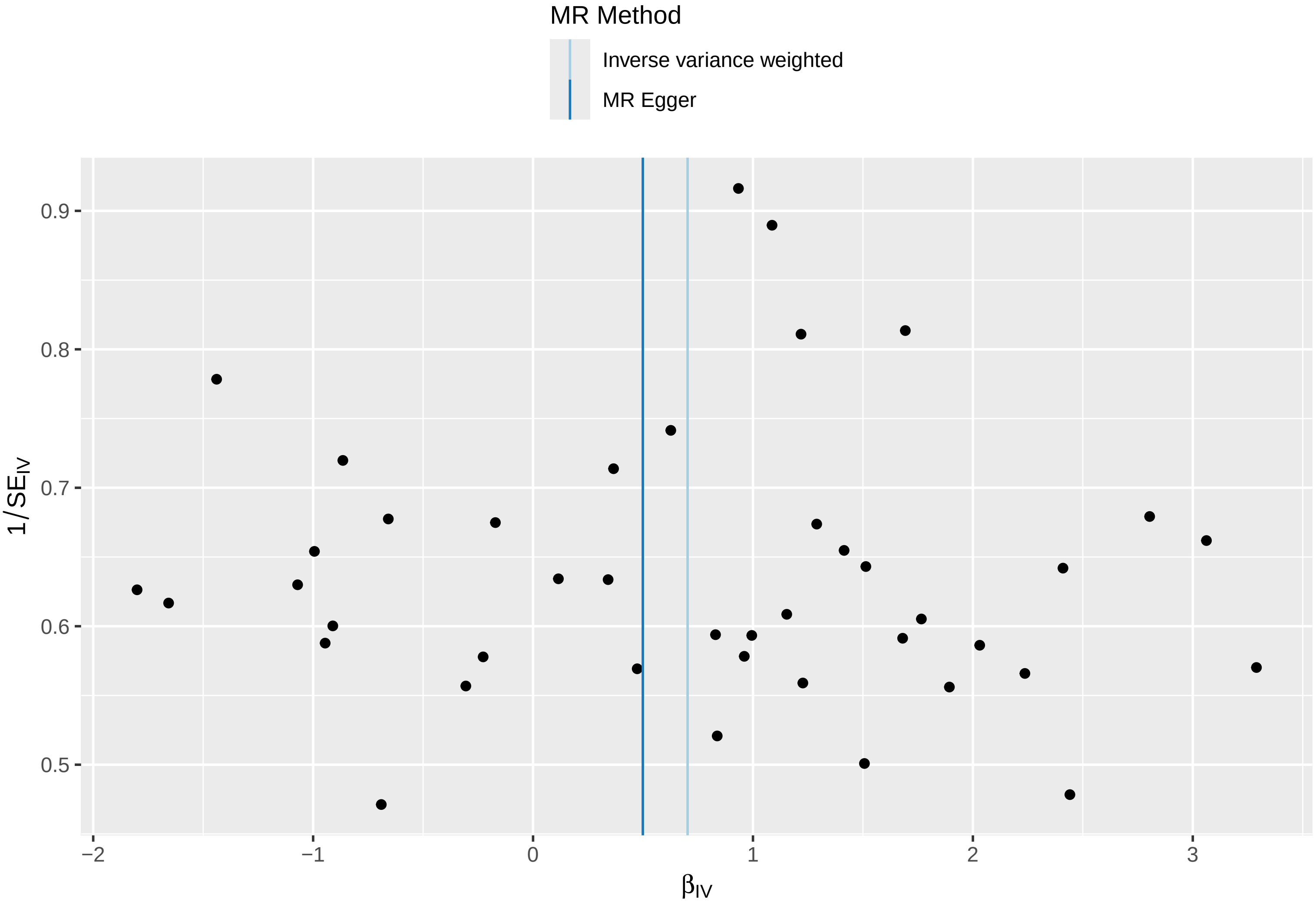
MR Method
Inverse variance weighted
MR Egger
0.9
0.8
0.7
0.6
0.5
1/SE_IV
−2
−1
0
1
2
3
β_IV

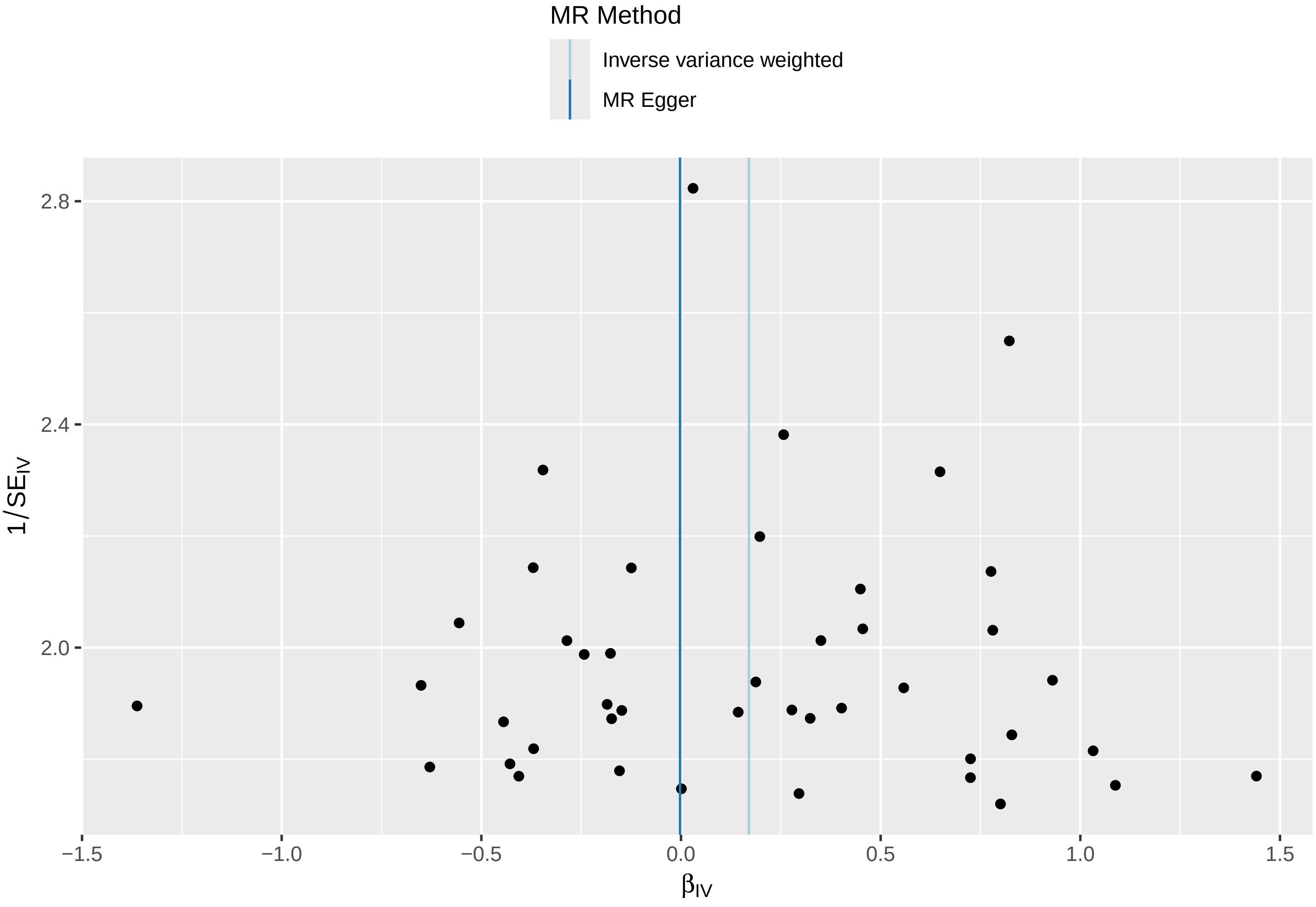

MR Method
Inverse variance weighted
MR Egger
2.8
2.4
2.0
$1/SE_{IV}$
−1.5
−1.0
−0.5
0.0
0.5
1.0
1.5
β_{IV}

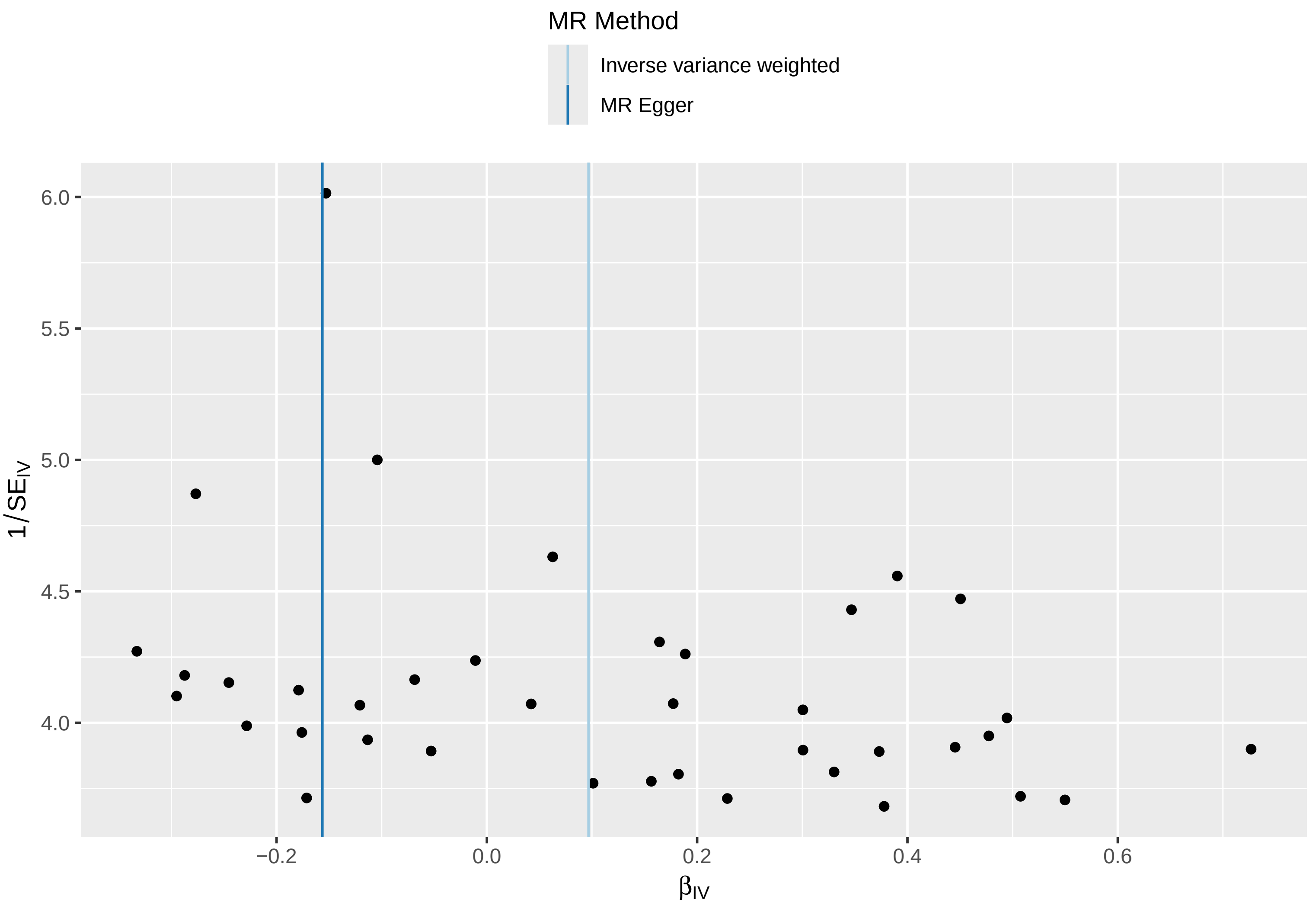
MR Method
Inverse variance weighted
MR Egger
6.0
5.5
5.0
4.5
4.0
1/SE_IV
−0.2
0.0
0.2
0.4
0.6
β_IV

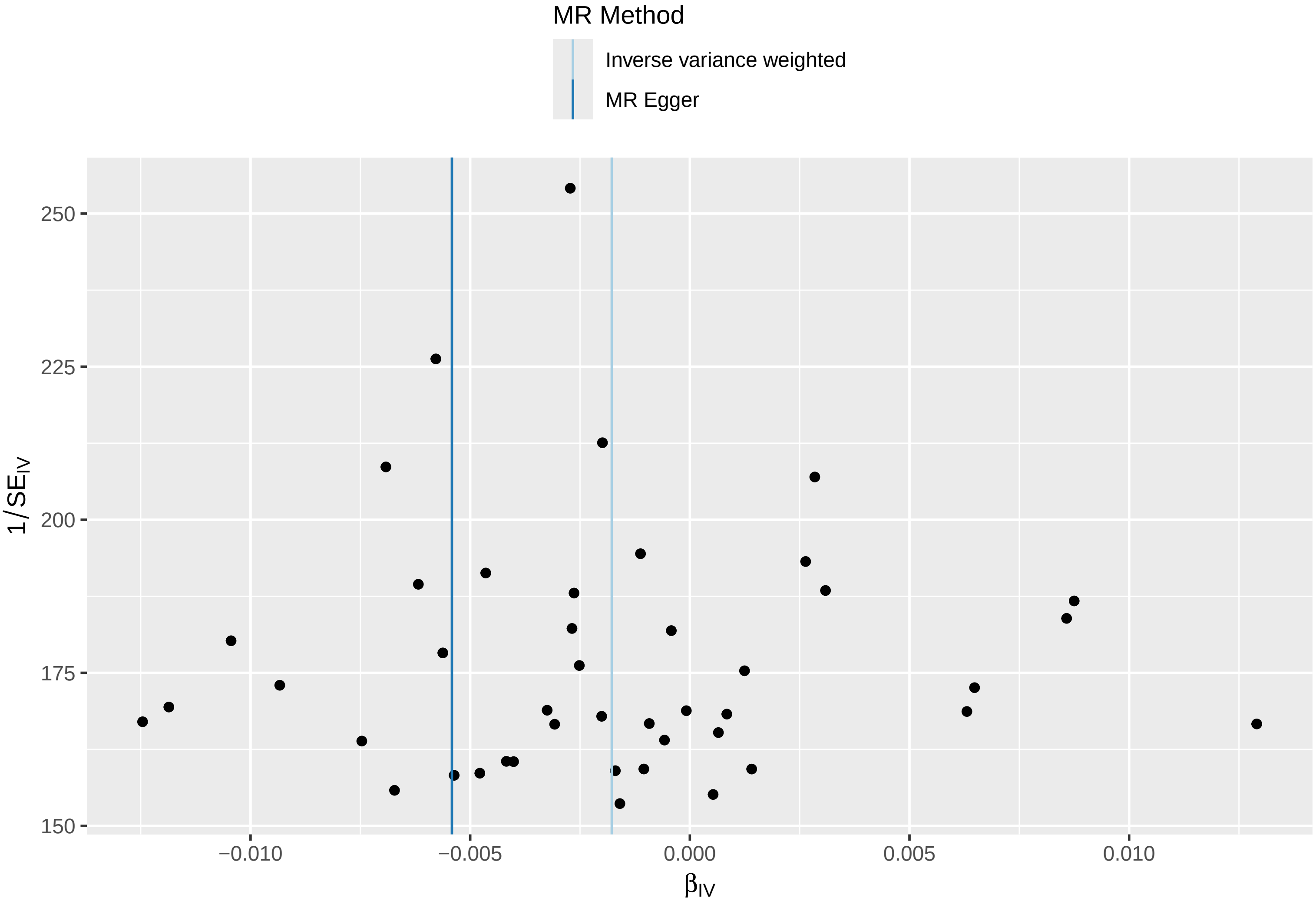
MR Method
Inverse variance weighted
MR Egger
250
225
200
175
150
−0.010
−0.005
0.000
0.005
0.010
β_{IV}
$1/SE_{IV}$

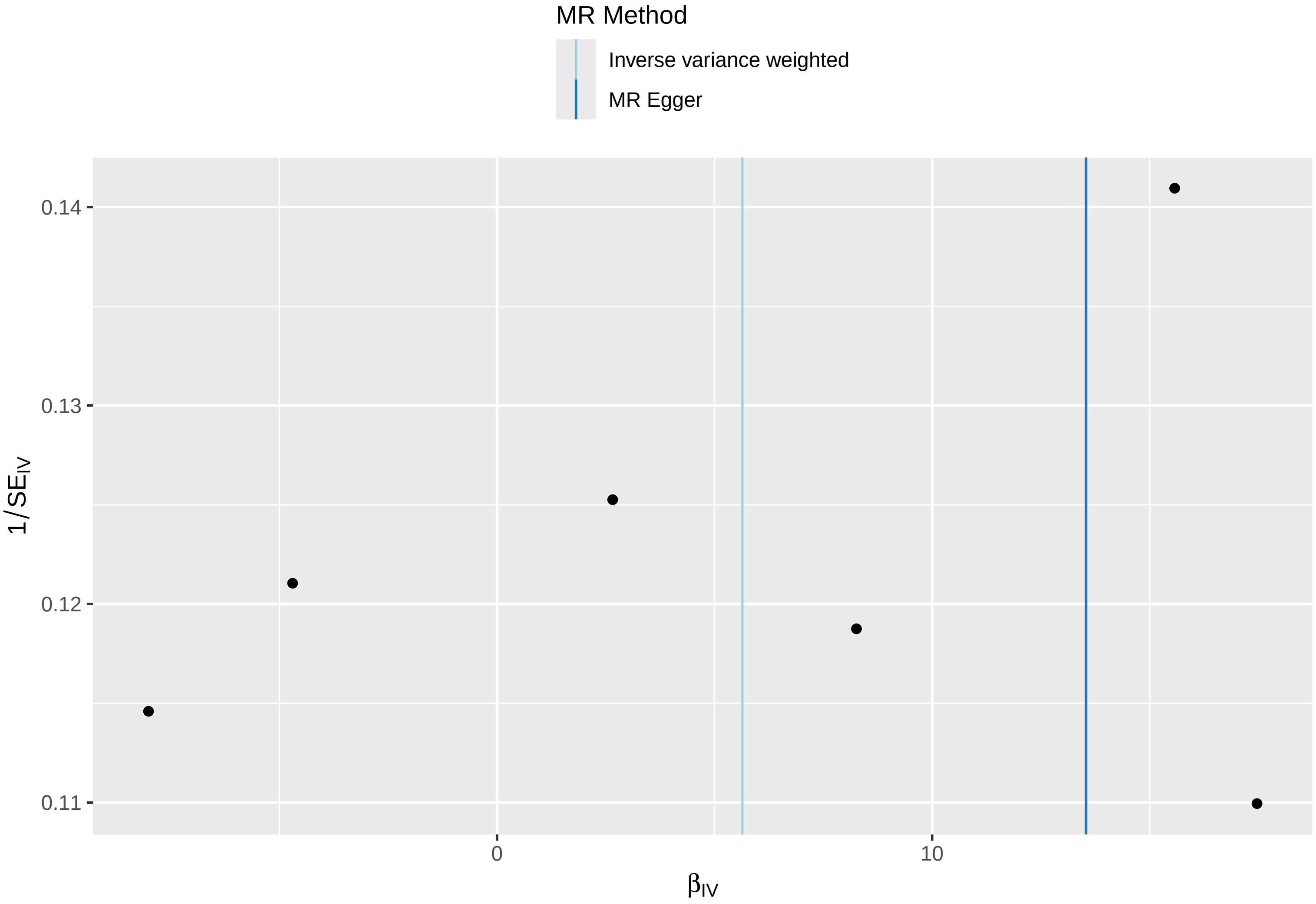
MR Method
Inverse variance weighted
MR Egger
0.14
0.13
0.12
0.11
0
10
$1/SE_{IV}$
β_{IV}

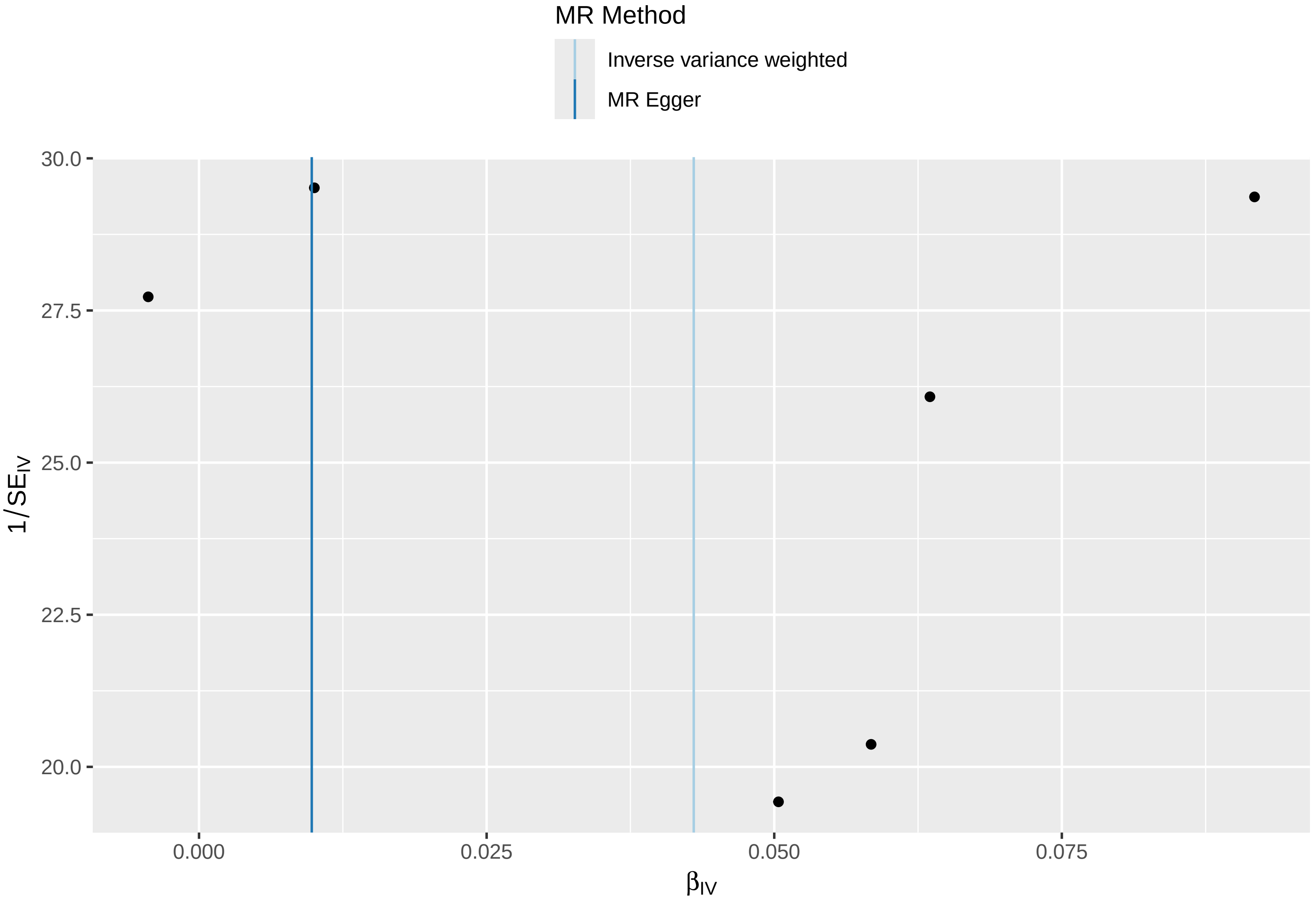
MR Method
Inverse variance weighted
MR Egger
30.0
27.5
25.0
22.5
20.0
1/SE$_{IV}$
0.000
0.025
0.050
0.075
β$_{IV}$

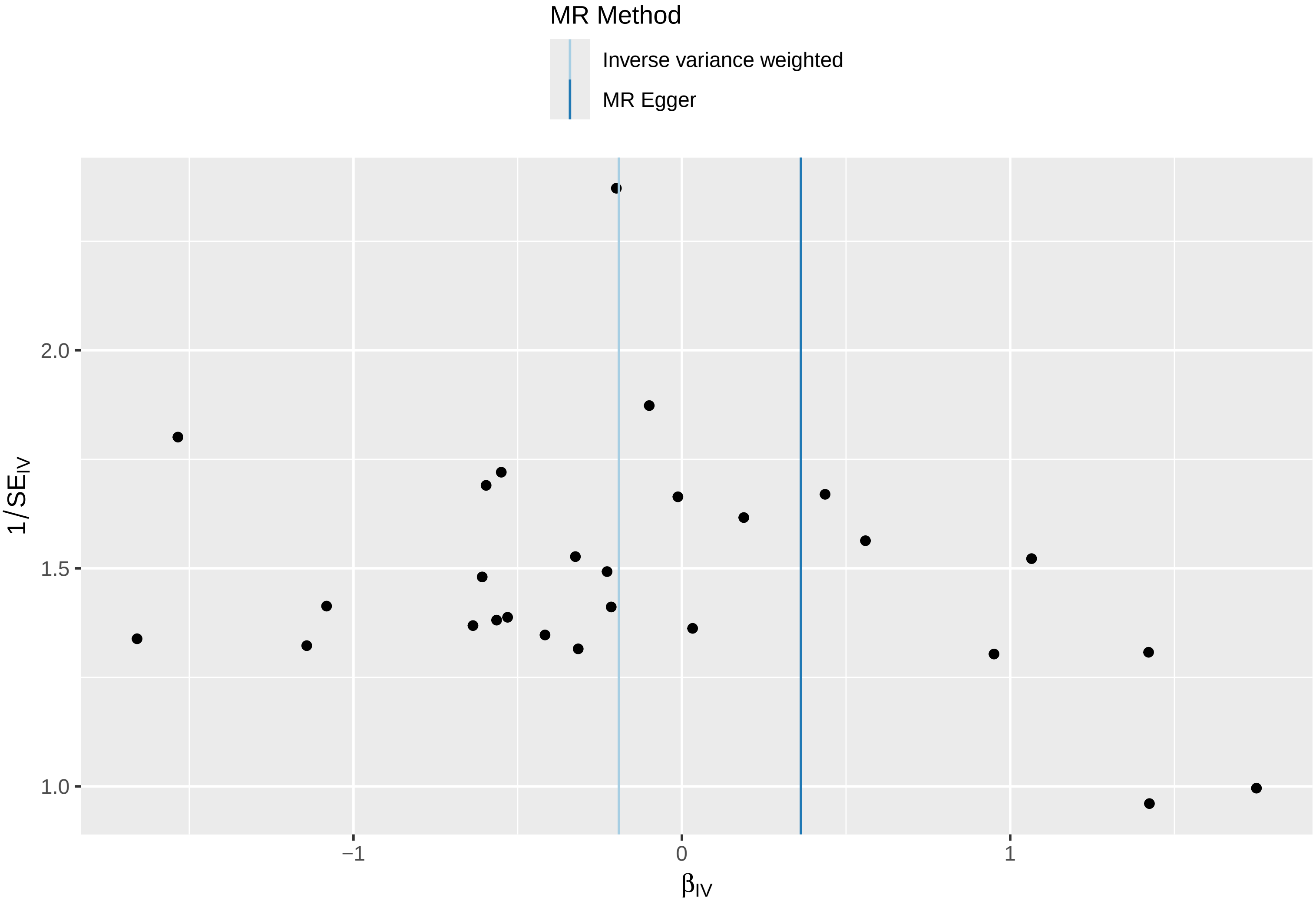

MR Method
Inverse variance weighted
MR Egger
2.0
1.5
1.0
1/SE_IV
−1
0
1
β_IV

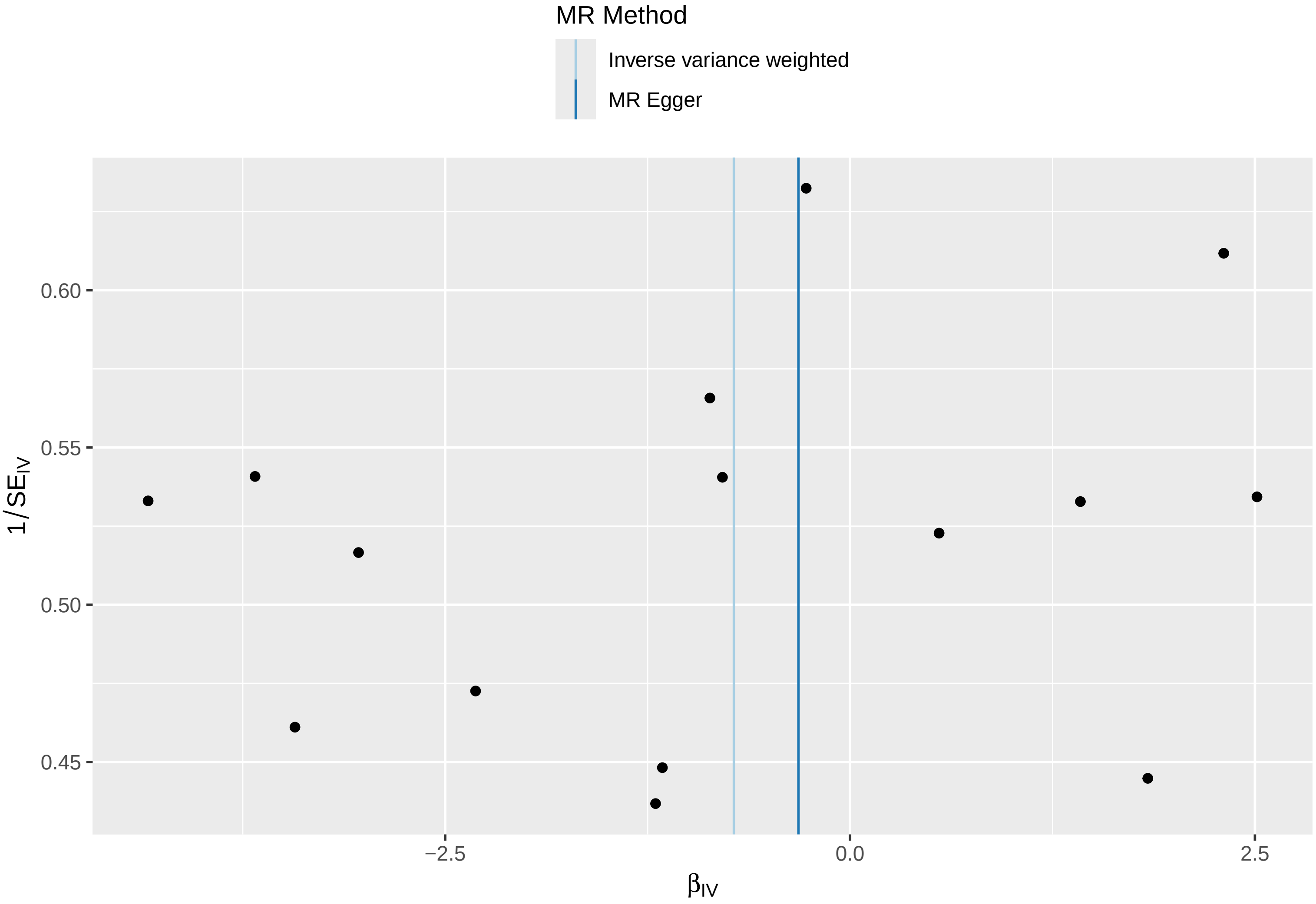

MR Method
Inverse variance weighted
MR Egger
0.60
0.55
0.50
0.45
$1/SE_{IV}$
−2.5
0.0
2.5
β_{IV}

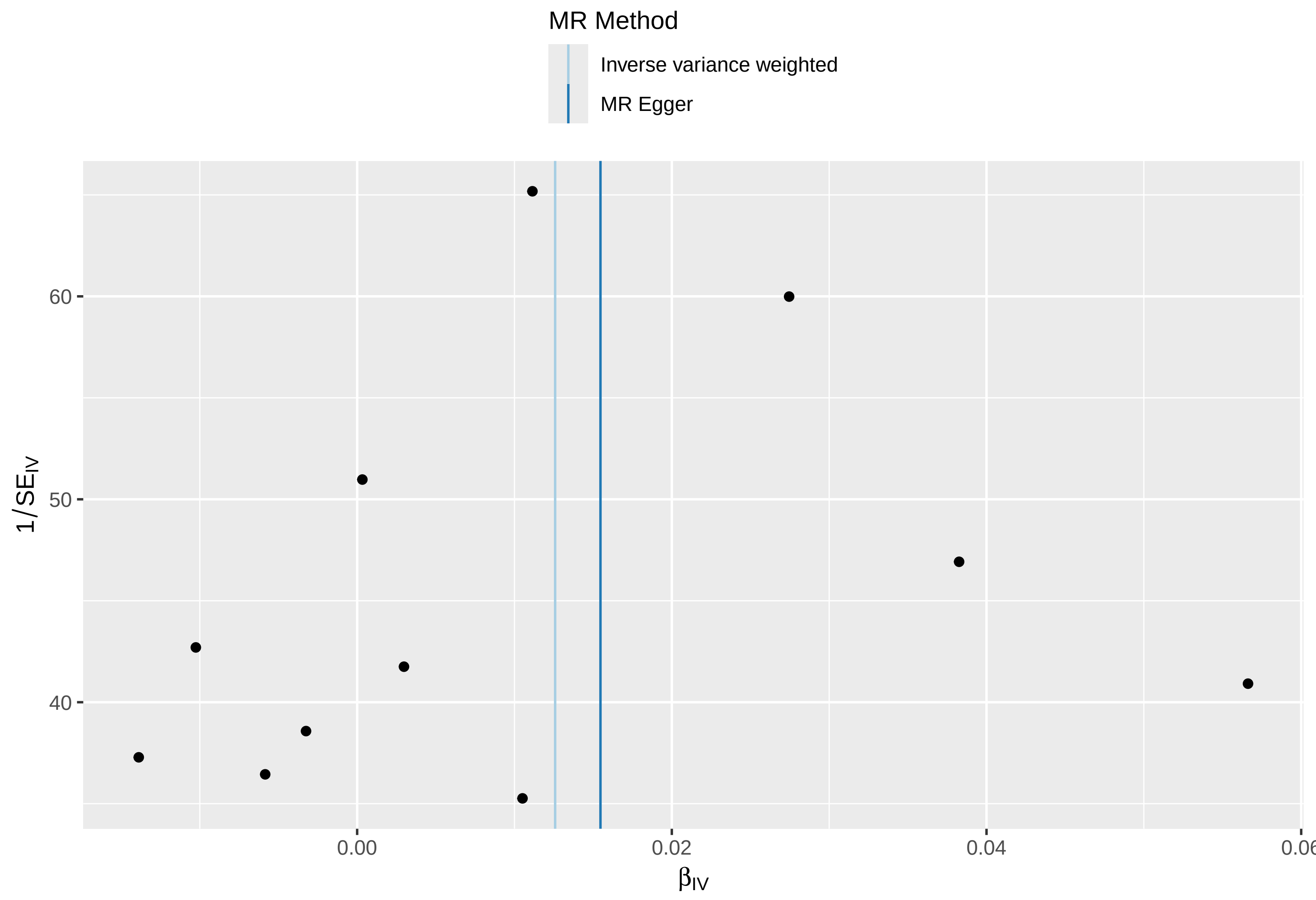
MR Method
Inverse variance weighted
MR Egger
60
50
40
$1/SE_{IV}$
0.00
0.02
0.04
0.06
β_{IV}

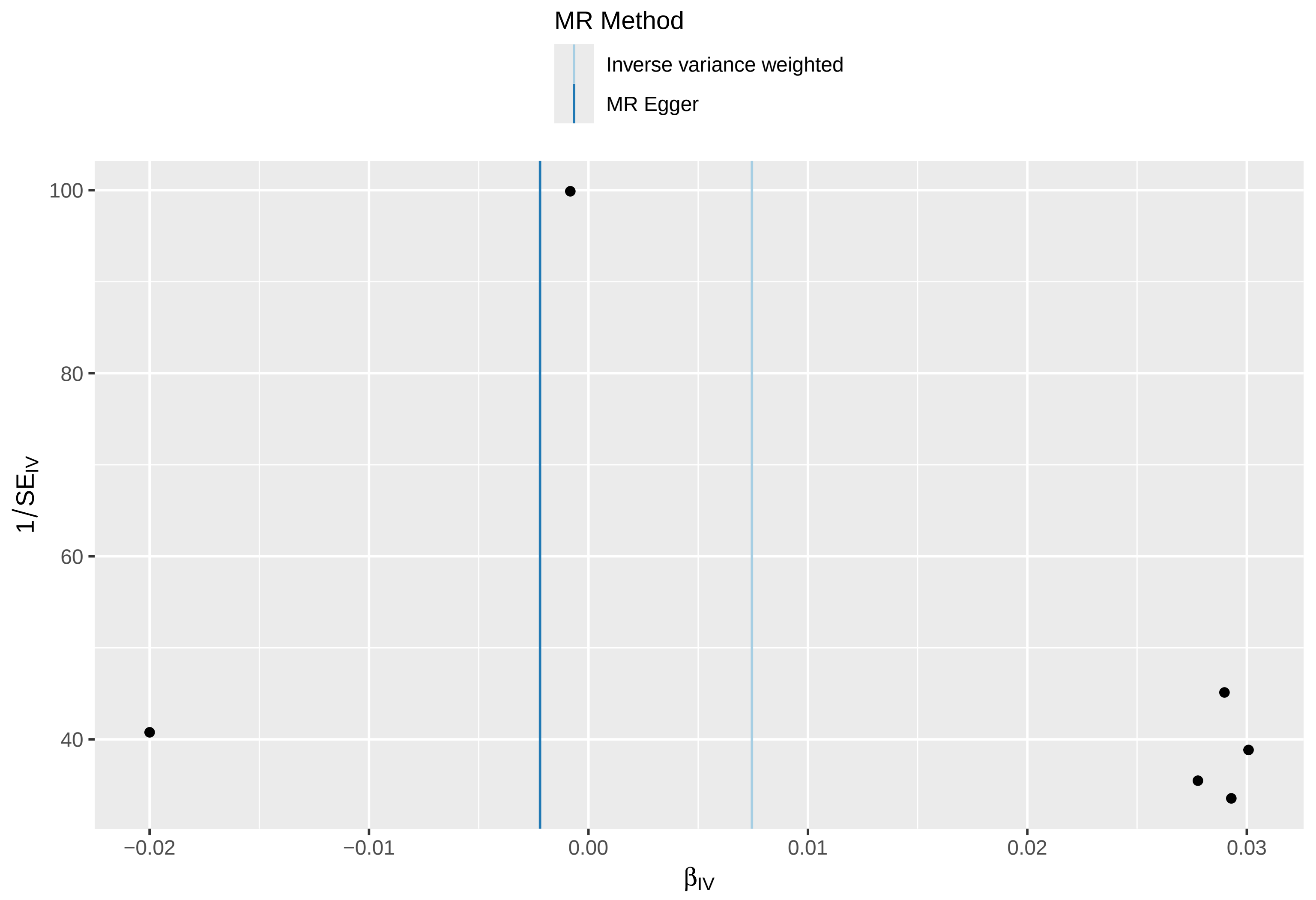

MR Method
Inverse variance weighted
MR Egger
100
80
60
40
1/SE$_{IV}$
−0.02
−0.01
0.00
0.01
0.02
0.03
β_{IV}

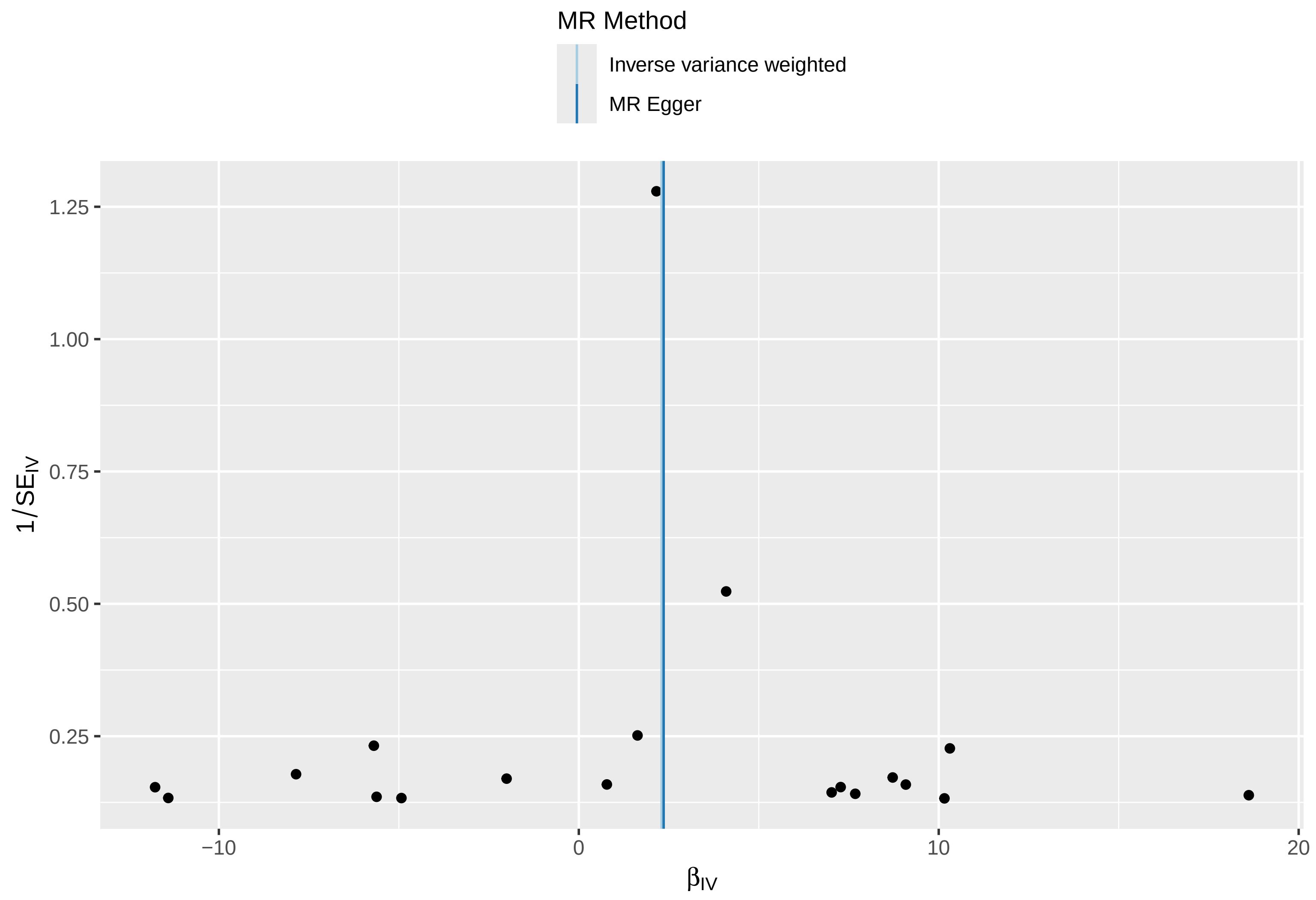
MR Method
Inverse variance weighted
MR Egger
1.25
1.00
0.75
0.50
0.25
$1/SE_{IV}$
−10
0
10
20
β_{IV}

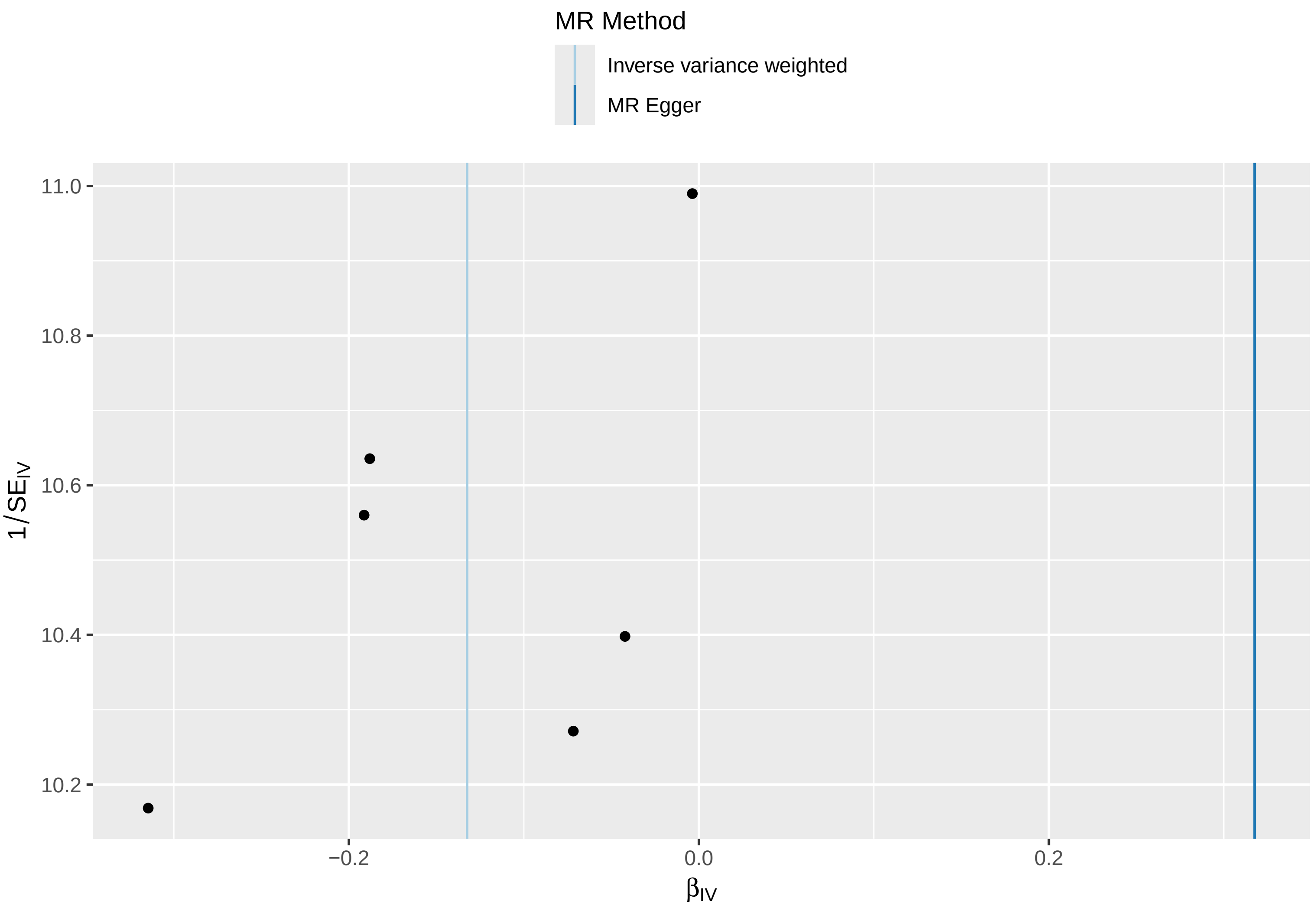
MR Method
Inverse variance weighted
MR Egger
11.0
10.8
10.6
10.4
10.2
1/SE_IV
−0.2
0.0
0.2
β_IV

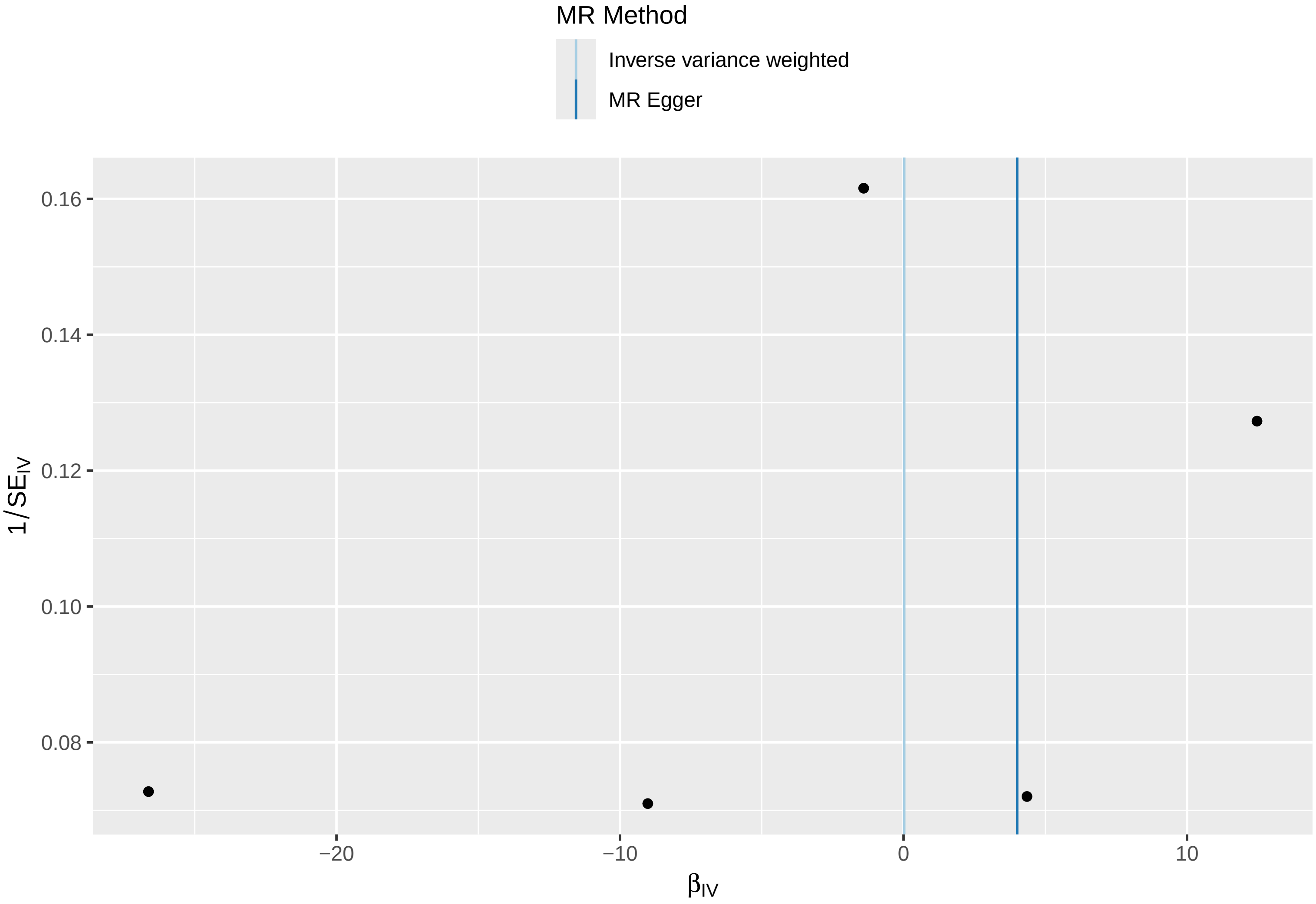
MR Method
Inverse variance weighted
MR Egger
0.16
0.14
0.12
0.10
0.08
$1/SE_{IV}$
−20
−10
0
10
β_{IV}

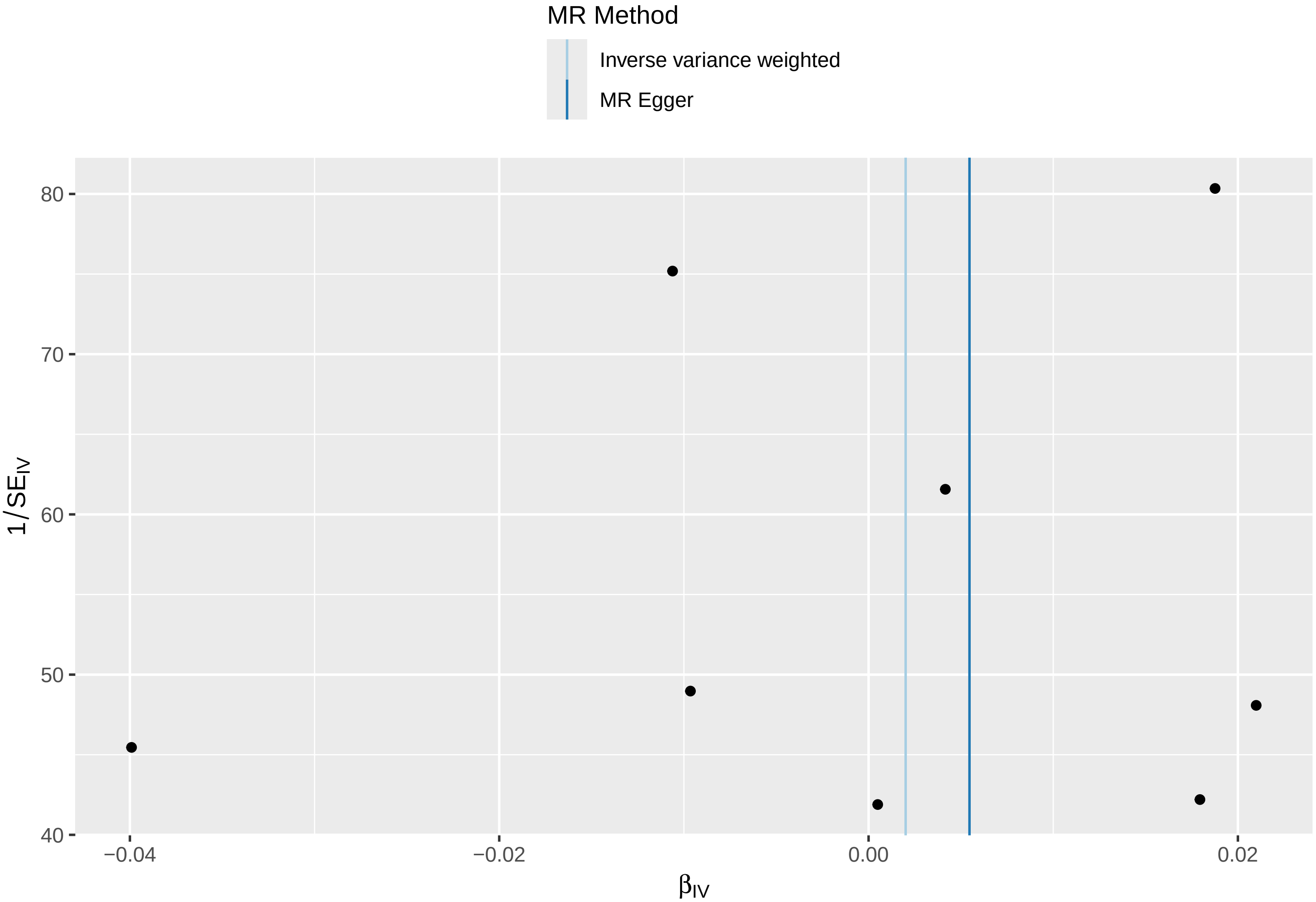
MR Method
Inverse variance weighted
MR Egger
80
70
60
50
40
1/SE$_{IV}$
−0.04
−0.02
0.00
0.02
β$_{IV}$

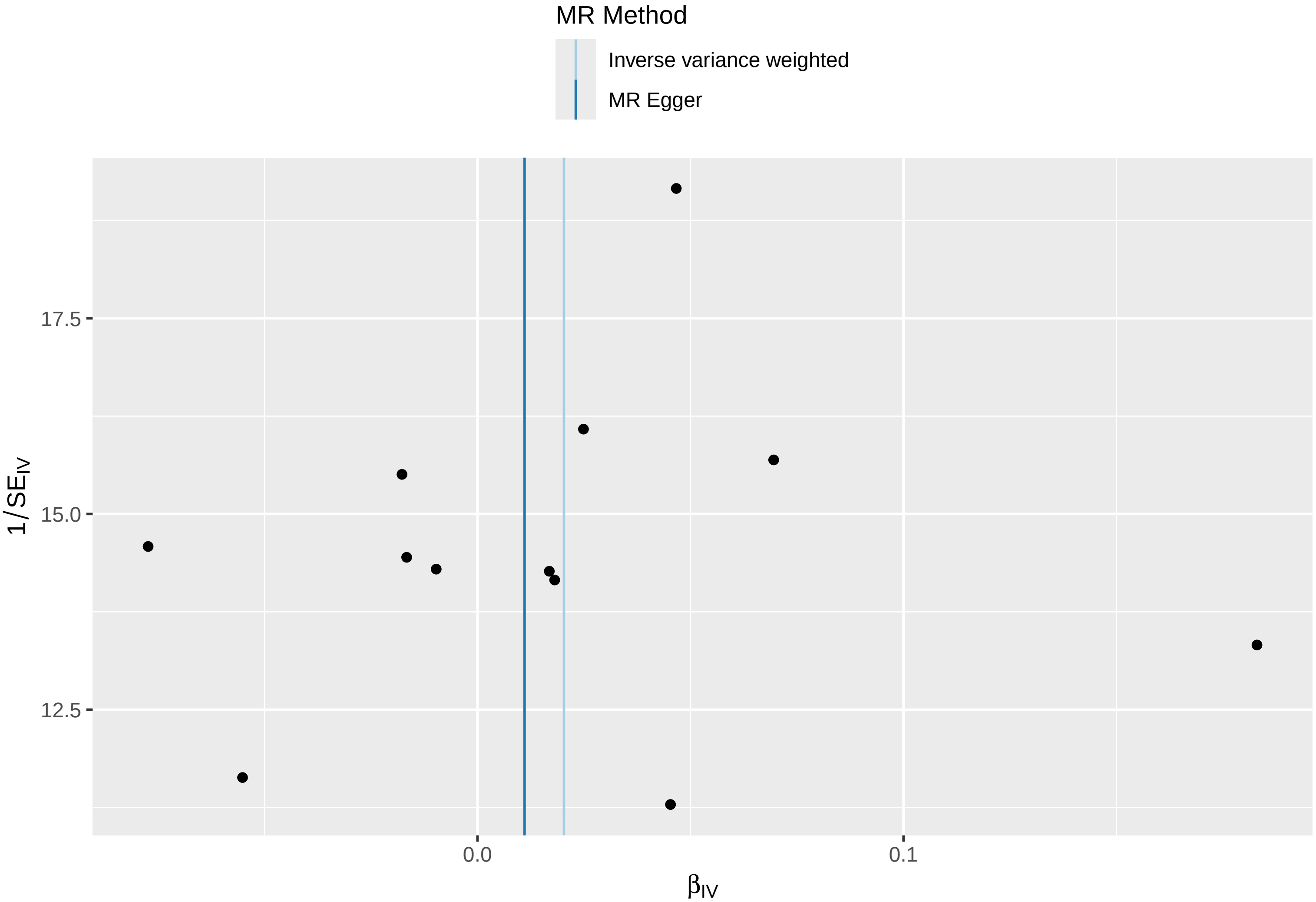

MR Method
Inverse variance weighted
MR Egger
17.5
15.0
12.5
1/SE_IV
0.0
0.1
β_IV

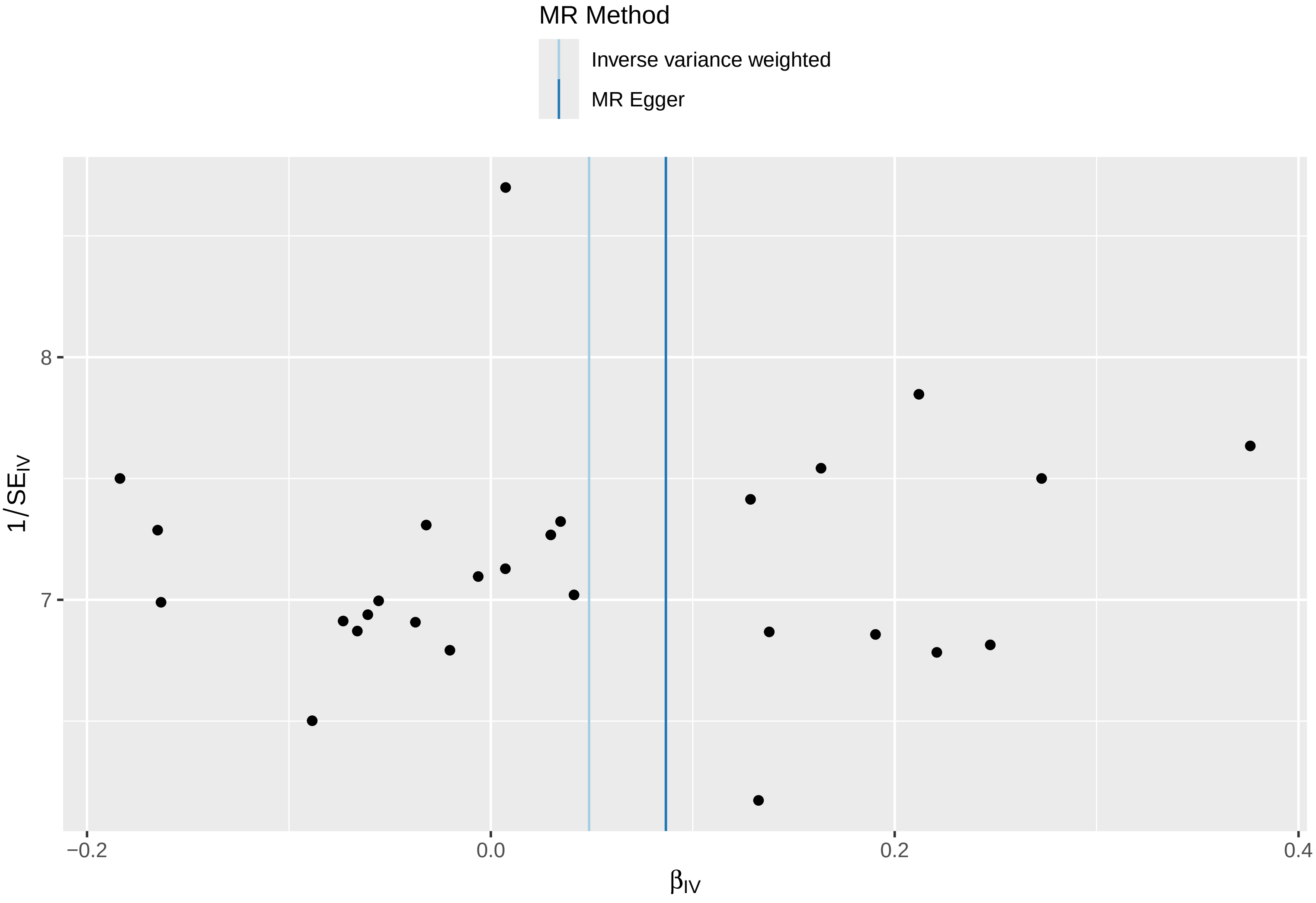

MR Method
Inverse variance weighted
MR Egger
8
7
1/SE_IV
−0.2
0.0
0.2
0.4
β_IV

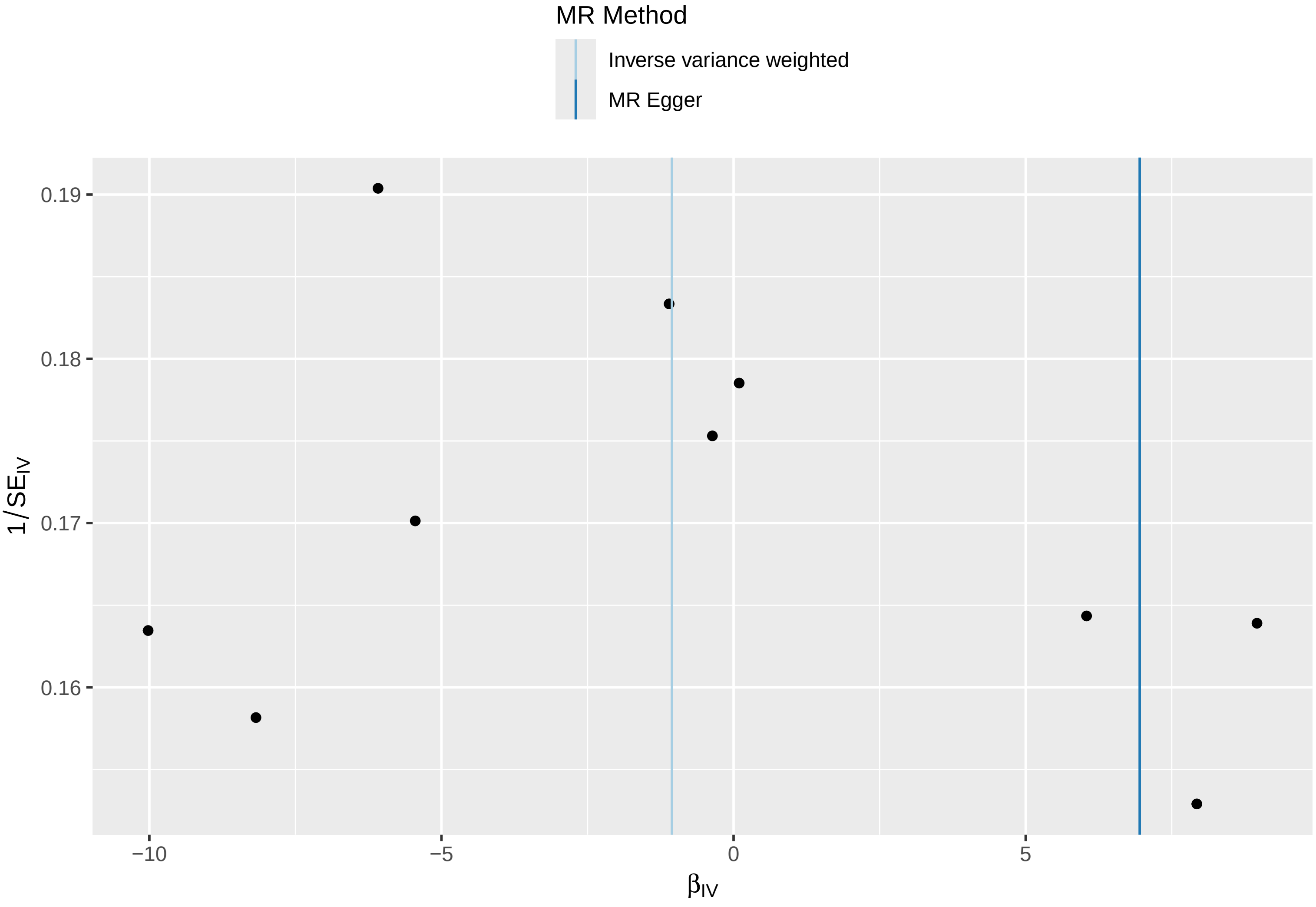
MR Method
Inverse variance weighted
MR Egger
0.19
0.18
0.17
0.16
$1/SE_{IV}$
−10
−5
0
5
β_{IV}

Supplementary file 6

Results of Leave-one-out analysis

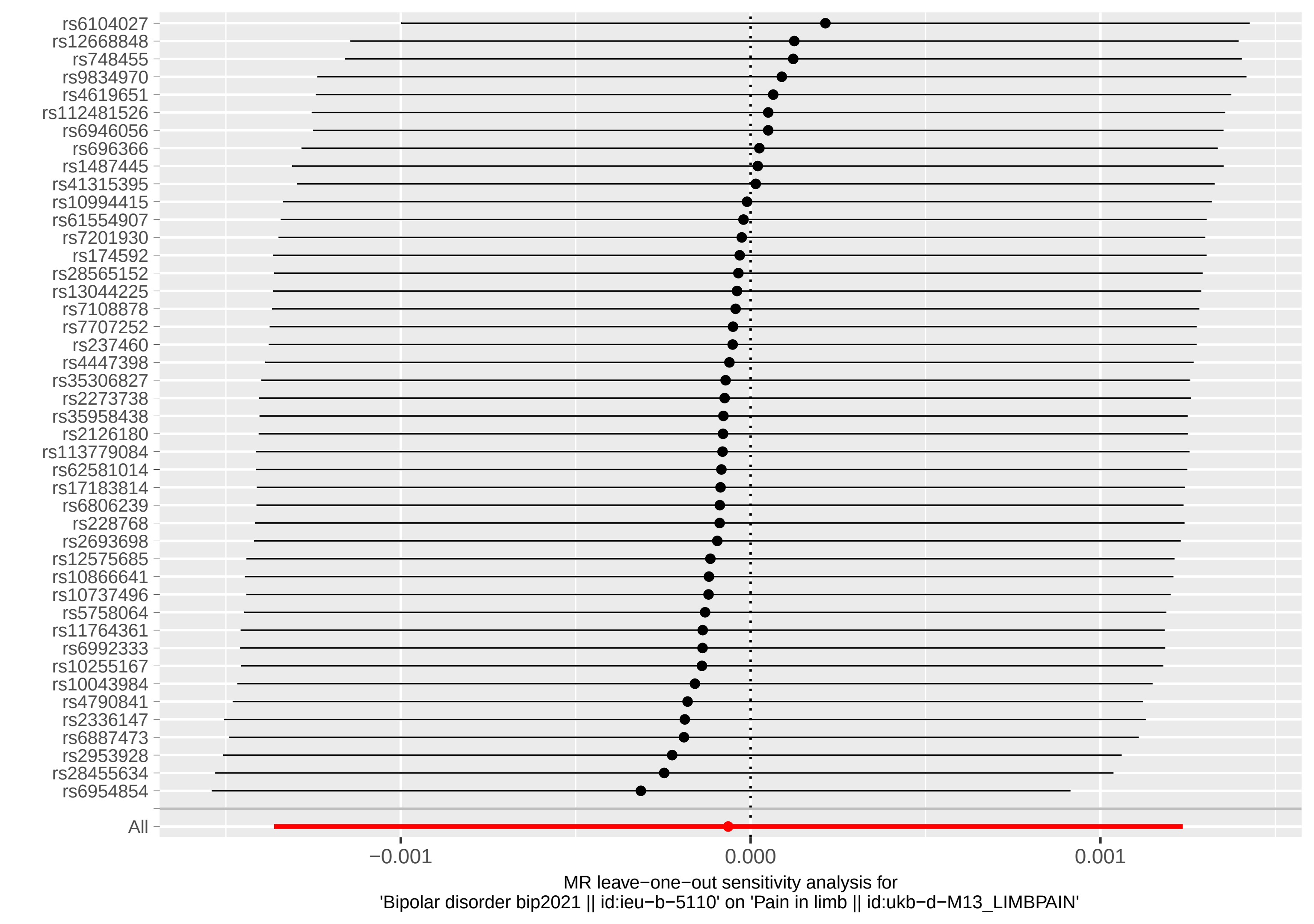

rs6104027
rs12668848
rs748455
rs9834970
rs4619651
rs112481526
rs6946056
rs696366
rs1487445
rs41315395
rs10994415
rs61554907
rs7201930
rs174592
rs28565152
rs13044225
rs7108878
rs7707252
rs237460
rs4447398
rs35306827
rs2273738
rs35958438
rs2126180
rs113779084
rs62581014
rs17183814
rs6806239
rs228768
rs2693698
rs12575685
rs10866641
rs10737496
rs5758064
rs11764361
rs6992333
rs10255167
rs10043984
rs4790841
rs2336147
rs6887473
rs2953928
rs28455634
rs6954854
All
−0.001
0.000
0.001
MR leave−one−out sensitivity analysis for
'Bipolar disorder bip2021 || id:ieu−b−5110' on 'Pain in limb || id:ukb−d−M13_LIMBPAIN'

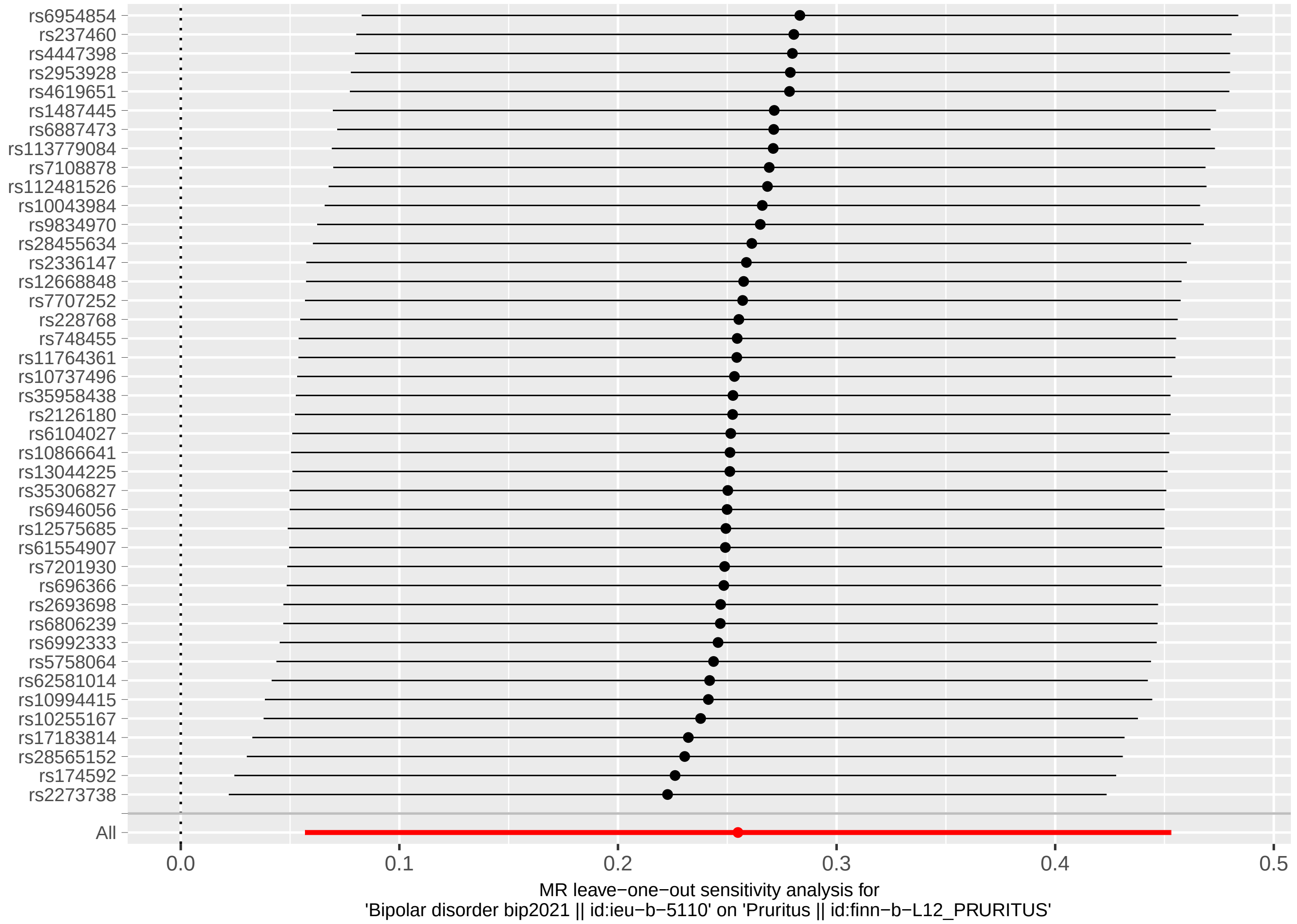
rs6954854
rs237460
rs4447398
rs2953928
rs4619651
rs1487445
rs6887473
rs113779084
rs7108878
rs112481526
rs10043984
rs9834970
rs28455634
rs2336147
rs12668848
rs7707252
rs228768
rs748455
rs11764361
rs10737496
rs35958438
rs2126180
rs6104027
rs10866641
rs13044225
rs35306827
rs6946056
rs12575685
rs61554907
rs7201930
rs696366
rs2693698
rs6806239
rs6992333
rs5758064
rs62581014
rs10994415
rs10255167
rs17183814
rs28565152
rs174592
rs2273738
All
0.0
0.1
0.2
0.3
0.4
0.5
MR leave−one−out sensitivity analysis for
'Bipolar disorder bip2021 || id:ieu−b−5110' on 'Pruritus || id:finn−b−L12_PRURITUS'

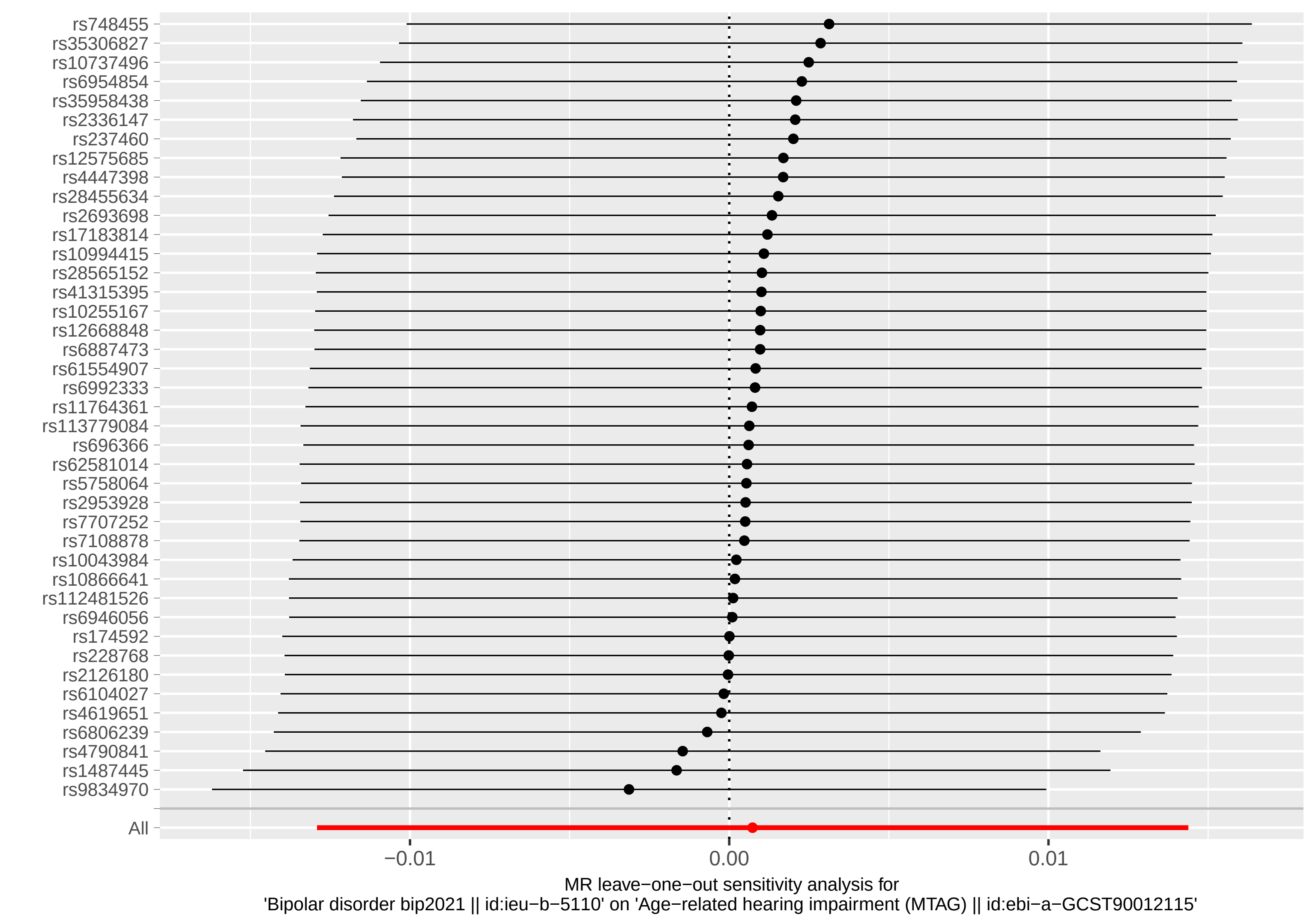
rs748455
rs35306827
rs10737496
rs6954854
rs35958438
rs2336147
rs237460
rs12575685
rs4447398
rs28455634
rs2693698
rs17183814
rs10994415
rs28565152
rs41315395
rs10255167
rs12668848
rs6887473
rs61554907
rs6992333
rs11764361
rs113779084
rs696366
rs62581014
rs5758064
rs2953928
rs7707252
rs7108878
rs10043984
rs10866641
rs112481526
rs6946056
rs174592
rs228768
rs2126180
rs6104027
rs4619651
rs6806239
rs4790841
rs1487445
rs9834970
All
−0.01
0.00
0.01
MR leave−one−out sensitivity analysis for
'Bipolar disorder bip2021 || id:ieu−b−5110' on 'Age−related hearing impairment (MTAG) || id:ebi−a−GCST90012115'

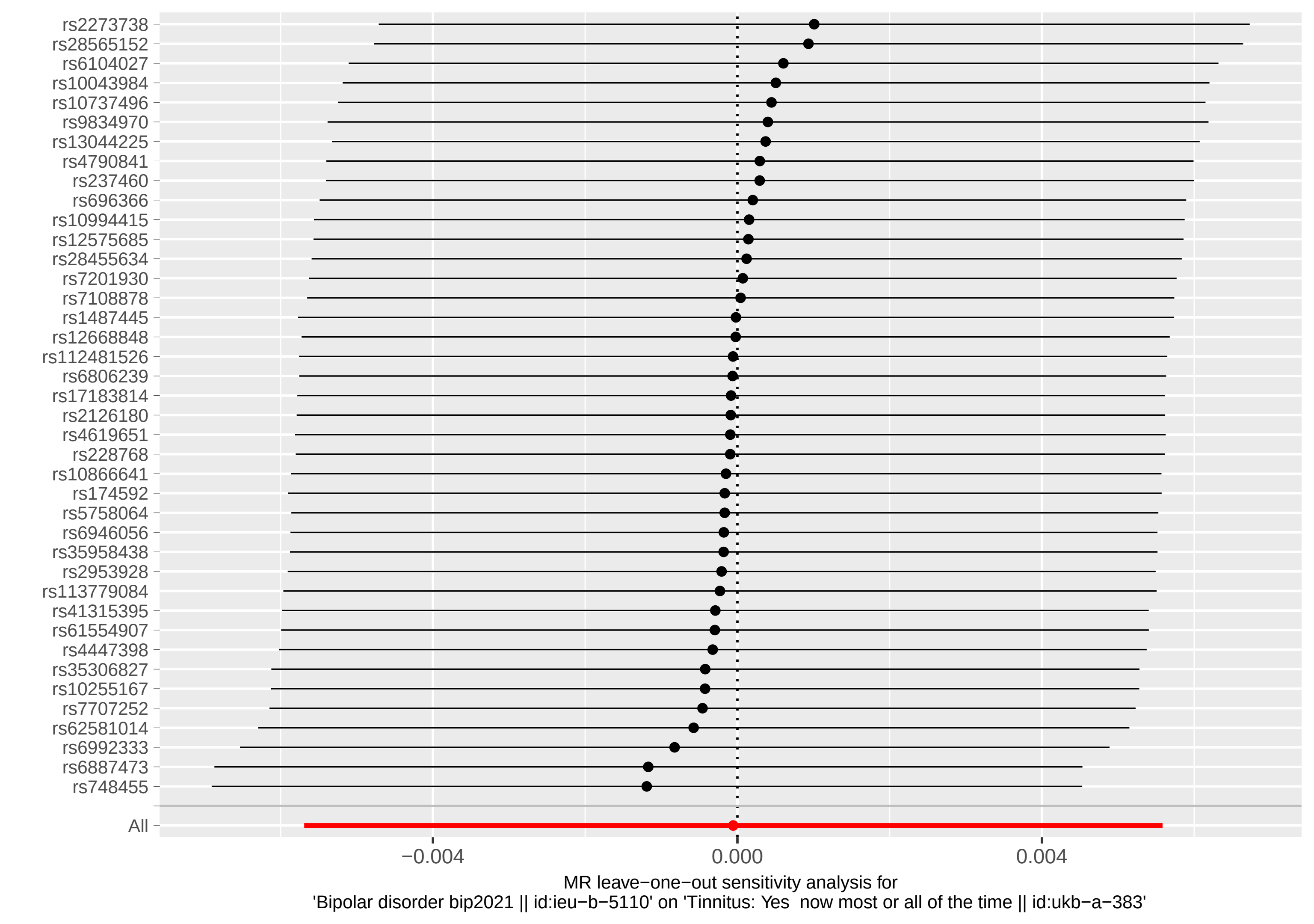
rs2273738
rs28565152
rs6104027
rs10043984
rs10737496
rs9834970
rs13044225
rs4790841
rs237460
rs696366
rs10994415
rs12575685
rs28455634
rs7201930
rs7108878
rs1487445
rs12668848
rs112481526
rs6806239
rs17183814
rs2126180
rs4619651
rs228768
rs10866641
rs174592
rs5758064
rs6946056
rs35958438
rs2953928
rs113779084
rs41315395
rs61554907
rs4447398
rs35306827
rs10255167
rs7707252
rs62581014
rs6992333
rs6887473
rs748455
All
−0.004
0.000
0.004
MR leave−one−out sensitivity analysis for
'Bipolar disorder bip2021 || id:ieu−b−5110' on 'Tinnitus: Yes now most or all of the time || id:ukb−a−383'

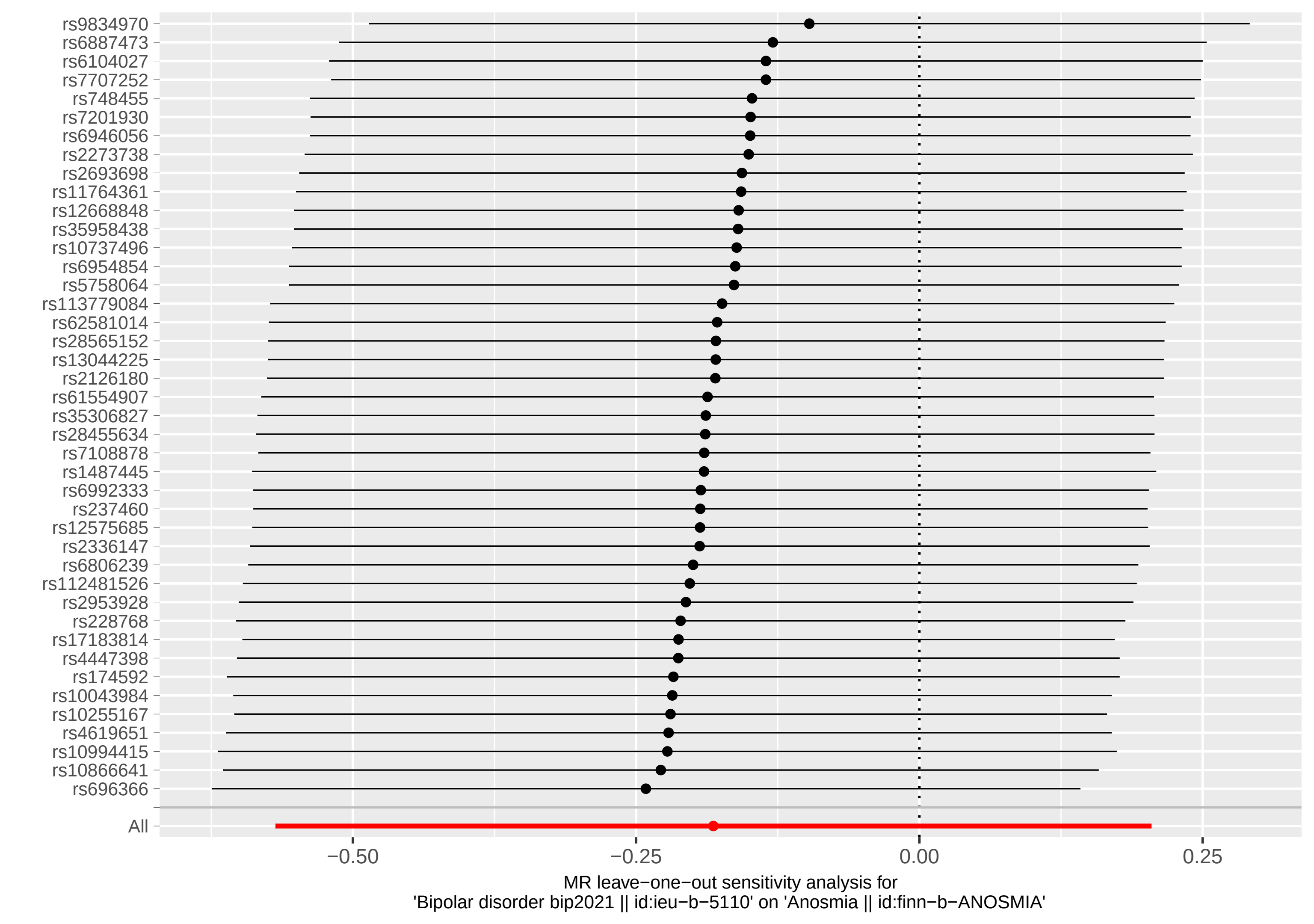

rs9834970
rs6887473
rs6104027
rs7707252
rs748455
rs7201930
rs6946056
rs2273738
rs2693698
rs11764361
rs12668848
rs35958438
rs10737496
rs6954854
rs5758064
rs113779084
rs62581014
rs28565152
rs13044225
rs2126180
rs61554907
rs35306827
rs28455634
rs7108878
rs1487445
rs6992333
rs237460
rs12575685
rs2336147
rs6806239
rs112481526
rs2953928
rs228768
rs17183814
rs4447398
rs174592
rs10043984
rs10255167
rs4619651
rs10994415
rs10866641
rs696366
All
−0.50
−0.25
0.00
0.25
MR leave−one−out sensitivity analysis for
'Bipolar disorder bip2021 || id:ieu−b−5110' on 'Anosmia || id:finn−b−ANOSMIA'

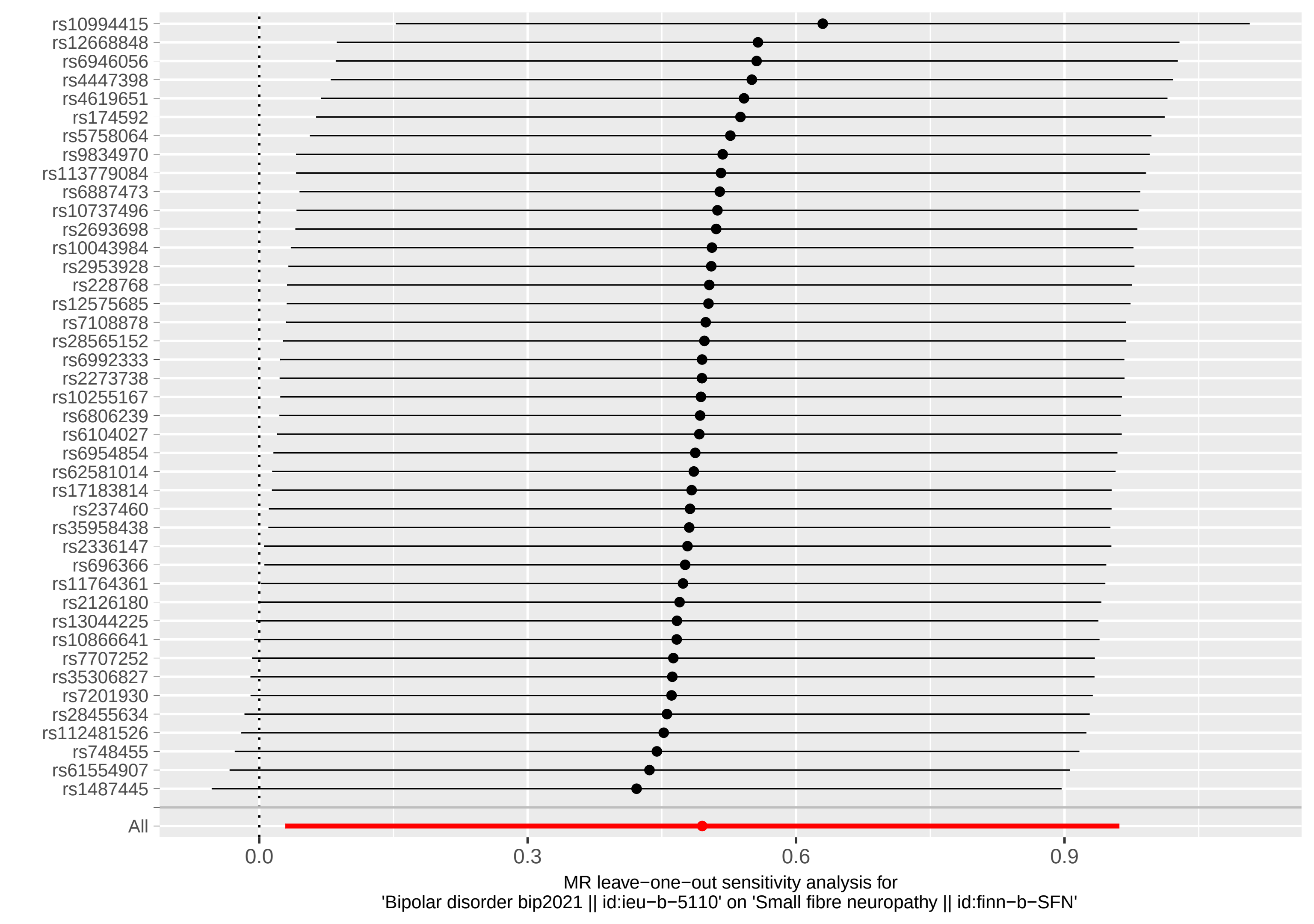
rs10994415
rs12668848
rs6946056
rs4447398
rs4619651
rs174592
rs5758064
rs9834970
rs113779084
rs6887473
rs10737496
rs2693698
rs10043984
rs2953928
rs228768
rs12575685
rs7108878
rs28565152
rs6992333
rs2273738
rs10255167
rs6806239
rs6104027
rs6954854
rs62581014
rs17183814
rs237460
rs35958438
rs2336147
rs696366
rs11764361
rs2126180
rs13044225
rs10866641
rs7707252
rs35306827
rs7201930
rs28455634
rs112481526
rs748455
rs61554907
rs1487445
All
0.0
0.3
0.6
0.9
MR leave−one−out sensitivity analysis for
'Bipolar disorder bip2021 || id:ieu−b−5110' on 'Small fibre neuropathy || id:finn−b−SFN'

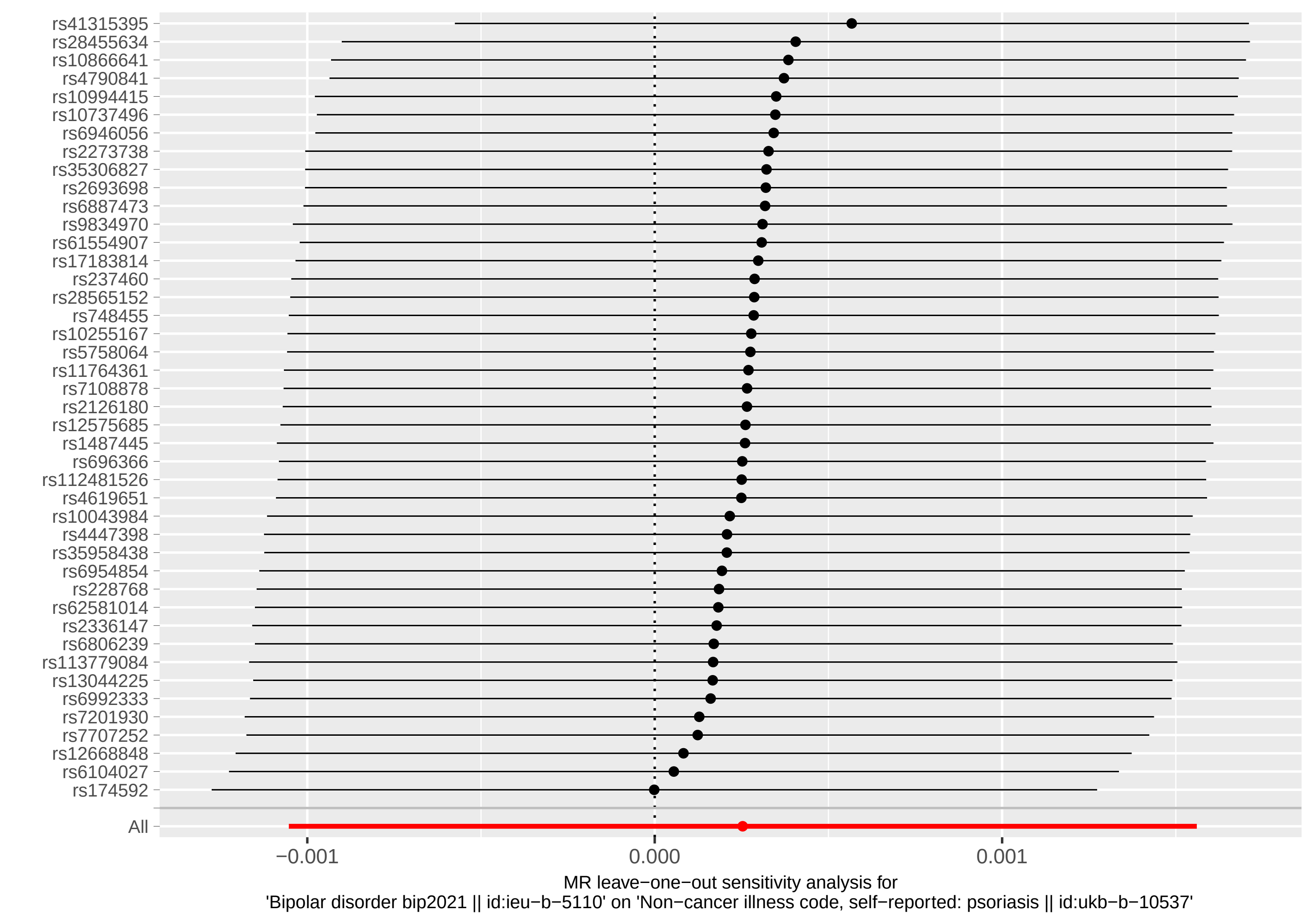
rs41315395
rs28455634
rs10866641
rs4790841
rs10994415
rs10737496
rs6946056
rs2273738
rs35306827
rs2693698
rs6887473
rs9834970
rs61554907
rs17183814
rs237460
rs28565152
rs748455
rs10255167
rs5758064
rs11764361
rs7108878
rs2126180
rs12575685
rs1487445
rs696366
rs112481526
rs4619651
rs10043984
rs4447398
rs35958438
rs6954854
rs228768
rs62581014
rs2336147
rs6806239
rs113779084
rs13044225
rs6992333
rs7201930
rs7707252
rs12668848
rs6104027
rs174592
All
−0.001
0.000
0.001
MR leave−one−out sensitivity analysis for
'Bipolar disorder bip2021 || id:ieu−b−5110' on 'Non−cancer illness code, self−reported: psoriasis || id:ukb−b−10537'

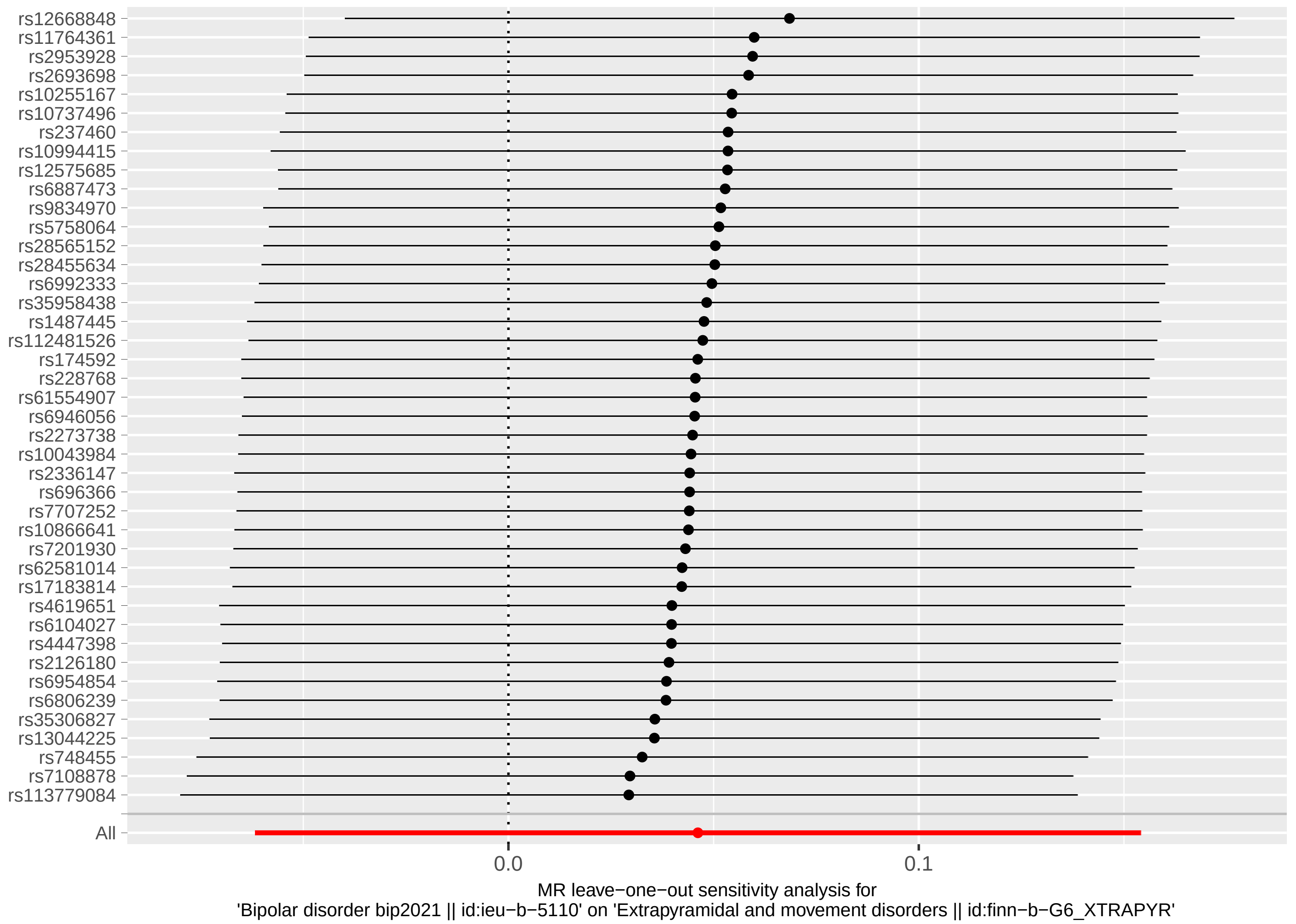
rs12668848
rs11764361
rs2953928
rs2693698
rs10255167
rs10737496
rs237460
rs10994415
rs12575685
rs6887473
rs9834970
rs5758064
rs28565152
rs28455634
rs6992333
rs35958438
rs1487445
rs112481526
rs174592
rs228768
rs61554907
rs6946056
rs2273738
rs10043984
rs2336147
rs696366
rs7707252
rs10866641
rs7201930
rs62581014
rs17183814
rs4619651
rs6104027
rs4447398
rs2126180
rs6954854
rs6806239
rs35306827
rs13044225
rs748455
rs7108878
rs113779084
All
0.0
0.1
MR leave−one−out sensitivity analysis for
'Bipolar disorder bip2021 || id:ieu−b−5110' on 'Extrapyramidal and movement disorders || id:finn−b−G6_XTRAPYR'

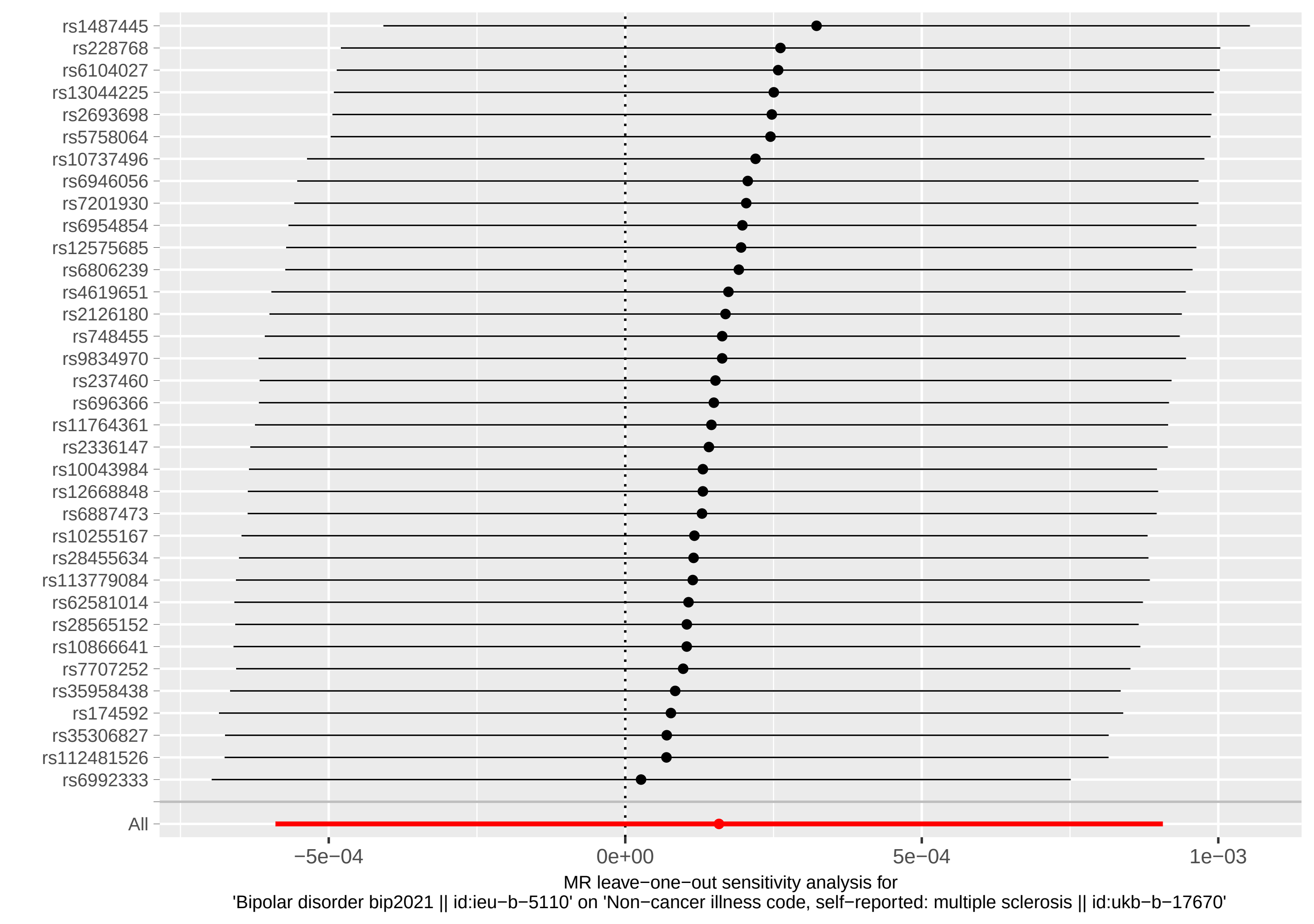
rs1487445
rs228768
rs6104027
rs13044225
rs2693698
rs5758064
rs10737496
rs6946056
rs7201930
rs6954854
rs12575685
rs6806239
rs4619651
rs2126180
rs748455
rs9834970
rs237460
rs696366
rs11764361
rs2336147
rs10043984
rs12668848
rs6887473
rs10255167
rs28455634
rs113779084
rs62581014
rs28565152
rs10866641
rs7707252
rs35958438
rs174592
rs35306827
rs112481526
rs6992333
All
−5e−04
0e+00
5e−04
1e−03
MR leave−one−out sensitivity analysis for
'Bipolar disorder bip2021 || id:ieu−b−5110' on 'Non−cancer illness code, self−reported: multiple sclerosis || id:ukb−b−17670'

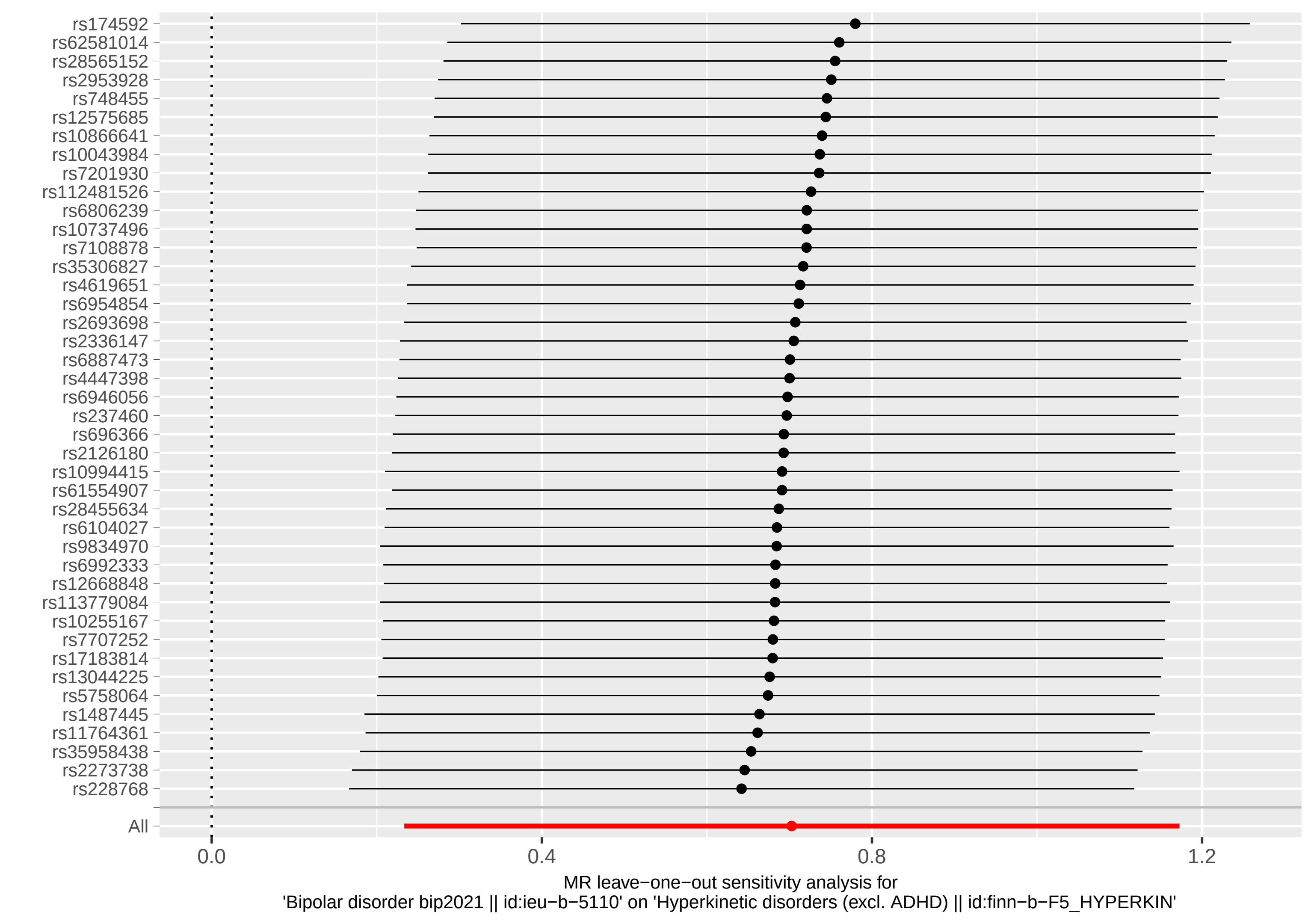

rs174592
rs62581014
rs28565152
rs2953928
rs748455
rs12575685
rs10866641
rs10043984
rs7201930
rs112481526
rs6806239
rs10737496
rs7108878
rs35306827
rs4619651
rs6954854
rs2693698
rs2336147
rs6887473
rs4447398
rs6946056
rs237460
rs696366
rs2126180
rs10994415
rs61554907
rs28455634
rs6104027
rs9834970
rs6992333
rs12668848
rs113779084
rs10255167
rs7707252
rs17183814
rs13044225
rs5758064
rs1487445
rs11764361
rs35958438
rs2273738
rs228768
All
0.0
0.4
0.8
1.2
MR leave−one−out sensitivity analysis for
'Bipolar disorder bip2021 || id:ieu−b−5110' on 'Hyperkinetic disorders (excl. ADHD) || id:finn−b−F5_HYPERKIN'

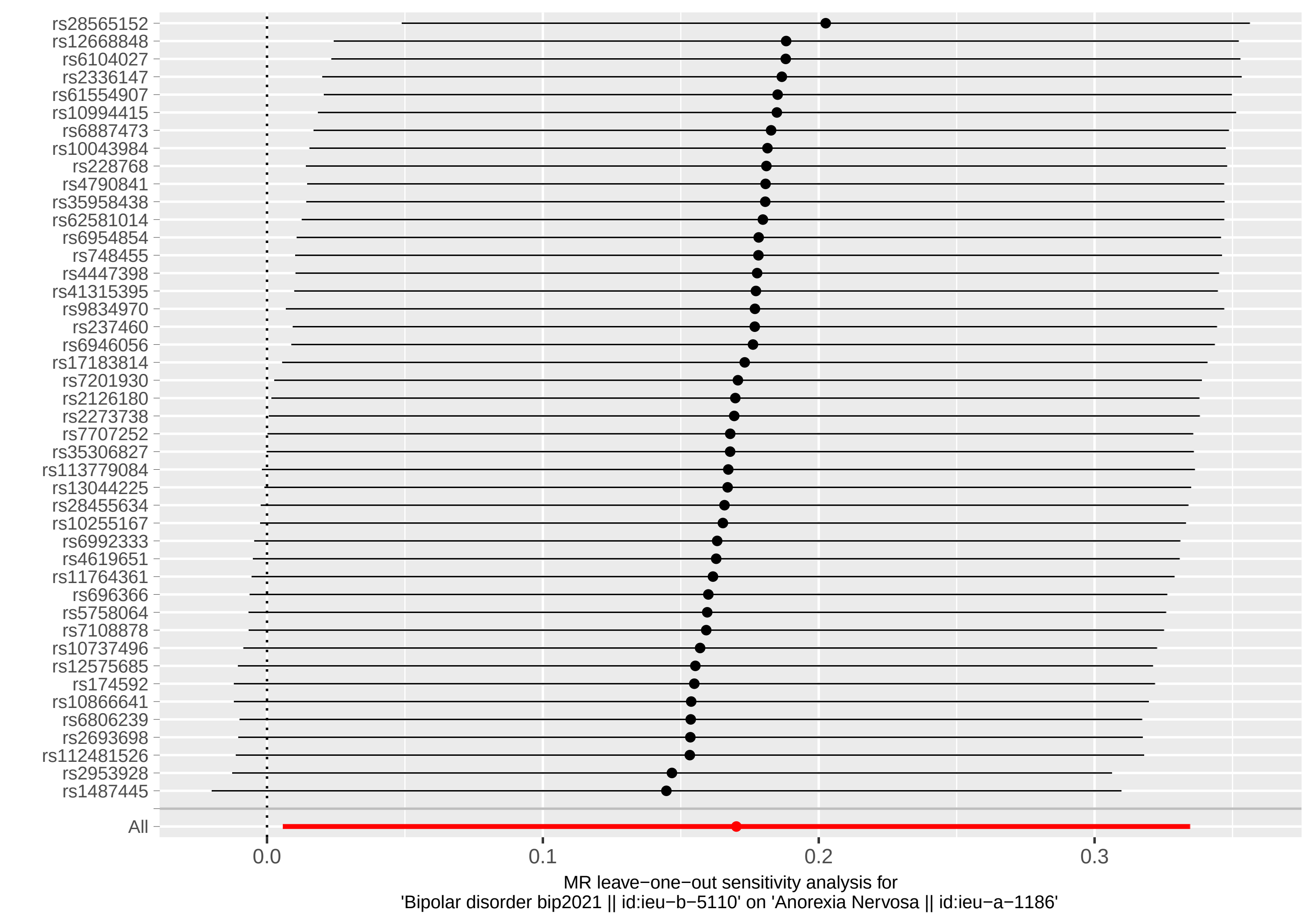
rs28565152
rs12668848
rs6104027
rs2336147
rs61554907
rs10994415
rs6887473
rs10043984
rs228768
rs4790841
rs35958438
rs62581014
rs6954854
rs748455
rs4447398
rs41315395
rs9834970
rs237460
rs6946056
rs17183814
rs7201930
rs2126180
rs2273738
rs7707252
rs35306827
rs113779084
rs13044225
rs28455634
rs10255167
rs6992333
rs4619651
rs11764361
rs696366
rs5758064
rs7108878
rs10737496
rs12575685
rs174592
rs10866641
rs6806239
rs2693698
rs112481526
rs2953928
rs1487445
All
0.0
0.1
0.2
0.3
MR leave−one−out sensitivity analysis for
'Bipolar disorder bip2021 || id:ieu−b−5110' on 'Anorexia Nervosa || id:ieu−a−1186'

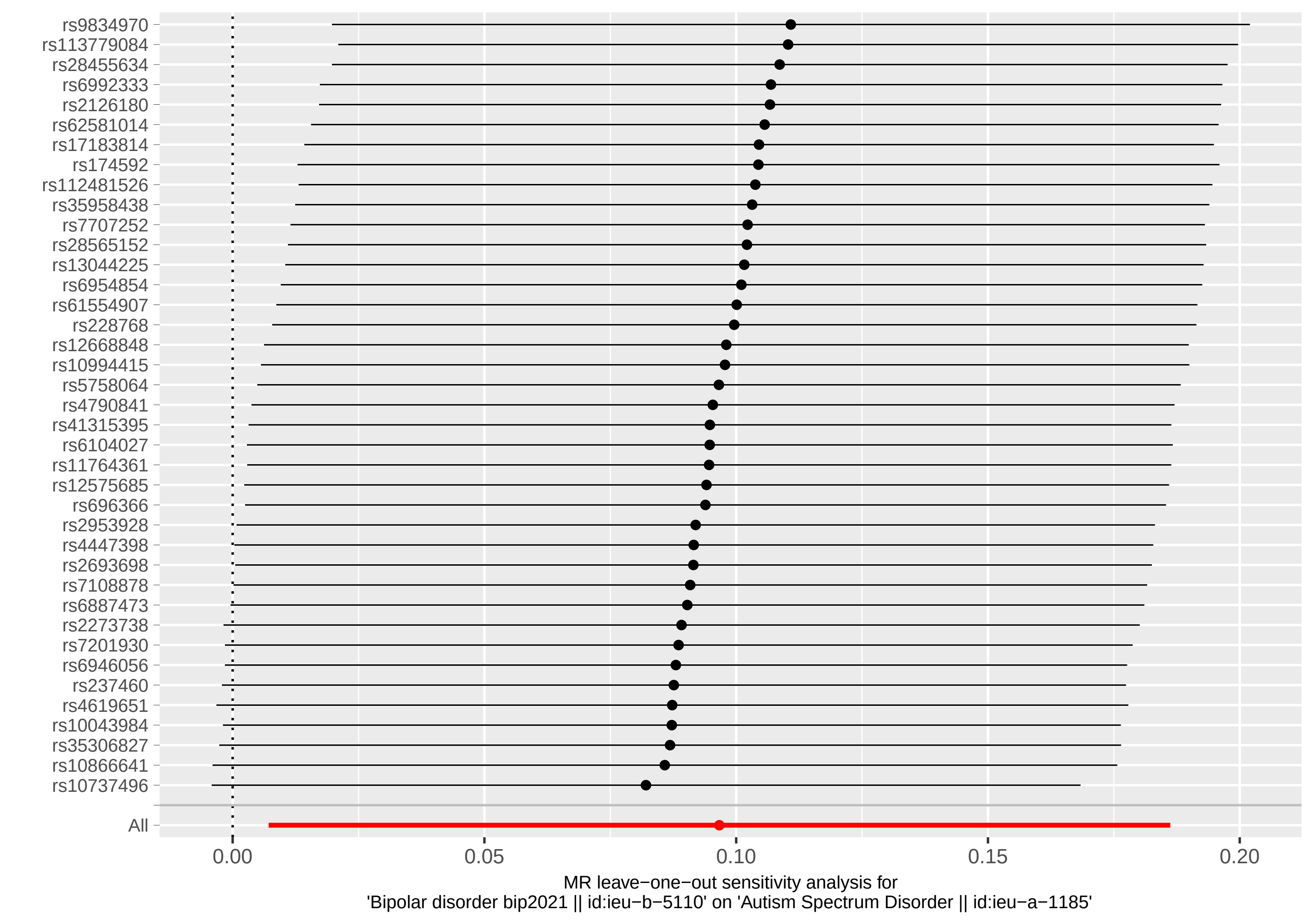
rs9834970
rs113779084
rs28455634
rs6992333
rs2126180
rs62581014
rs17183814
rs174592
rs112481526
rs35958438
rs7707252
rs28565152
rs13044225
rs6954854
rs61554907
rs228768
rs12668848
rs10994415
rs5758064
rs4790841
rs41315395
rs6104027
rs11764361
rs12575685
rs696366
rs2953928
rs4447398
rs2693698
rs7108878
rs6887473
rs2273738
rs7201930
rs6946056
rs237460
rs4619651
rs10043984
rs35306827
rs10866641
rs10737496
All
0.00
0.05
0.10
0.15
0.20
MR leave−one−out sensitivity analysis for
'Bipolar disorder bip2021 || id:ieu−b−5110' on 'Autism Spectrum Disorder || id:ieu−a−1185'

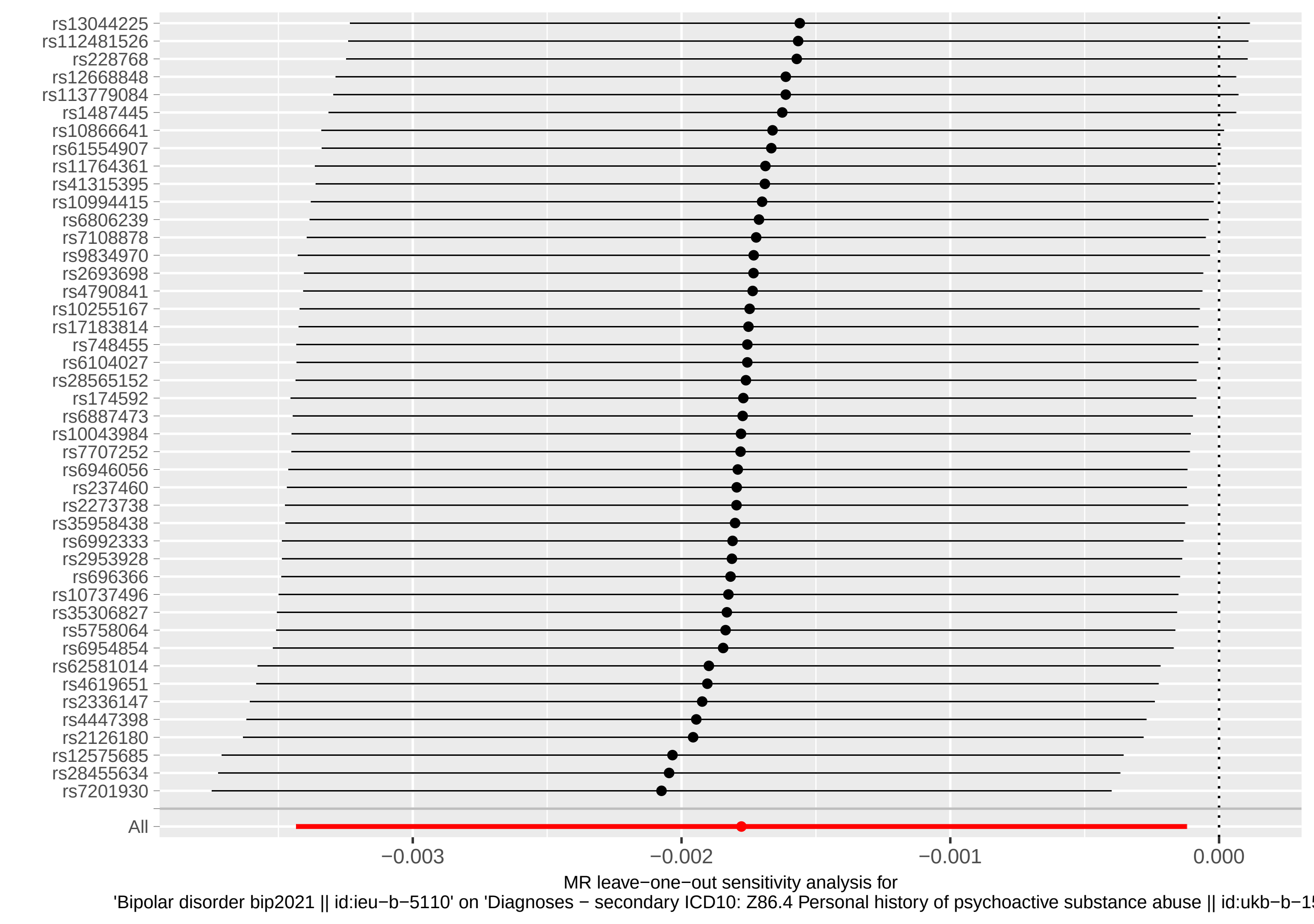
rs13044225
rs112481526
rs228768
rs12668848
rs113779084
rs1487445
rs10866641
rs61554907
rs11764361
rs41315395
rs10994415
rs6806239
rs7108878
rs9834970
rs2693698
rs4790841
rs10255167
rs17183814
rs748455
rs6104027
rs28565152
rs174592
rs6887473
rs10043984
rs7707252
rs6946056
rs237460
rs2273738
rs35958438
rs6992333
rs2953928
rs696366
rs10737496
rs35306827
rs5758064
rs6954854
rs62581014
rs4619651
rs2336147
rs4447398
rs2126180
rs12575685
rs28455634
rs7201930
All
−0.003
−0.002
−0.001
0.000
MR leave−one−out sensitivity analysis for
'Bipolar disorder bip2021 || id:ieu−b−5110' on 'Diagnoses − secondary ICD10: Z86.4 Personal history of psychoactive substance abuse || id:ukb−b−1

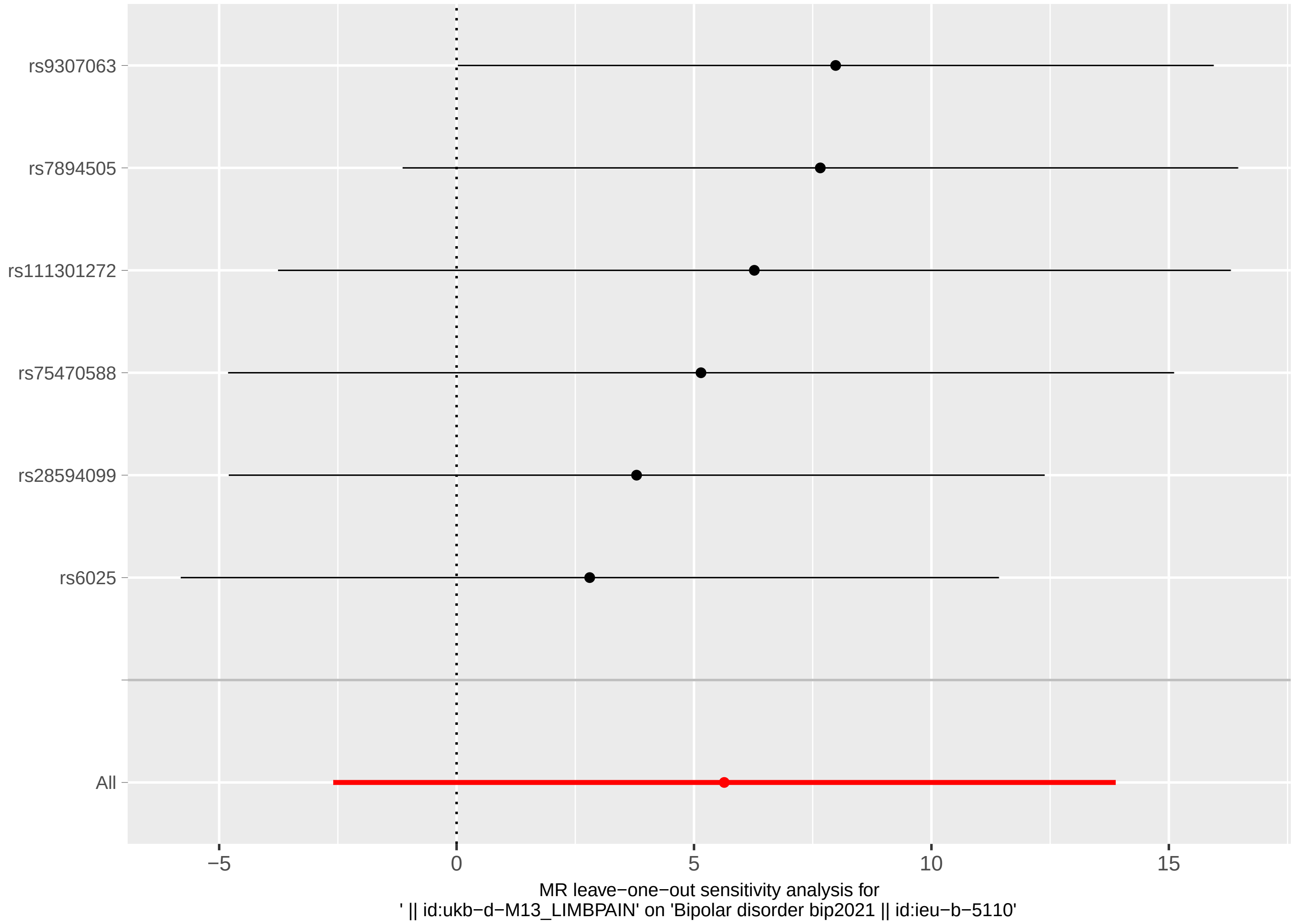
rs9307063
rs7894505
rs111301272
rs75470588
rs28594099
rs6025
All
−5
0
5
10
15
MR leave−one−out sensitivity analysis for
' || id:ukb−d−M13_LIMBPAIN' on 'Bipolar disorder bip2021 || id:ieu−b−5110'

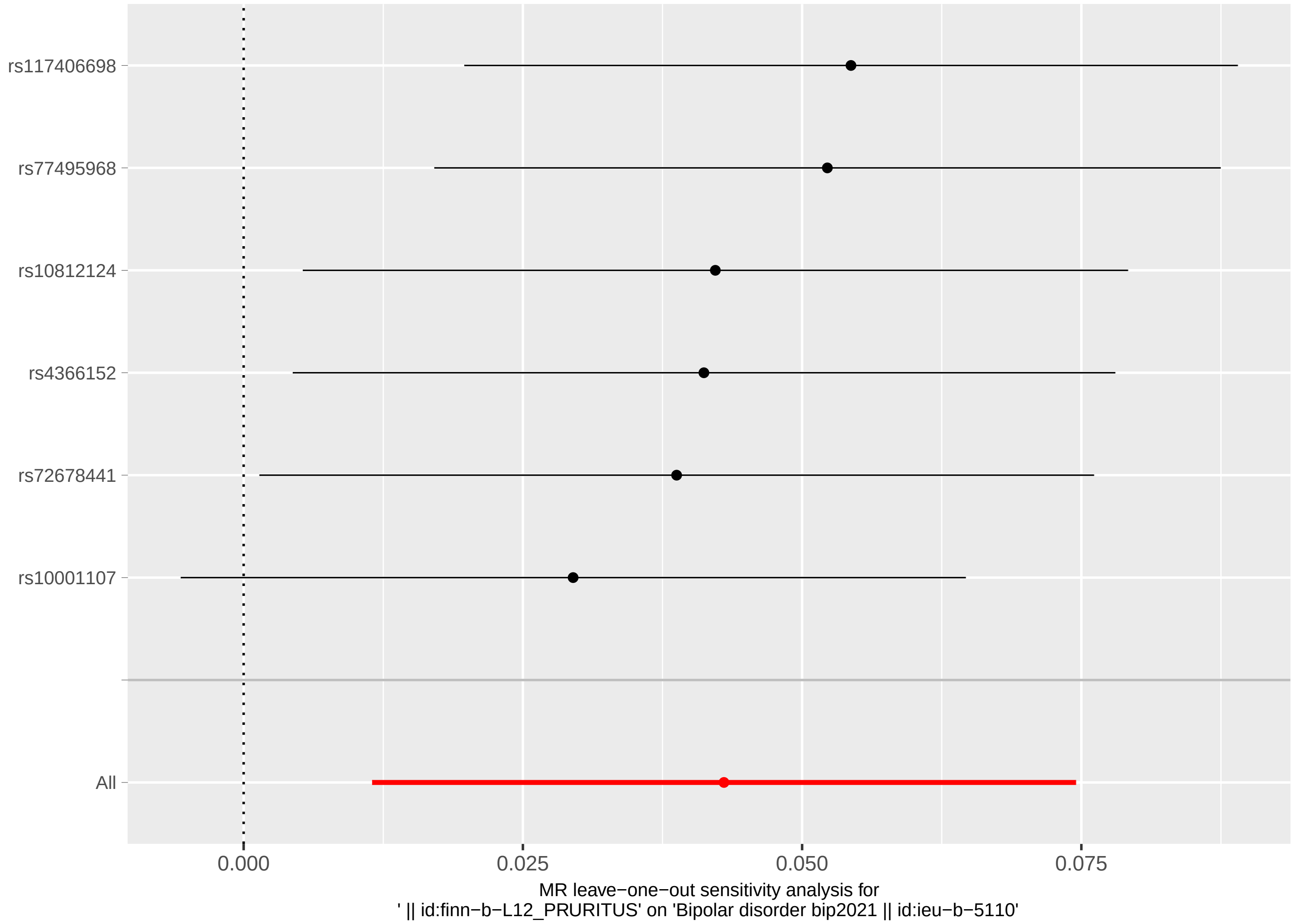
rs117406698
rs77495968
rs10812124
rs4366152
rs72678441
rs10001107
All
0.000
0.025
0.050
0.075
MR leave−one−out sensitivity analysis for
' || id:finn−b−L12_PRURITUS' on 'Bipolar disorder bip2021 || id:ieu−b−5110'

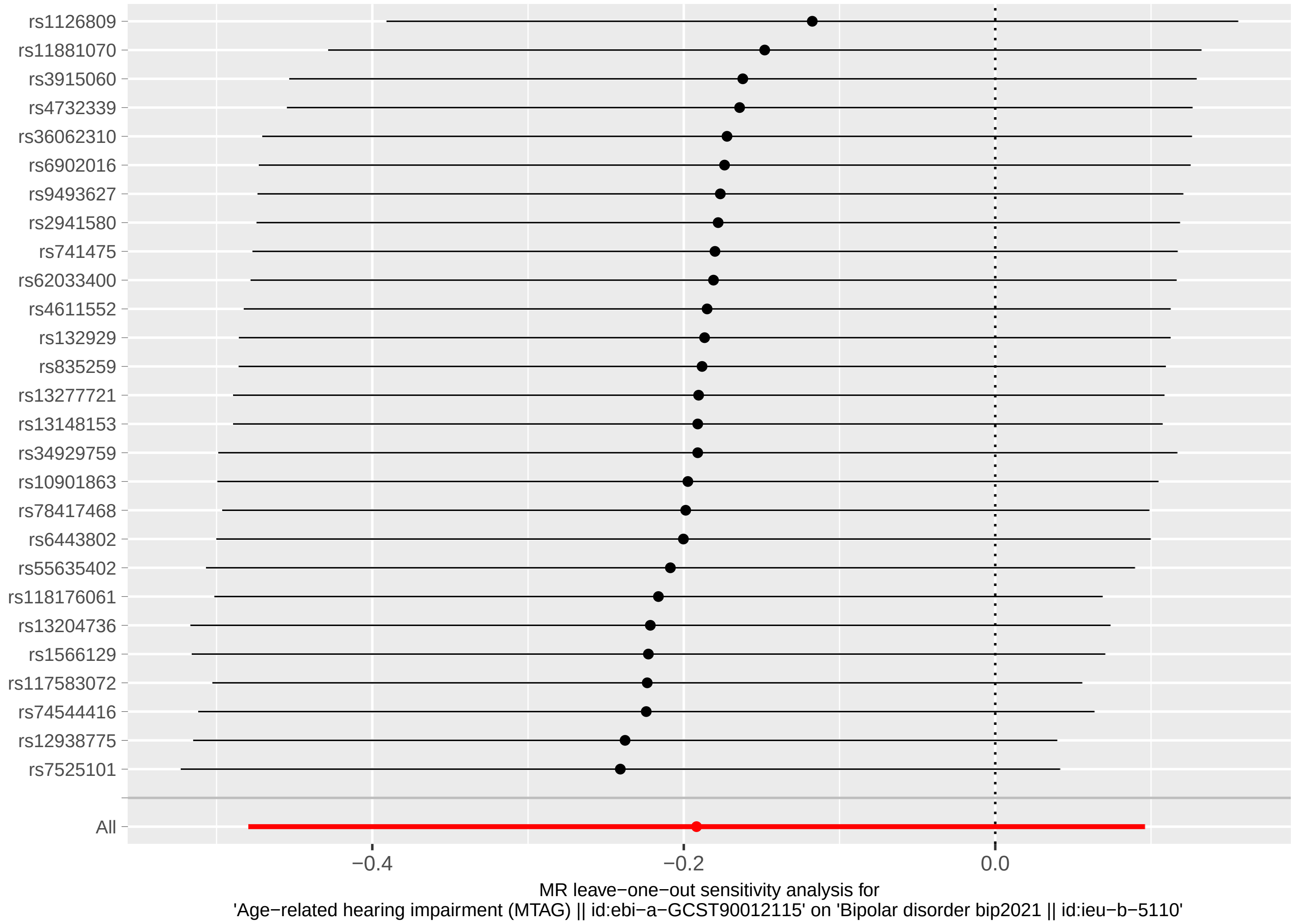
rs1126809
rs11881070
rs3915060
rs4732339
rs36062310
rs6902016
rs9493627
rs2941580
rs741475
rs62033400
rs4611552
rs132929
rs835259
rs13277721
rs13148153
rs34929759
rs10901863
rs78417468
rs6443802
rs55635402
rs118176061
rs13204736
rs1566129
rs117583072
rs74544416
rs12938775
rs7525101
All
−0.4
−0.2
0.0
MR leave−one−out sensitivity analysis for
'Age−related hearing impairment (MTAG) || id:ebi−a−GCST90012115' on 'Bipolar disorder bip2021 || id:ieu−b−5110'

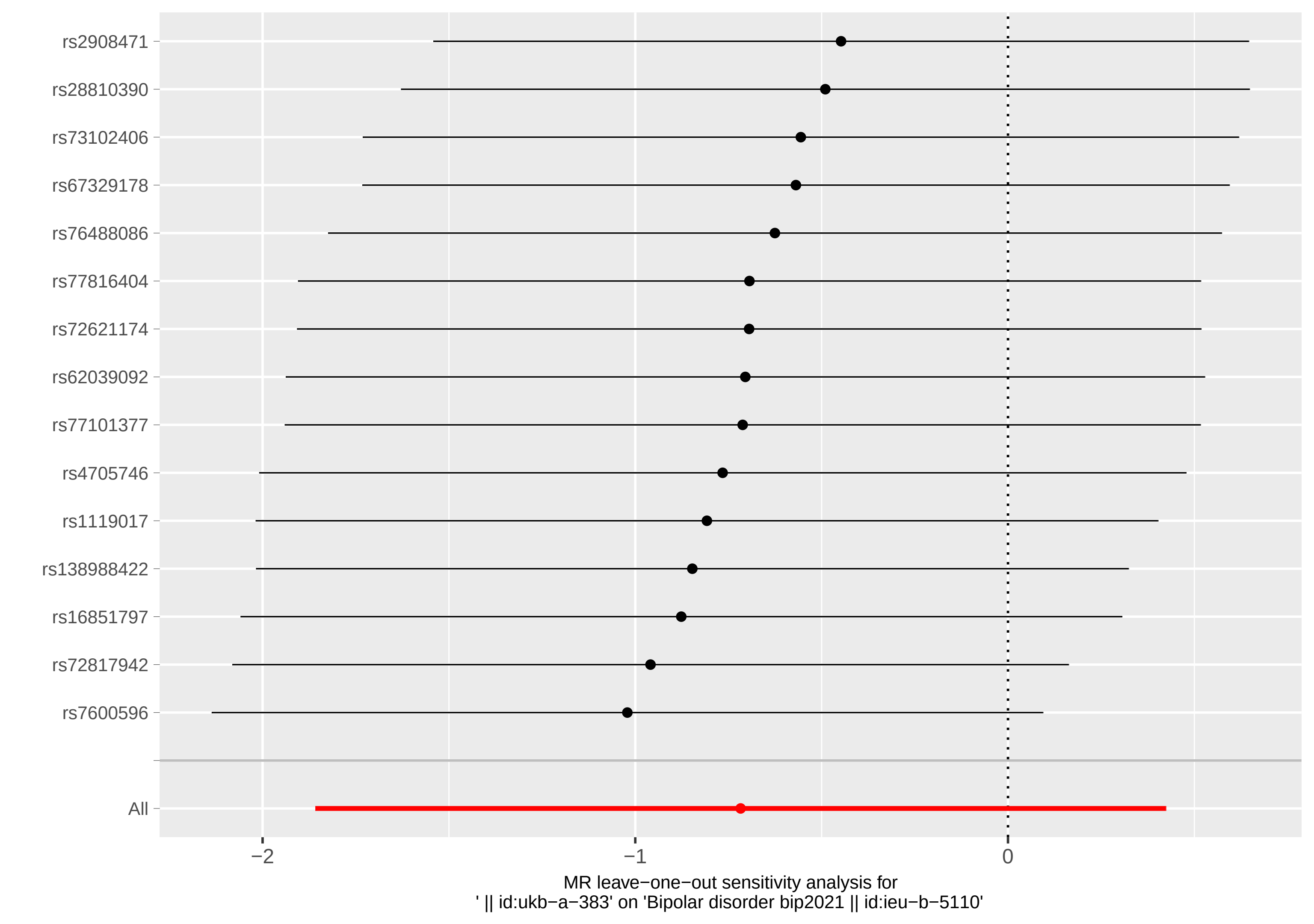
rs2908471
rs28810390
rs73102406
rs67329178
rs76488086
rs77816404
rs72621174
rs62039092
rs77101377
rs4705746
rs1119017
rs138988422
rs16851797
rs72817942
rs7600596
All
−2
−1
0
MR leave−one−out sensitivity analysis for
' || id:ukb−a−383' on 'Bipolar disorder bip2021 || id:ieu−b−5110'

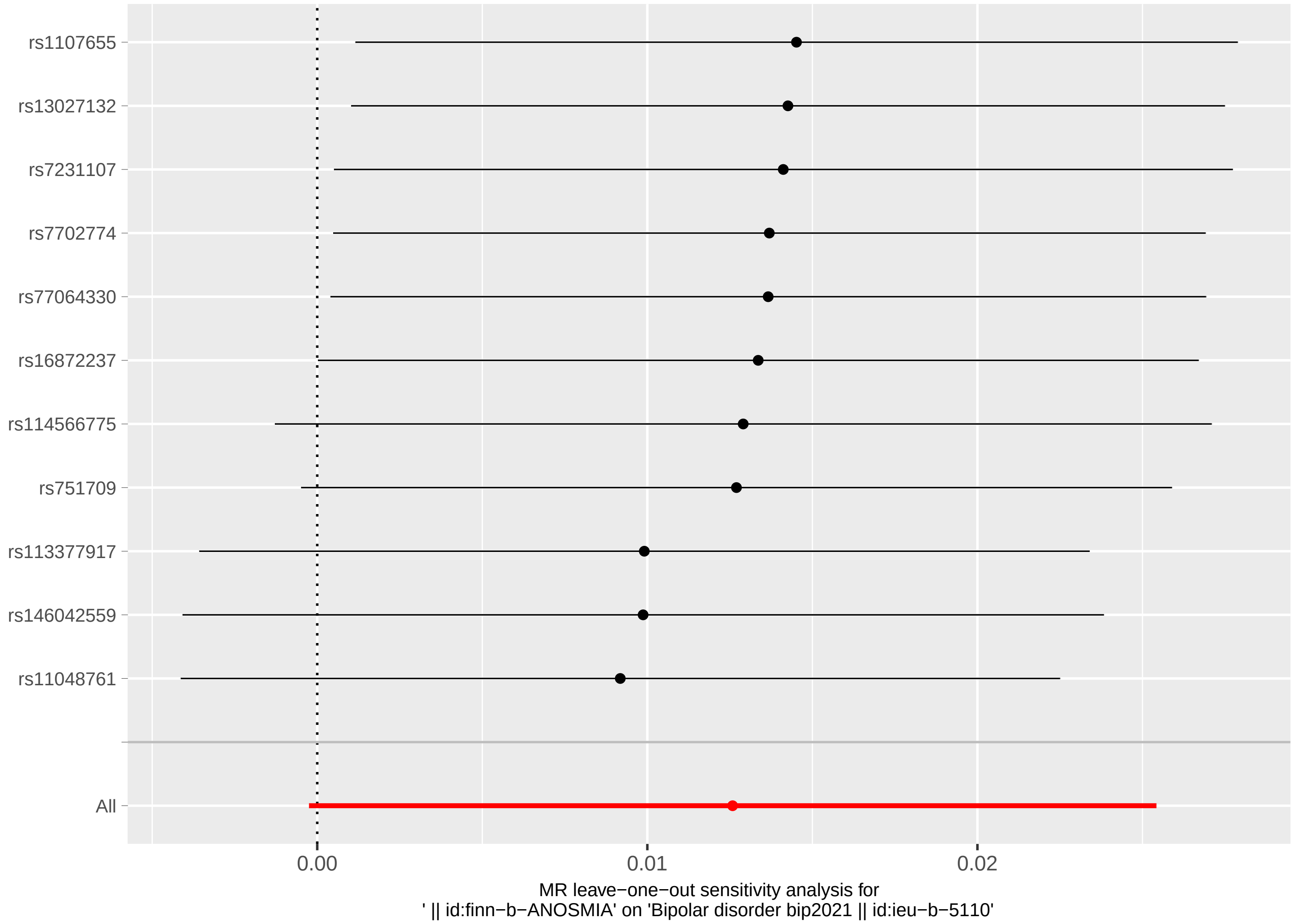
rs1107655
rs13027132
rs7231107
rs7702774
rs77064330
rs16872237
rs114566775
rs751709
rs113377917
rs146042559
rs11048761
All
0.00
0.01
0.02
MR leave−one−out sensitivity analysis for
' || id:finn−b−ANOSMIA' on 'Bipolar disorder bip2021 || id:ieu−b−5110'

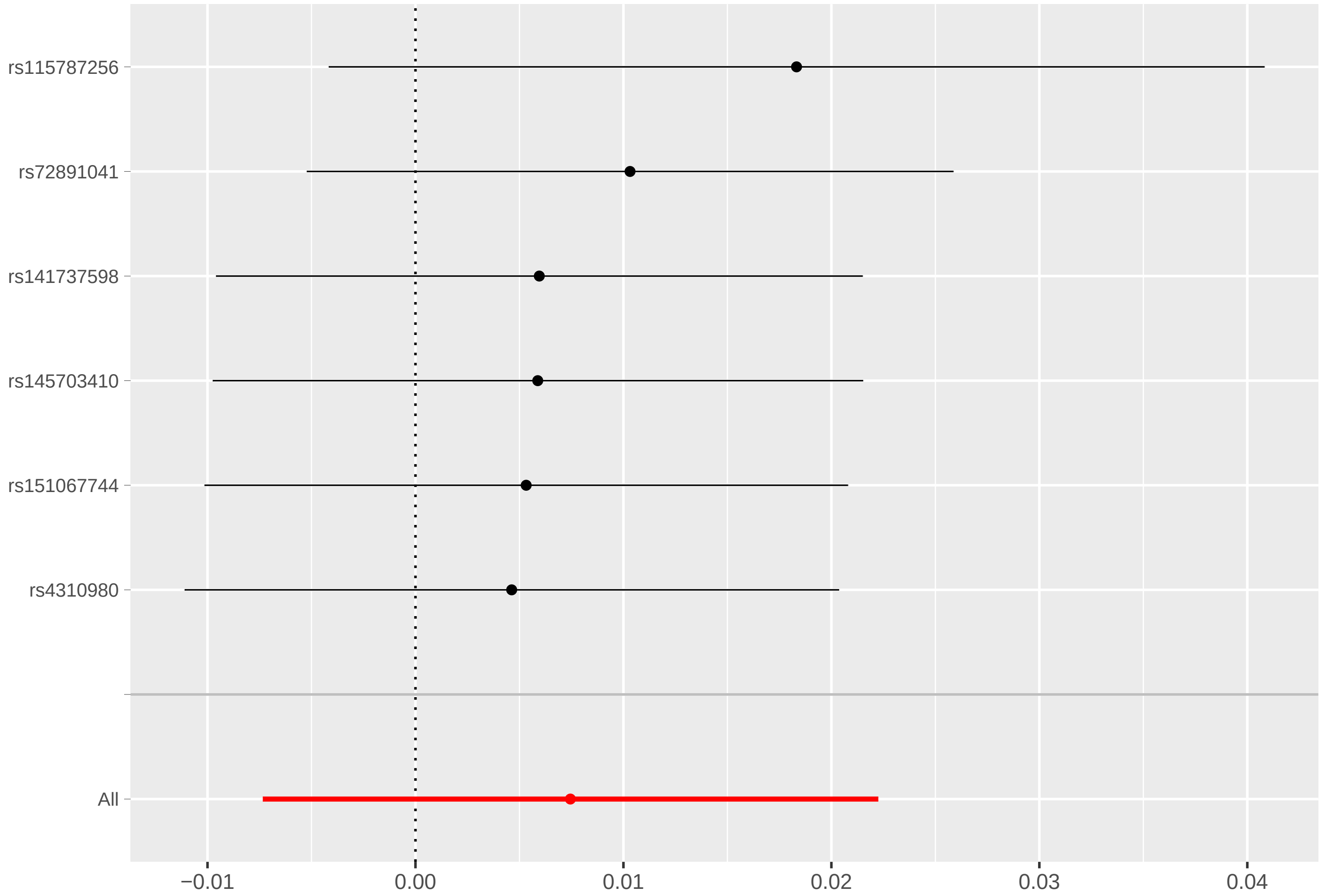

MR leave−one−out sensitivity analysis for
' || id:finn−b−SFN' on 'Bipolar disorder bip2021 || id:ieu−b−5110'

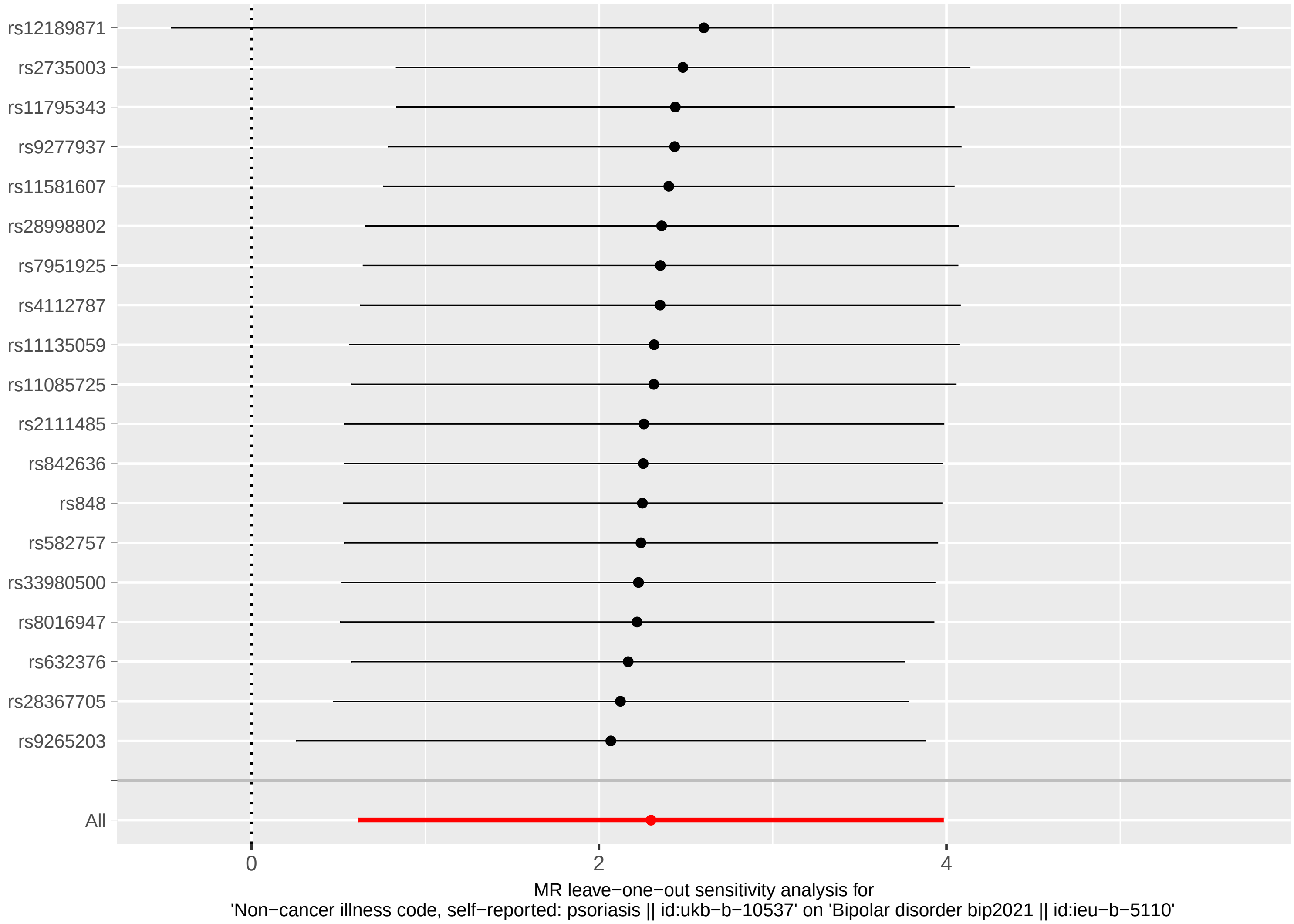

rs12189871
rs2735003
rs11795343
rs9277937
rs11581607
rs28998802
rs7951925
rs4112787
rs11135059
rs11085725
rs2111485
rs842636
rs848
rs582757
rs33980500
rs8016947
rs632376
rs28367705
rs9265203
All
0
2
4
MR leave−one−out sensitivity analysis for
'Non−cancer illness code, self−reported: psoriasis || id:ukb−b−10537' on 'Bipolar disorder bip2021 || id:ieu−b−5110'

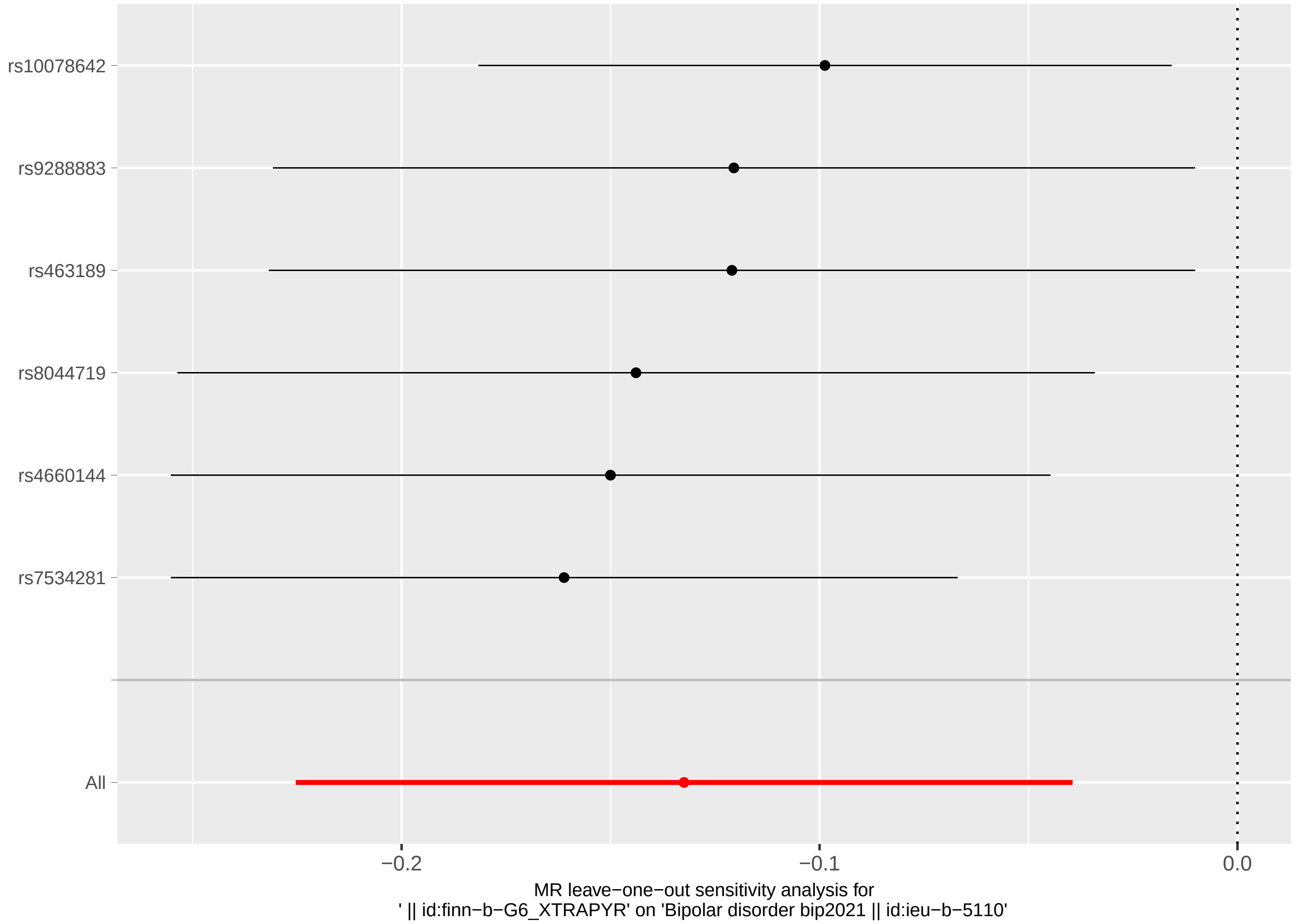
rs10078642
rs9288883
rs463189
rs8044719
rs4660144
rs7534281
All
−0.2
−0.1
0.0
MR leave−one−out sensitivity analysis for
' || id:finn−b−G6_XTRAPYR' on 'Bipolar disorder bip2021 || id:ieu−b−5110'

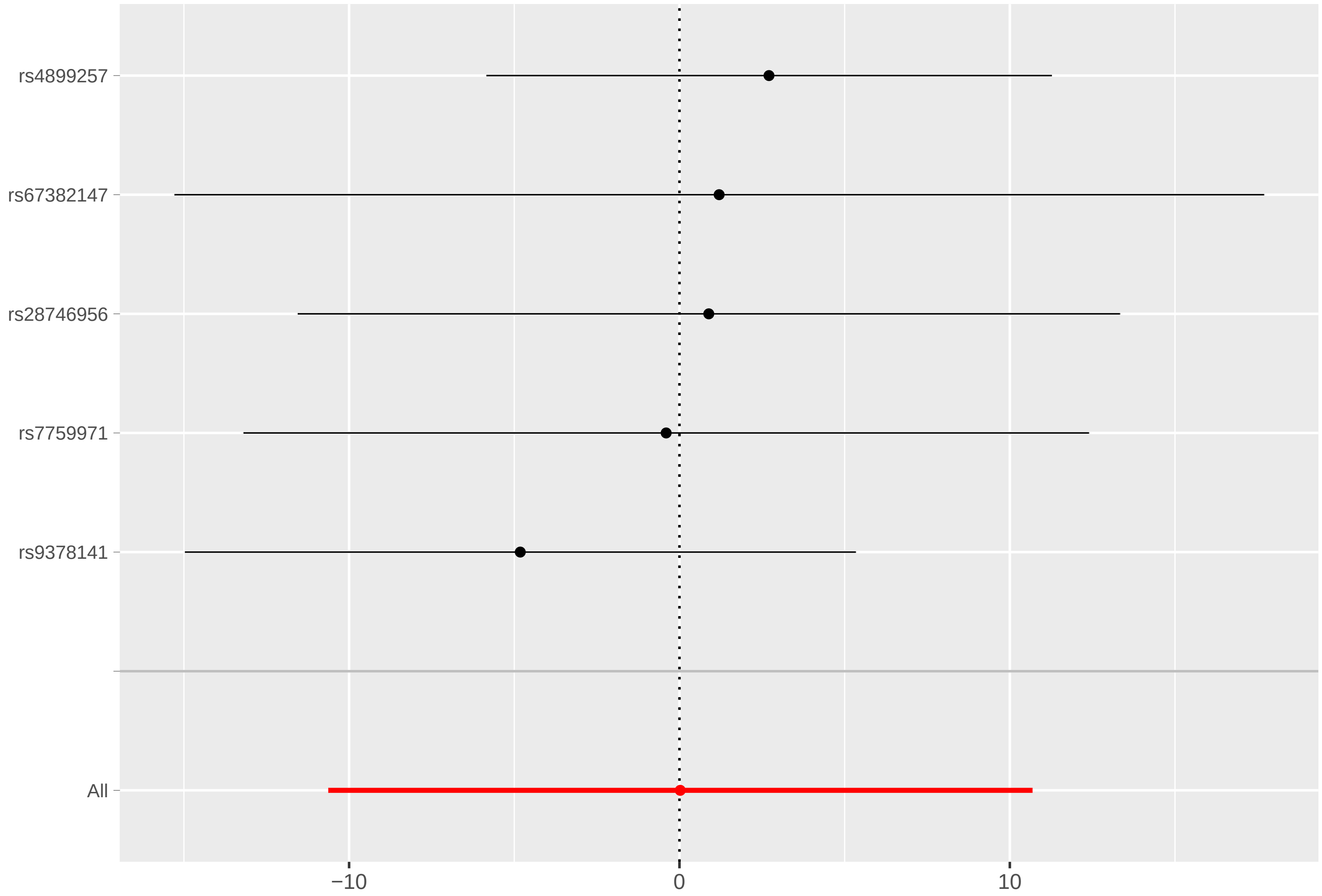

MR leave−one−out sensitivity analysis for
'Non−cancer illness code, self−reported: multiple sclerosis || id:ukb−b−17670' on 'Bipolar disorder bip2021 || id:ieu−b−5110'

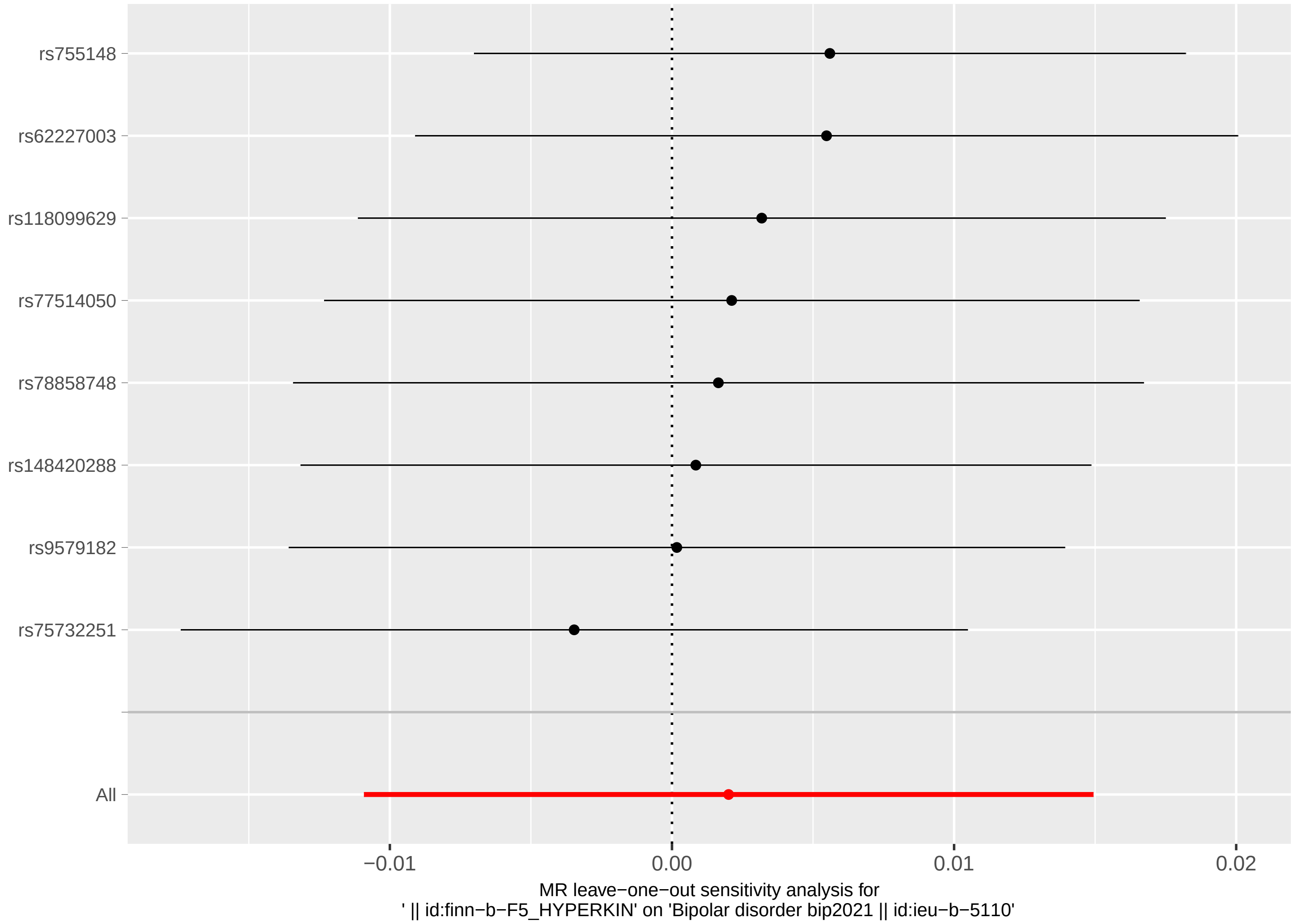
rs755148
rs62227003
rs118099629
rs77514050
rs78858748
rs148420288
rs9579182
rs75732251
All
−0.01
0.00
0.01
0.02
MR leave−one−out sensitivity analysis for
' || id:finn−b−F5_HYPERKIN' on 'Bipolar disorder bip2021 || id:ieu−b−5110'

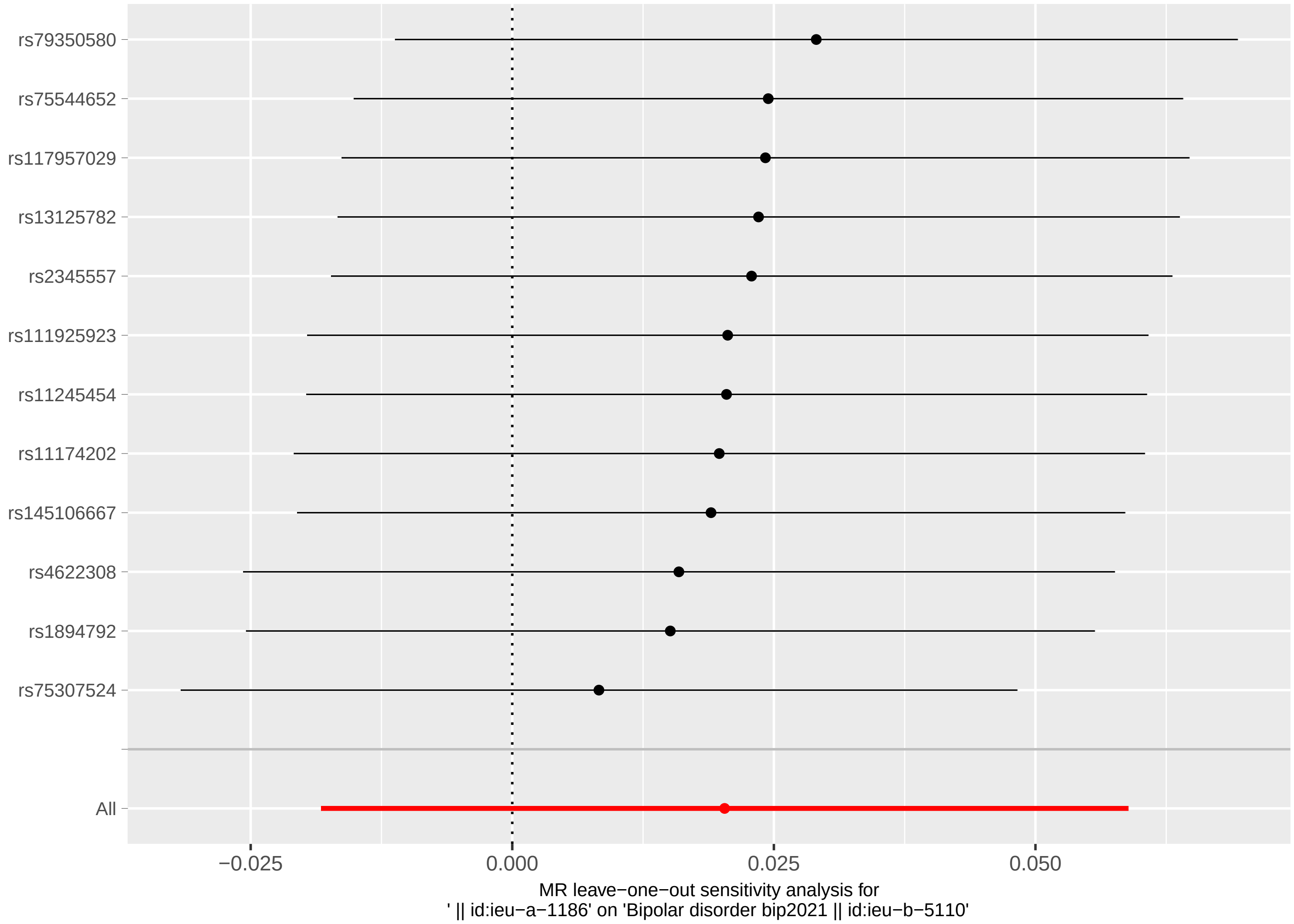
rs79350580
rs75544652
rs117957029
rs13125782
rs2345557
rs111925923
rs11245454
rs11174202
rs145106667
rs4622308
rs1894792
rs75307524
All
−0.025
0.000
0.025
0.050
MR leave−one−out sensitivity analysis for
' || id:ieu−a−1186' on 'Bipolar disorder bip2021 || id:ieu−b−5110'

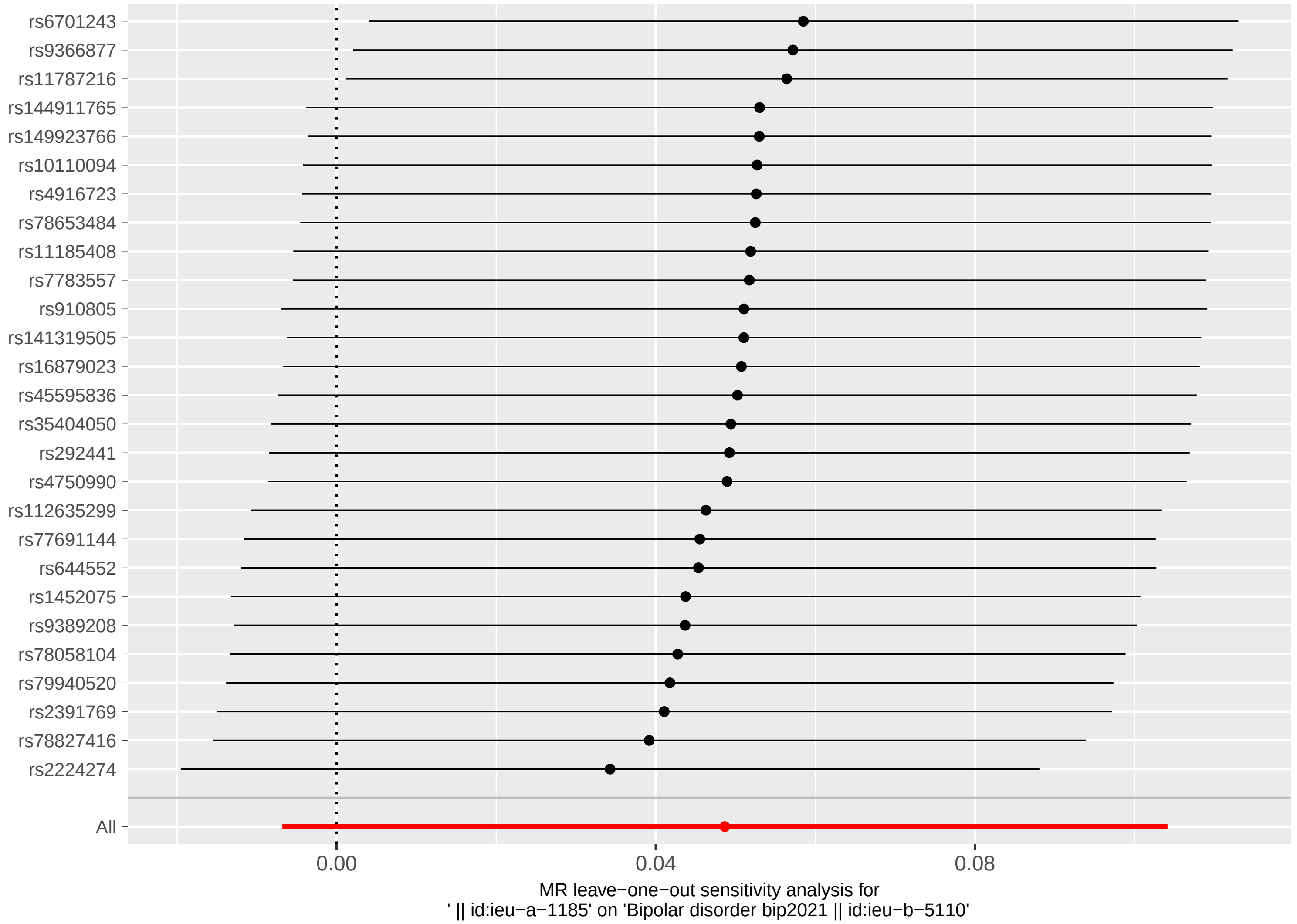
rs6701243
rs9366877
rs11787216
rs144911765
rs149923766
rs10110094
rs4916723
rs78653484
rs11185408
rs7783557
rs910805
rs141319505
rs16879023
rs45595836
rs35404050
rs292441
rs4750990
rs112635299
rs77691144
rs644552
rs1452075
rs9389208
rs78058104
rs79940520
rs2391769
rs78827416
rs2224274
All
0.00
0.04
0.08
MR leave−one−out sensitivity analysis for
' || id:ieu−a−1185' on 'Bipolar disorder bip2021 || id:ieu−b−5110'

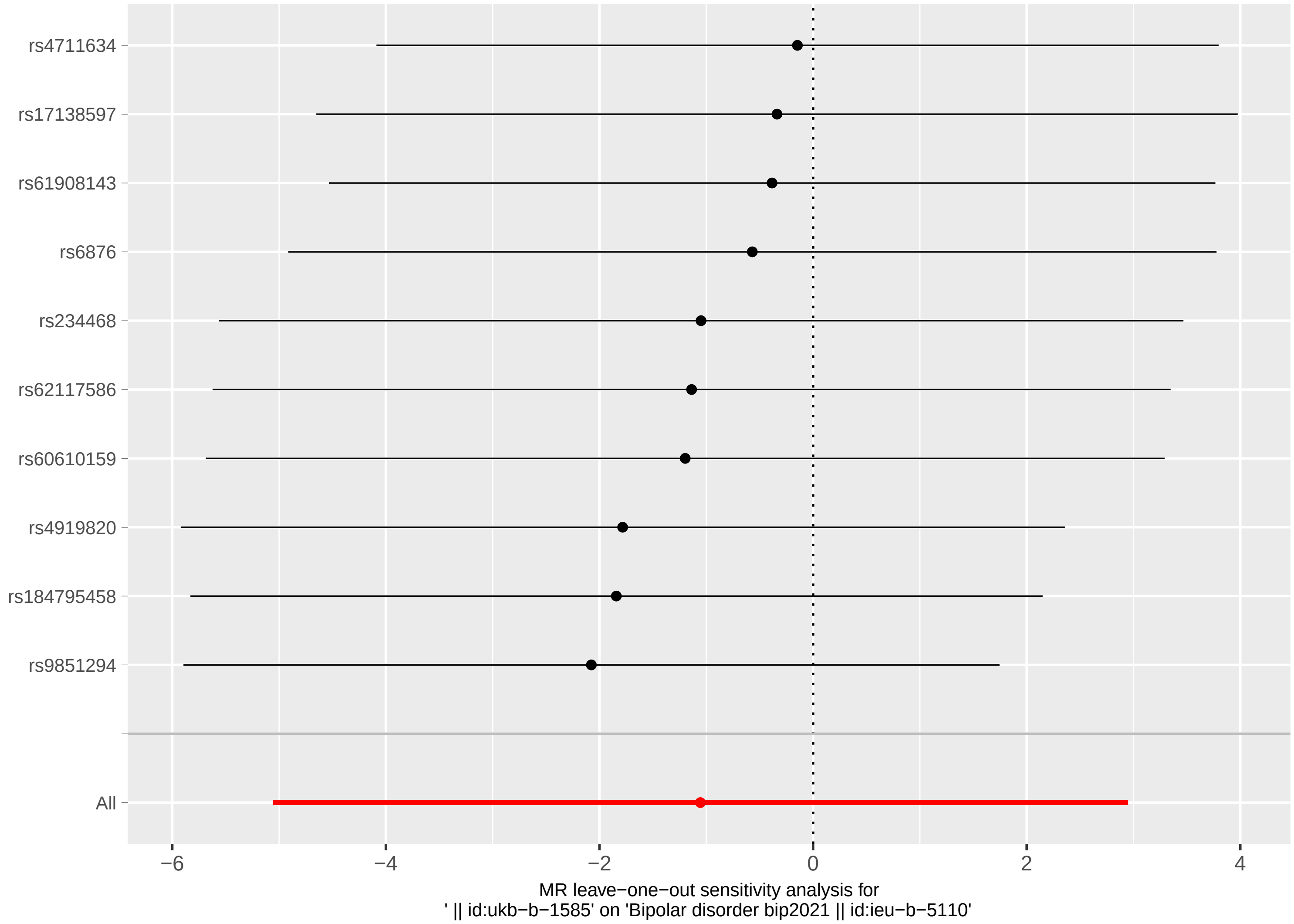
rs4711634
rs17138597
rs61908143
rs6876
rs234468
rs62117586
rs60610159
rs4919820
rs184795458
rs9851294
All
−6
−4
−2
0
2
4
MR leave−one−out sensitivity analysis for
' || id:ukb−b−1585' on 'Bipolar disorder bip2021 || id:ieu−b−5110'
